# Supplementary material for: Medication incidents in primary care medicine: a prospective study in the Swiss Sentinel Surveillance Network (Sentinella)
Source: BMJ Open. 2017 Jul 26;7(7):e013658. doi: 10.1136/bmjopen-2016-013658 (PMC5642752; doi:10.1136/bmjopen-2016-013658)
Supplement: Supplementary data [file bmjopen-2016-013658supp001.pdf]

Appendix A Incident questionnaire

Medication Incidents in Primary Care (MIPC)
(Version 1.0, January 25th 2015) (Internet version not available in English)

Incident reporting form

Administrative Information

1. Sentinella identification number: ☐☐☐☐ **2. Week of reporting:** ☐☐

The Patient

3. Year of birth: ☐☐☐☐ **4. Gender:** m ☐ f ☐

5. What was your relationship to the patient when the incident happened? Were you the

family physician ☐ emergency / substitute physician ☐ institution physician ☐ other ☐
if other what kind………………………

6. What is the patient's living situation?

with partner / family ☐ alone ☐ institution ☐ unknown ☐

7. Are there social problems?
yes ☐ no ☐ unknown ☐

8. Is the patient demented or otherwise mentally handicapped?

yes ☐ no ☐ unknown ☐

9. Does he suffer from psychological problems?

yes ☐ no ☐ unknown ☐

10. Does he take psychotropic drugs (esp. antidepressants, neuroleptics, benzodiazepines, opiates)?

yes ☐ no ☐ unknown ☐

11. Are there linguistic problems?

yes ☐ no ☐ unknown ☐

12. Does he or she smoke?

yes ☐ no ☐ unknown ☐

13. Is there substance abuse (other than nicotine)?

yes ☐ no ☐ unknown ☐**,** if yes, what substance? ……………………..

14. Does the patient have uncorrected / uncorrectable visual impairment?

yes ☐ no ☐ unknown ☐

15. Does the patient have uncorrected / uncorrectable hearing impairment?

yes ☐ no ☐ unknown ☐

16. Does the patient have uncorrected / uncorrectable mobility impairment?

yes ☐ no ☐ unknown ☐

17. Is there renal insufficiency (GFR: <60 ml/min/1.73 m^2)?

yes ☐ no ☐ unknown ☐

18. Is there hepatic insufficiency or liver cirrhosis?

yes ☐ no ☐ unknown ☐

19. Was the patient hospitalized in the past 12 months?
yes ☐ no ☐ unknown ☐

20. Is the patient taken care of by others? (*only one answer permitted*)?

yes, family / proxies ☐ yes, community nurse ☐ yes, institution ☐ no ☐ unknown ☐

21. Number of regularly applied active substances (including non-daily applied ones, see guidelines)?

☐☐ unknown ☐

22. Number of chronic conditions (see guidelines)?

☐☐ unknown ☐

23. Scale value of „Thurgau Morbidity Index" (chronic part, see guidelines)?

☐ unknown ☐

Details of the incident

24. Please, give a short **description** of the incident (in block letters):

25. Who observed the incident? (*multiple answers possible*)?

Physician / staff ☐ patient / relatives ☐ community nurse ☐ home / institution ☐ hospital ☐

other physicians ☐ pharmacist ☐ other ☐ unknown ☐

for „other” please specify: ……………………………

26. What happened (*multiple answers possible*)?

- dosage too high ☐
- dosage too low ☐
- application too short ☐
- application too long ☐
- wrong administration route ☐
- wrong medication ☐
- indicated medication not received ☐
- expired / defective medication ☐
- problems with insurance reimbursement ☐

- unclear / undefined ☐
- other (please specify): ……………………… ☐

27. Please state the **trade name** of the medication used in the incident:

………………………………………………..

28. Please note other medication names, presuming they are relevant to the case.

none ☐ unknown ☐

29. How would you judge the degree of hazard to the patient during the incident?

mild ☐ medium ☐ severe ☐ none ☐ does not apply ☐ unknown ☐

30. How intense was the impairment (as judged by the patient)?

mild ☐ medium ☐ severe ☐ fatal ☐ no symptoms, but pathological lab values ☐
no impairment ☐ does not apply ☐ unknown ☐
If there wasn't *any* impairment, please skip to **question 34**.

31. How long did the impairment last?

hours ☐ days ☐ weeks ☐ longer ☐ unknown ☐

32. How was the recovery?
without residues ☐ with mild residues ☐ with severe residues / fatal ☐ unknown ☐

33. Which organ system was affected (*multiple answers possible*)?

cardiovascular	☐
central nervous	☐
gastro-enteral	☐
kidneys	☐
liver	☐
lung	☐
skin	☐
other, please specify ………………….	☐

34. Did you have to apply a specific surveillance or treatment for the incident?

yes, ambulatory ☐ yes, hospital ☐ no ☐ unknown ☐

If yes, please specify which? ……………………………………………..

35. What factors contributed causally to the emergence of the incident (*multiple answers possible*)?

off duty hours ☐

communication failure within practice ☐

generic substitution by pharmacist ☐

hand-written prescription incorrectly interpreted ☐

conflicting multiple prescriptions ☐

lack of alertness within practice ☐

lack of documentation ☐

insufficient patient instruction ☐

lack of aids (e.g. Dosette®) ☐

lack of cooperation by patient / relatives ☐

misleading package leaflet information ☐

patient's internet search ☐

administrative problems ☐

manufacturer related (defective medication) ☐

distributer related (out of stock) ☐

lack of maintenance (e.g. first aid kit) ☐

other, please specify……………………….. ☐

unknown ☐

36. Was there an interface problem? If yes, which (*multiple answers possible*)?

- yes, with hospital ☐
- yes, with institution ☐
- yes, with community nurse ☐
- yes, with pharmacist ☐

yes, with specialist physician ☐
yes, with other, please specify? …………… ☐

no ☐

If **no**, please skip to **question 38**.

37. Was there an **explicit comparison** of prescription lists with the institution / person?

yes, verbal / by phone ☐ yes, written / by fax ☐ no ☐ unknown ☐

38/39. Was the patient informed about the incident?

yes, by myself / practice staff ☐
no, because he was not able to understand the message (children, demented) ☐
no, because the problem was solved and communication would have impaired confidence ☐
no, because the patient had moved or was deceased ☐
no, this was not needed because patient / relatives themselves had observed the incident ☐
no, because others had already informed him ☐

no, because: ………………………………………………………………………. ☐
unknown ☐

If yes, what was the patient’s reaction? ……………………………………………………

40. What did you do as a result to prevent similar incidents in the future (*multiple answers possible*)?

change standard operations procedures ☐
better instruction of patients ☐
communication with institution(s) ☐
notification of manufacturer ☐
notification of liability insurer ☐
notification of drug authority („yellow leaflet“) ☐
notification of the “critical incident reporting system” ☐
other, please specify? ……………………….. ☐
nothing ☐

41. Who or what was ultimately **responsible** for the occurrence of the incident? ……………………………………………

42. In the given situation, do you think one could have **anticipated** the event?

yes ☐ no ☐

43. Did you already report an **identical** or very similar incident to this study?

yes ☐ no ☐

44. Please make any **suggestions** about the kind of measures that could be taken to generally reduce the frequency of such events (in block letters):

Please keep a copy of this questionnaire in the patient files. Thank you for filling it out!
