# Supplementary material for: Medication incidents in primary care medicine: a prospective study in the Swiss Sentinel Surveillance Network (Sentinella)
Source: BMJ Open. 2017 Jul 26;7(7):e013658. doi: 10.1136/bmjopen-2016-013658 (PMC5642752; doi:10.1136/bmjopen-2016-013658)
Supplement: Supplementary data [file bmjopen-2016-013658supp002.pdf]

Appendix B

Swiss Pharmaceutical Market: Number of packaged sold by ATC-groups, 2015

	2015<br>number of packa-ged sold
Total	210,992,389
A Alimentäres System und Stoffwechsel	31,455,252
A01 Stomatologika, Medizinische Präparate zur Mund- und Zahnpflege	1,473,520
A02 Antacida-Ulcustherapheutika, Antiflatulentia	6,051,251
A02A Antacida,Antiflatulentia	1,500,822
A02B Ulcustherapeutika	4,426,496
A02C Sonstige Magentherapeutika	123,933
A03 Produkte gegen funktionelle Magen-Darm-Störungen	2,087,299
A03A Antispasmodika+Anticholinergika rein	595,861
A03C Antispasmodika/Ataraktika-Kombinationen	25,411
A03E Antispasmodika, sonstige Kombinationen	222,102
A03F Gastroprokinetika	1,233,461
A03G Modulatoren der gastrointestinalen Sensomotorik	10,464
A04 Antiemetica und Antinausea	920,841
A05 Cholagoga und Leberschutz-Mittel	337,970
A05A Gallentherapeutica und Cholagoga	254,744
A05B Leberschutzpräparate	83,226
A06 Mittel gegen Verstopfung und Darmreinigung	4,583,118
A06A Mittel Gegen Verstopfung	4,329,037
A06B Darmreinigungsmittel	254,081
A07 Antidiarrhoica, Elektrolytzufuhr und intestinale Antiphlogistica	3,005,035
A07B Intestinale Adsorbierende Antidiarrhoica	228,076
A07E Intestinale Antiphlogistica	222,680
A07F Antidiarrhoica Micro-Organismen	1,340,432
A07G Orale Elektrolyt-Zufuhr	107,030
A07H Motilitätshemmer	1,095,216
A07X Übrige Antidiarrhoica inkl. A07A Antidiarrhoica intestinale Antiinfectiva	11,601
A08 Antiadiposita excl. Diätetica	51,654
A09 Digestiva und Enzyme	804,667
A10 Antidiabetica	3,376,580
A10C Humaninsulin und Analoga	1,078,495
A10H Sulfonylharnstoff Antidiabetika	321,219
A10J Biguanid Antidiabetika	1,217,578
A10K Glitazone Antidiabetika	25,305
A10M Glinide Antidiabetika	38,782
A10N DPP-IV Inhibitor Antidiabetika	483,559
A10P SGLT2-Hemmer Antidiabetika	45,927
A10S GLP-1 Agonisten Antidiabetika	156,656
A10X Übrige Antidiabetika inkl. Insulin tierischen Ursprungs und Alphagluko-sidaseinhibitor Antidiabetika	8,616

A11 Vitamine	5,001,408
A11A Multivitamine mit Mineralstoffen	755,243
A11B Multivitamine ohne Mineralstoffe	17,507
A11C Vitamin A+D inkl. einfache Kombinationen	2,618,699
A11D Vitamin B1 und Kombinationen	170,884
A11E Vitamin B-Komplex	739,860
A11F Vitamin B 12 rein	123,445
A11G Vitamin C, inkl. Kombinationen mit Mineralstoffen	353,644
A11X Sonstige Vitamine	222,126
A12 Mineralverbindungen	3,490,891
A12A Mineralverbindung Calcium	1,254,334
A12B Mineralverbindung Kalium	200,438
A12C Sonstige Mineralverbindungen	2,036,119
A13 Tonica und Roborantia	229,735
A16 Übrige Präparate des alimentären und Stoffwechsel-Systems	34,295
B Blut und Blutbildende Organe	5,507,624
B01 Antithrombotika	4,009,465
B01A Vitamin-K-Antagonisten	399,963
B01B Heparine	714,872
B01C Thrombozytenaggregationshemmer	2,265,838
B01D Fibrinolytika	14,293
B01X Direkte Thrombin-Inhibit und direkte Faktor XA-Hemmber	598,766
B02 Blutgerinnungssystem – Sonstige Produkte	237,495
B02B Antagonisten (Antidot.Anticoag)	146,201
B02D Blutcoagulation	1,805
B02G Glitazone-Antidiabetika	48,969
B02X Übrige Blutgerinnungsprodukte (Tissues sealing preparations, Thrombopoietin Agonisten, Antifibrinolytica synthetisch etc.)	40,520
B03 Antianämica	1,259,896
B03A Antianämica mit Eisen und Kombinationen	862,744
B03C Erythropoietin-Präparate	67,258
B03X Sonstige Antianämica inkl. Folsäure/Folinsäure	329,894
C Herz-Kreislauf Therapie	16,027,143
C01 Cardica	954,962
C01B Anti-Arrhythmie-Präparate	213,952
C01C Cardiale Stimulantien excl. Herzglykoside	266,028
C01D Coronartherapeutica excl. Calcium-Antagonisten und Nitropräparate	121,815
C01E Nitropräparate und Analog	228,438
C01X Positive Inotrope, Herzglykoside und übrige Herzpräparate	124,729
C02 Antihypertonica	129,957
C03 Diuretica	1,815,738
C04 Cerebrale und periphere Vasotherapeutika	390,255
C05 Antivaricosa/Antihaemorrhoidalia	2,271,175
C05A Antihaemorrhoidalia topisch	773,800
C05B Antivaricosa topisch	1,029,010
C05C Antivaricosa systemisch	468,365
C06 Übrige Herz+Kreislaufmittel	339,930
C07 Betablocker	2,398,309
C07A Betablocker rein	2,272,988

C07B Betablocker Kombination	125,321
C08 Calciumantagonisten	1,157,267
C09 Stoffe mit Wirkung auf das Renin-Angiotensin-System	4,116,677
C09A Ace-Hemmer, rein	1,142,465
C09B Ace-Hemmer, Kombinationen	584,697
C09C Angiotensin-II-Antagonisten, rein	1,084,218
C09D Angiotensin-II-Antagonisten, Kombinationen	1,261,616
C09X Sonstige Stoffe mit Wirkung auf das Renin - Angiotensin - System	43,681
C10 Lipidregu/Antiarteriosklerotika	2,446,927
C10A Lipidregulatoren	2,341,963
C10A1 Statine (Cholesterolsynthesehemmer)	2,190,227
C10A2 Lipidsenker, Fibrate, Ionenaustauscher und sonstige Lipidsenker	151,736
C10C Lipidregulatoren kombiniert mit anderen Lipidregulatoren	104,964
C11 Übrige Präparate für die Herz-Kreislauf-Therapie	5,946
D Dermatologica	17,314,810
D01 Dermatologische Antimykotica	1,360,570
D02 Emollientia und Hautschutzmittel	2,703,273
D03 Wundheilmittel	4,389,791
D04 Antipruriginosa inkl. Antihistaminica und Anästhetica usw.	1,458,276
D05 Nichtsteroidale Präparate gegen entzündliche Hauterkrankungen	327,245
D06 Topische antibakterielle Produkte und antivirale Mittel	864,829
D06A Topische antibakterielle Produkte	379,013
D06D Topische Produkte gegen Virusinfektionen	485,816
D07 Corticosteroide topisch	1,982,173
D07A Reine Corticosteroide topisch	960,182
D07B Corticosteroid-Kombinationen topisch	1,021,991
D08 Antiseptica + Desinfizientia	1,836,788
D10 Aknemittel	1,227,850
D10A Topische Aknemittel	829,114
D10B Orale Aknemittel	398,736
D11 Übrige Dermatologica	1,164,015
G Urogenital-System+Sexualhormon	7,936,641
G01 Gynaekologische Antiinfektiva	812,985
G01A Trichomonadenmittel	185,395
G01B Gynaekologische Antimykotica	475,911
G01X Gynaekologische antibakterielle Produkte und Antiseptika	151,679
G02 Sonstige Gynaekologica	1,060,376
G02A Oxytocica	91,111
G02F Topische Sexualhormone	496,735
G02X Lokale Antikonzipientia, Prolactininhibitoren, wehenhemmende Mittel und sonstige Gynaekologika	472,530
G03 Sexualhormone und Stimulantien des Genitalsystems	3,917,793
G03A Hormonale Kontrazeptiva systemisch	2,477,919
G03B Androgene, excl.G3E,G3F	70,852
G03C Oestrogene, excl.G3A,G3E,G3F	397,050
G03D Progestogene, excl. G3A,G3F	245,149
G03F Oestrogen-Progestogen-Kombinationen	487,291
G03G Gonadotropin inl. sonstige Ovulationsstimulantien	100,879

G03X Androgen-Kombinationen mit weiblichen Sexualhormonen, SERMS und sonstige Sexualhormone und ähnliche Produkte	138,653
G04 Urologica	2,145,487
G04A Urologische Antiinfektiva und Harnantiseptika	410,545
G04C BPH Produkte (Benigne Prostata-Hyperplasie)	715,845
G04D Harninkontinenzprodukte	390,832
G04E Produkte gegen Erektionsstörungen	311,659
G04X Alle sonstigen Urologika	316,606
H Hormonales System, exkl. Sexualhormone	2,875,760
H01 Hypophysen-/Hypothalamus-Hormone	36,944
H02 Corticosteroide Systemisch	1,575,880
H03 Schilddruesentherapie	1,109,934
H04 Sonstige Hormone (wachstumshormone, antidiuretische Hormone, Anti-Parathyroid-Hormone etc.)	153,002
J Antiinfektiva systemisch	8,446,623
J01 System.Antibakter.Prod.	5,721,173
J01A Tetracycline und Kombinationen	155,659
J01C Breitspektrum Penicilline	2,353,136
J01D Cephalosporine	897,839
J01F Macrolide und vergleichbare Substanzen	778,785
J01G Fluorchinolone	817,056
J01H Mittel- und Schmalspektrum-Penicilline	111,484
J01K Aminoglykoside	46,447
J01P Sonstige Beta-Lactam. Antibiot. Penicilline, Cephalosporine (Monobactame, Peneme und Carbapeneme)	69,074
J01X Rifamycin und vergleichbare Substanzen, Trimethoprim, Kombinationen und vergleichbare Substanzen und sonstige antibakterielle Produkte	491,693
J02 Antimykotika, Systemisch	591,346
J04 Tuberkulosemittel	37,320
J05 Antivirale Mittel, systemisch	570,063
J05B Antivirale Mittel, exkl. Produkte gegen HIV	236,676
J05C Virustatika gegen HIV	333,387
J05C1 Nucleoside und Nucleotide Reverse Transkriptase Hemmer	108,107
J05C2 Protease Hemmer	98,581
J05CX Nicht-Nucleoside Reverse Transkriptase Hemmer, HIV Fusionshemmer, HIV-Antivirale Integrase Hemmer und sonst.HIV-Antivirale Mittel	126,699
J06 Sera und Gammaglobulin	23,164
J07 Vakzine	1,406,674
J07B Vakzine Kombinationen	463,631
J07B1 Kombinationen mit Tetanusimpfstoff	387,139
J07BX Mehrfach-Impfstoffe mit Masern und/oder Mumps und alle anderen Kombinationen	76,492
J07D Bakterielle Impfstoffe	164,542
J07E Virale Impfstoffe	687,366
J07E1 Influenza-Impfstoff	205,391
J07E4 Hepatitis-Impfstoff	212,503
J07E9 Alle uebrigen viralen Impfstoffe, inkl. HPV, Rotaviren, Varizellen etc.	269,472
J07X Alle uebrigen Impfstoffe und aehnliche Produkte	91,135
J08B Übrige Antiinfektiva	96,883
K Infusionslösungen	24,158,749
K01 Intravenoese Lösungen ab 100 ML	12,064,154

K01A Elektrolytlösungen	3,372,213
K01B Standardlösungen	8,461,628
K01D Fettemulsionen, inkl. Produkte zur totalen parenteralen Ernährung	156,484
K01E Aminosäuren-Lösungen	51,140
K01X Kalorische Lös. über 10% und Lösungen zur Osmotherapie	22,689
K02 Plasma Expander	70,547
K03 Blut- und Blutersatzinfusionslösungen	1,817
K04 Injektionslösungen/Infusionszusätze	12,005,621
K04A Elektrolytlösungen	752,499
K04B Standardlösungen	9,981,186
K04C Kalorische Lösungen < 100 ML	34,050
K04D Sonstige Injektionslösungen/Infusionszusätze < 100 ML	1,237,886
K05 Spüllösungen	16,610
L Antineoplastika und Immunmodulatoren	1,934,950
L01 Antineoplastika	984,072
L01A Alkylisierende Substanzen	46,698
L01B Antimetaboliten	323,741
L01C Vinca-Alkaloide & sonstige pflanzliche Produkte	157,793
L01D Antineoplastisch wirkende Antibiotika	46,619
L01F Platinhalt.Antineoplast.	95,347
L01G Monoklonale Antikörper zur antineoplastischen Therapie	159,208
L01H Proteinkinasehemmer zur antineoplastischen Therapie	33,895
L01X Sonstige Antineoplastika	120,771
L02 Cytostatische Hormonetherapie	183,379
L02A Cytostatische Hormone	43,050
L02B Cytostatische Hormonantagonisten	140,329
L03 Immunostimulantien	79,294
L03A Immunstimulantien	40,990
L03B Interferone	38,304
L04 Immunsuppressiva	688,205
L04B Anti-TNF-Produkte	340,857
L04X Sonstige Immunsuppressiva inkl. Interleukin Inhibitoren	347,348
M Muskel-und Skelettsystem	14,787,413
M01 Antirheumatica	6,373,707
M01A Corticoidfreie Antirheumatica	5,700,326
M01C Antirheumatica spezifisch	673,381
M02 Rheumaeinreibungen Rubefatientia	6,372,249
M03 Muskelrelaxantien	665,391
M03A Muskelrelaxantien peripher wirkend	85,757
M03B Muskelrelaxantien zentral wirkend	579,634
M04 Gichtmittel	368,020
M05 Übrigige Muskel-Skelettsystem-Präparate	1,008,046
N Nervensystem	42,690,195
N01 Anaesthetica	1,554,391
N01A Narkosemittel	1,078,794
N01A1 Narkosemittel Inhalationspräparate	20,582
N01A2 Narkosemittel Injektionspräparate	1,058,212
N01B Lokalanaesthetika	475,597

N01B1 Medizinische injizierbare Lokalanästhetika	198,964
N01B3 Topische Lokalanästhetika	270,962
N01B9 Sonstige Lokalanästhetika und dentale injizierbare Lokalanästhetika	5,671
N02 Analgetica	24,080,469
N02A Betäubungsmittel	1,661,607
N02B Analgetica und Antipyretica	21,918,098
N02B1 Analgetica rezeptpflichtig	8,592,792
N02B2 Analgetica rezeptfrei	13,325,306
N02C Migränemittel	500,764
N02C1 Triptane	432,741
N02C9 Sonstige Migränemittel	68,023
N03 Antiepileptica	1,957,745
N04 Parkinsonmittel	658,710
N05 Psycholeptica	9,075,955
N05A Antipsychotika	2,002,404
N05A1 Atypische Antipsychotika	1,521,618
N05A9 Konventionelle Antipsychotika	480,786
N05B Hypnotica und Sedativa	4,462,062
N05B1 Hypnotica und Sedativa, barbituratfreie Reinsubstanzen	2,830,382
N05B2 Hypnotica und Sedativa, barbituratfreie Kombinationen	129,461
N05B5 Pflanzliche Hypnotica und Sedativa	1,502,219
N05C Tranquilizer	2,611,489
N06 Psychoanaleptica Ex.Antiadipos	4,081,575
N06A Antidepressiva und stimmungsstabilisierende Produkte	3,503,509
N06A2 Pflanzliche Antidepressiva	245,372
N06A3 Stimmungsstabilisierende Produkte	50,396
N06A4 SSRI Antidepressiva	1,319,897
N06A5 SNRI Antidepressiva	664,070
N06A9 Sonstige Antidepressiva	1,223,774
N06B Psychostimulantia	341,172
N06X Psycholeptica-Psychoanaleptica-Kombinationen, Nootropica und Neurotonica sowie verschiedene andere Produkte	236,894
N07 Sonstige ZNS-wirksame Produkte	1,281,350
N07B Raucherentwöhnungsmittel	652,146
N07C Produkte gegen Schwindel	286,538
N07D Anti-Alzheimer Produkte	121,928
N07E Alkohol- und Opiatentwöhnungsmittel	126,973
N07X Alle anderen ZNS-wirksamen Präparate	93,765
P Parasitologie	462,559
P01 Antiprotozoenmittel und Anthelmintika	437,402
P01B Anthelmintica, excl. Schistosomiasis-Mittel	296,327
P01D Malariamittel	139,242
P02 Sonstige Antiparasitäre Präparate inkl. Insektizide und Repellentien	26,990
R Respirationssystem	28,837,468
R01 Rhinologica	7,246,454
R01A Rhinologica topisch	6,868,431
R01AX Corticosteroide Rhinologika	1,067,991
R01A6 Antiallergische Rhinologica	230,180
R01A7 Abschwellende Rhinologica	4,290,641

R01A9 Sonstige Rhinologica topisch inkl. Antiinfektive Rhinologika ohne Corticosteroide	1,279,619
R01B Rhinologica systemisch	378,023
R02 Halsschmerzmittel und Antiinfectiva	3,977,806
R03 Antiasthmatika und Produkte gegen chronisch-obstruktive Lungenerkrankungen	2,931,292
R03A B2-Stimulatoren	923,570
R03B Xanthine	16,188
R03C Degranulationshemmer	9,088
R03D Corticoide	258,075
R03F Komb. B2-Stimulatoren mit Corticoiden	1,172,896
R03G Anticholinergica rein und in Komb. mit B2-Stimulatoren	422,479
R03G3 Anticholinergica rein, Inhalate	269,418
R03G4 Anticholinergica – Kombinationen mit Beta-2-Stimulantien, Inhalate	153,061
R03J Antileukotrien Antiasthmatika	90,062
R03X Sonstige Antiasthmatika und Produkte gegen chronisch-obstruktive Lungenerkrankungen inkl. PDE4-Hemmer gegen Asthma / Chronisch-Obstruktive Lungenerkrankungen	38,934
R04 Percutane Einreibemittel und Inhalationspräparate	1,093,346
R05 Husten- und Erkältungspräparate	10,899,026
R05A Erkältungspräparate	2,498,594
R05C Expectorantia ohne Antiinfectiva	4,499,364
R05D Hustensedativa	3,297,144
R05D1 Hustenmittel plain (Einzelsubstanzen)	1,971,766
R05D2 Sonstige Hustensedativa, inkl. Kombinationen	1,325,378
R05F Sonstige Husten- und Erkältungspräparate	603,924
R06 Antihistamine systemisch	2,687,483
R07 Sonstige Präparate des Respirationssystems	2,061
S Sinnesorgane	7,658,311
S01 Ophthalmologica	7,214,097
S01A Ophtalmologische Antiinfectiva	451,604
S01B Ophtalmologische Corticoide rein	271,663
S01C Ophthalm. Antiphlogistika und Antiinfektiva Kombinationen	613,682
S01E Myotika und Mittel zur Glaukombehandlung	1,038,592
S01F Mydriatika und Cykloplegika	46,547
S01G Ophthalmologische Antiallergika, Abschwellende Mittel, Antiseptika	1,473,188
S01H Ophtalmolog. Lokalanesthetika	40,383
S01K Künstliche Tränen und Netzmittel für Augen	2,503,750
S01P Ophthalmologische Antineovaskularisationsprodukte	127,501
S01R Nonsteroidale entzündungshemmende Ophthalmologica (NSAID'S)	236,207
S01X Übrige Ophtalmologica inkl. Präparate für den Gebrauch von Kontaktlinsen, Augentonika und Augenvitamine, Präparate zur Verhütung von Katarakt und Antikataraktogenika, Ophthalmologische Operationshilfsmittel und Ophthalmologische Diagnostika	411,009
S02 Otologica	444,214
T Diagnostika	43,184
T01 Diagnostika für bildgebende Verfahren	18,885
T02 Testdiagnostika	24,299
V Verschiedenes	855,707
V01 Allergene	6,429
V03 Übrige Therapeutische Präparate	820,500

V03D Entgiftungspräparate für die Cytostatikatherapie	45,668
V03E Antidote	4,532
V03G Hyperkaliämie/Hyperphosphatämie-Produkte	42,259
V03X Sonstige therapeutische Präparate inkl. Entzündungshemmende Enzyme, Eisen-Chelatbildner und Radiopharmazeutika	728,041
V05 Chirurgische Antiseptica	134
V06 Allgemeine Nährmittel	5,612
V07 Alle übrigen nicht therapeutischen Präparate	23,032

Source: Interpharma with data from IMS Health Schweiz, 2016.
