# Supplementary material for: Medication incidents in primary care medicine: a prospective study in the Swiss Sentinel Surveillance Network (Sentinella)
Source: BMJ Open. 2017 Jul 26;7(7):e013658. doi: 10.1136/bmjopen-2016-013658 (PMC5642752; doi:10.1136/bmjopen-2016-013658)
Supplement: Supplementary data [file bmjopen-2016-013658supp003.pdf]

Appendix C Initial questionnaire

Medication incidents in primary care (MIPC)

Initial reporting (Version 1.0, January 25th 2015)
Internet version: not available in English

1. Sentinella identification number: …………

2. Number of physicians within your practice: …………

3. Number of them who report to Sentinella …………

4. Your weekly workload, h/w: <16 ☐, 16-30 ☐, >30 ☐

5. Number of hours within your practice (%): <50% ☐, ≥50% ☐

6. Approximate proportion of medication prescribed (as compared to directly delivered drugs):
<33% ☐, 33-66% ☐, >66% ☐

7. Do you have an X-ray (machine?)? yes ☐ no ☐

8. Do you have an ECG? yes ☐ no ☐

9. Do you have an ultrasound? yes ☐ no ☐

10. Do you have an electronic system for controlling electronic drug interaction?
yes ☐ no ☐

11. Do you have electronic patient history documentation?
yes ☐ no ☐

12. Do you prescribe electronically?
yes, with a medication thesaurus ☐ yes, but without one (use of a typewriter) ☐ no ☐

13. Is your practice certified (e.g. EQUAM)? yes ☐ no ☐

14. Do you regularly schedule team sessions?
Yes, at least monthly ☐, yes, but less frequently ☐, no ☐

15. Do you attend quality circle sessions (in accordance with "Hausärzte Schweiz")?
yes, regularly ☐, yes, now and then ☐, no ☐

16. Did you complete a special education (e.g. manual or psychosomatic medicine), or do you have special interests (e.g. toxic maniac patients)? yes ☐, no ☐
if yes, please specify : …………………………………………………………………….

17. Are you contracted by an institution? yes ☐ no ☐
if yes, please specify (prison, home etc.): ………………………………………………………………

18. If yes, does this institution have specific problems with medication?
yes ☐, no ☐, if yes, please specify: ……………………………………………………………

19. Are you involved in other special activities (teaching, research, insurance doctor)?
yes ☐ no ☐
If yes, please specify the kind of activity: …………………………………………………….

Thank you very much!
