# Supplementary material for: Medication incidents in primary care medicine: a prospective study in the Swiss Sentinel Surveillance Network (Sentinella)
Source: BMJ Open. 2017 Jul 26;7(7):e013658. doi: 10.1136/bmjopen-2016-013658 (PMC5642752; doi:10.1136/bmjopen-2016-013658)
Supplement: Supplementary data [file bmjopen-2016-013658supp004.pdf]

Appendix D Final questionnaire

Medication Incidents in Primary Care (MIPC)

Final questionnaire (Version 3.0 / December 14th 2015)

Online version: not available in English

Dear colleagues,

Thank you for your dedicated support of our study on medication incidents in 2015, regardless of whether you reported much of them or not. We would like to ask some final questions which will help us put the other information you sent us in the right context.

1. Sentinella identification number: ……………

2. Did you **not** report medication incidents that you had noticed during the last year (e.g. because of lack of time)?

never or almost never ☐, yes, but rarely ☐, yes, frequently ☐ always or almost always ☐

If this was frequently the case, please explain why:………………………………………..

3. Did your practice participate in the fortnight morbidity denominator study in March 2015 (calendar weeks 11 and 12)?
yes, fully ☐, yes, *but only partly (by omission of certain variables)* ☐, no ☐

If you did not fully participate or not at all, what was the reason?
…………………………………………………….

If you did not participate in the denominator study, please continue with **question 12**.

4. How big was your effort for coding the morbidity variables of the denominator study?
manageable ☐, rather big ☐, too much ☐, impossible ☐

Did you have any difficulties when coding the morbidity variables?

5. Hospitalisation during the previous 12 months?	none ☐, a little ☐, considerable ☐, severe ☐
6. Care-dependency?	none ☐, a little ☐, considerable ☐, severe ☐
7. Number of medications?	none ☐, a little ☐, considerable ☐, severe ☐
8. Number of conditions?	none ☐, a little ☐, considerable ☐, severe ☐
9. Thurgau Morbidity Index?	none ☐, a little ☐, considerable ☐, severe ☐
10. Repeat consultation during the fortnight?	none ☐, a little ☐, considerable ☐, severe ☐

If you named considerable to severe difficulties in questions 5 to 10, please list them in **item 13**.

11. How long did it take for you and your practice nurse together for coding all of the variables of **one patient** (Dr. Gnädinger needed less than 3 minutes)?
………………………………………………. minutes

12. Would you be willing to be interviewed for a focus group on the subject of medication safety?

yes ☐, no ☐

13. Other comments:

Thank you very much!
