# Supplementary material for: Medication incidents in primary care medicine: a prospective study in the Swiss Sentinel Surveillance Network (Sentinella)
Source: BMJ Open. 2017 Jul 26;7(7):e013658. doi: 10.1136/bmjopen-2016-013658 (PMC5642752; doi:10.1136/bmjopen-2016-013658)
Supplement: Supplementary data [file bmjopen-2016-013658supp005.pdf]

Appendix E: electronic tables and figures

Figure e1: Distribution of the incident notifications over the year
Table e1: What went wrong with the incidents
Table e2: Organ system involved
Table e3: Causes of the incident
Table e4: Patient-sided possible risk factors
Table e5: Reactions to the incident
Table e6: Proposals to avoid further incidents

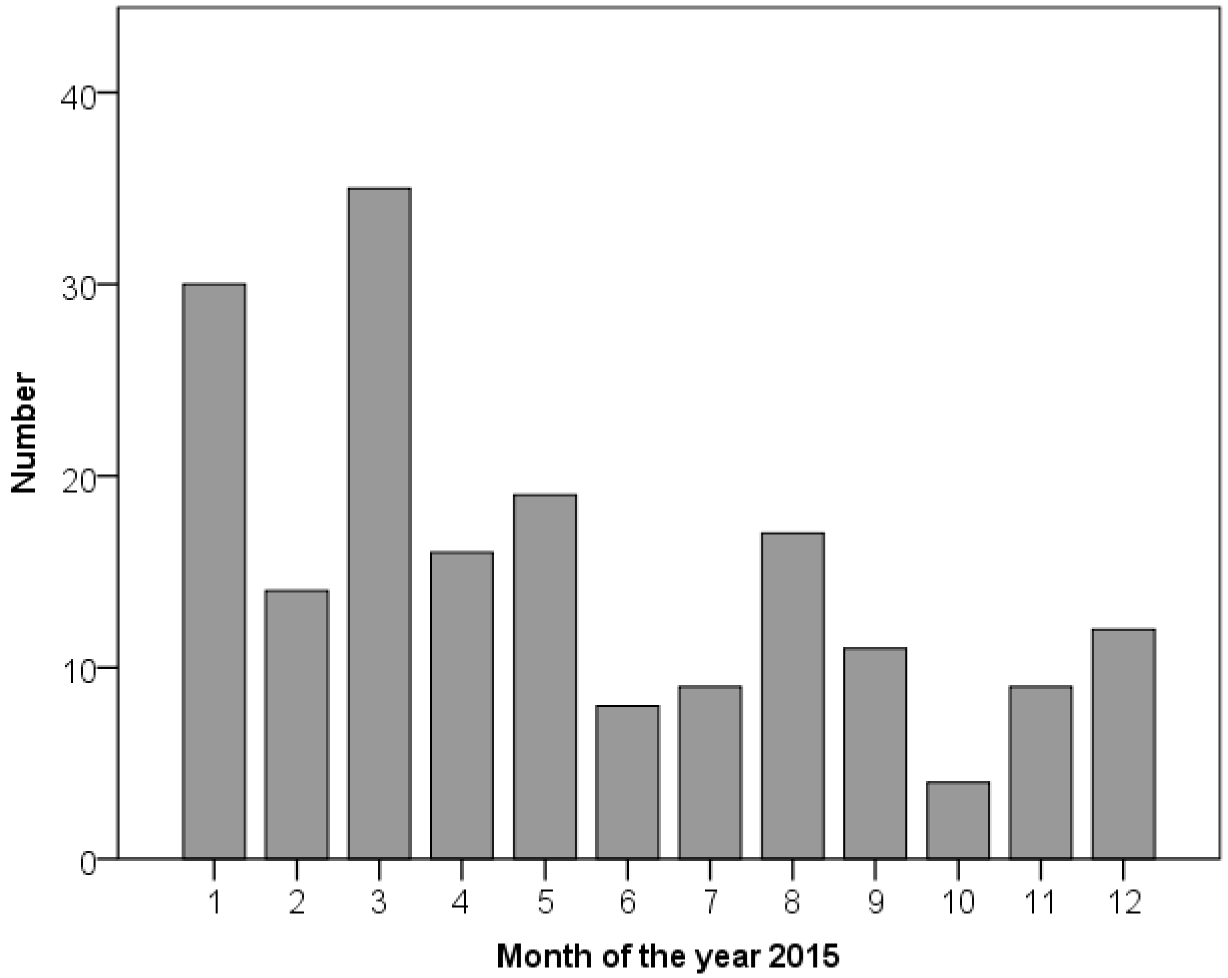

Figure e1. Distribution of the incident notifications over the year. Because only the week has been reported, the assignment to the month is approximate.

Type of error	N*	Percent*, %
Dosing too high	41 (12)	20.9 (6.1)
Dosing too low	21 (6)	10.7 (3.0)
Dosing too short	1 (2)	0.5 (1.0)
Dosing too long	5 (4)	2.6 (2.0)
Wrong way of administration	1 (1)	0.5 (0.5)
Wrong medication applied	56 (12)	28.6 (6.1)
Necessary medication not applied	12 (9)	6.1 (4.6)
Defective or expired medication applied	1 (1)	0.5 (0.5)
Problems with insurance reimbursing	1 (0)	0.5 (0.0)
Other problem**	31 (6)	15.8 (3.0)
Unknown	1 (0)	0.5 (0.0)
Multiple naming	26 (n.a.)	13.3 (n.a.)
Total	197 (53)	100.0 (26.9)

Table e1. What went wrong with the incidents. * Parenthesis denotes the additional naming within the category "multiple naming". ** Naming within the category "other" were: confusion of similar trade names (2), dosing error (6), erroneous package size (1), confusion of similar looking preparations (1), error when controlling blood levels (2), double dosing (4), missed discontinuation of medication (1), necessary treatment not applied (1), wrong vaccine applied (4), missed re-uptake of anticoagulation after operation (1), contra-indication overlooked (2), drug-drug interaction overlooked (3), known intolerance overlooked (3), necessary monitoring missed (2), transgression of maximal drug allowance (2), delivery of incomplete package (1), confusion by contradictory medication lists (3), incorrect handling of administering device (1), uncontrolled taking of "nature medicines" (1), unreliable compliance with medication plan (2), two medications of the same drug

class (3), forbidden bisection of pills (2), confusion of similar named patients (1), intake of medication of the neighbor resident in a home (1), other (9).

Organ system	N*	Percent*, %
Cardiovascular	14 (10)	15.4 (11.0)
Central nervous system	23 (10)	25.3 (11.0)
Gastro-intestinal	5 (3)	5.5 (3.3)
Kidneys	2 (2)	2.2 (2.2)
Liver	2 (0)	2.2 (2.2)
Lung	2 (1)	2.2 (1.1)
Skin	6 (0)	6.6 (0.0)
Other**	24 (6)	26.4 (6.6)
Multiple naming	13 (n.a.)	14.3 (n.a.)
Total	91 (32)	100 (35.2)
Question does not apply	106	/

Table e2. Organ system involved. * Parenthesis denotes the additional naming within the category “multiple naming”. ** Naming within the category “other” were: endocrine system (9), musculo-skeletal (8), ear-nose-throat (2), psychic (1), other (9).

Causes of the incident	N*	Percent*;%
Out of hours	2 (3)	1.0 (1.5)
Communication problems within staff	10 (11)	5.1 (5.6)
Generic substitution of original trade medication by pharmacist	3 (3)	1.5 (1.5)
Difficulties when reading hand-written prescription	1 (1)	0.5 (0.5)
Multiple conflicting prescriptions	5 (8)	2.5 (4.1)
Lacking alertness of physician or practice staff	50 (17)	25.4 (8.6)
Insufficient documentation	3 (11)	1.5 (5.6)
Insufficient patient instruction	7 (17)	3.6 (8.6)
Lacking cooperation of patient / proxies	6 (17)	3.0 (8.6)
Confusion by reading package leaflet	1 (0)	0.5 (0.0)
Confusion after “Googleing”	1 (0)	0.5 (0.0)
Administrative problems	2 (3)	1.0 (1.5)
Defective medication as caused by manufacturer	3 (0)	1.5 (0.0)
Lacking maintenance (e.g. emergency case)	1 (0)	0.5 (0.0)
Lacking use of treatment aids (e.g. Dosett)	0 (6)	0.0 (3.0)
Other source of trouble**	54 (18)	27.2 (9.1)
Unknown	3 (0)	1.5 (0.0)
Multiple naming	45 (n.a.)	23.1 (n.a.)
Total	197 (115)	100.0 (58.4)

Table e3. Causes of the incident. * Parenthesis denotes the additional naming within the category “multiple naming”. ** Naming within the category “other” were: Erroneous delivery in pharmacy (5), treatment delayed/hampered by patient (2), transcription error (2), missed

discontinuation of an ongoing treatment (2), communication problem within helpers' network (16), similar trade names (2), interface problems with hospital (4), reading error of patient / proxies (3), erroneous execution of a medical prescription (14), missed follow-up control (9), difficult handling of a preparation (2), disregard of possible interactions (2), transgression of competence by care workers (5), incomplete information by patient / proxies (8), stress / lack of time (12), other 25.

Item *(number of missing observations in groups "less" / "more")*	Relevance		All n (%)
	less n (%)	more n (%)	
Number of patients	124 (100.0)	73 (100.0)	197 (100.0)
Living situation (1/0)			
- together with mate / family	61 (49.6)	31 (42.5)	92 (46.9)
- alone	28 (22.8)	23 (31.5)	51 (26.0)
- institution	33 (27.6)	19 (26.0)	53 (27.0)
Social problems (2/4)			
- yes	22 (18.1)	18 (26.1)	49 (20.9)
Dementia or mental illness (2/2)			
- yes	31 (25.4)	18 (25.4)	49 (25.4)
Psychiatric problems (1/2)			
- yes	28 (22.8)	28 (38.4)*	56 (28.9)
Treatment with psychotropic drugs (0/0)			
- yes	50 (40.3)	39 (53.4)	89 (44.2)
Linguistic problems (0/1)			
- yes	11 (8.9)	5 (6.8)	16 (8.2)
Smoking (7/4)			
- yes	11 (9.4)	8 (11.6)	19 (10.2)
Substance abuse other than nicotine (2/1)			
- yes	3 (2.5)	5 (6.9)	8 (4.1)
Visual blurring (6/6)			
- yes	4 (3.4)	4 (6.0)	8 (4.3)

Hearing problems (3/5)			
- yes	8 (6.6)	4 (5.9)	12 (6.3)
Gait problems (2/2)			
- yes	40 (32.8)	18 (25.4)	58 (30.1)
Renal insufficiency (GFR<60 ml/min*1.73m^2) (6/2)			
- yes	24 (20.3)	21 (29.6)	45 (23.8)
Liver cirrhosis of other hepatic function problem (3/1)	3 (2.5)	3 (4.2)	6 (3.1)
- yes			

Table e4. Patient-sided possible risk factors. * p=0.021 “more” vs. “less” by chi-square testing. We calculated the variable “incident relevance” from the variables “disturbance” and “endangering” of the patients; if any of them was graded with “medium” or higher, the variable relevance was set to “more”, otherwise to “less”.

Reactions to the incident	N*	Percent*; %
Changing standard operating procedures of the practice	14 (3)	7.2 (1.5)
Better instruction of patients	23 (4)	11.7 (2.0)
Communication with other institutions	31 (3)	15.7 (1.5)
Notifying manufacturer	1 (2)	0.5 (1.0)
Reporting the incident to the critical incident reporting system	10 (6)	5.1 (3.0)
No reaction at all	55 (n.a.)	27.9 (n.a)
Other type of reactions to the incident**	49 (9)	24.9 (4.6)
Missing information	1 (0)	0.5 (0.0)
Multiple naming	13 (n.a.)	6.6 (n.a.)
Total	197 (27)	100.0 (13.7)

Table e5. Reactions to the incident. * Parenthesis denotes the additional naming within the category "multiple naming". ** Naming within the category "other" were: Hire more workforce (1), arrangements with other physicians (2), with pharmacist (4), with community nurse (4), with institution (12), with practice nurse (6), with specialist (1), with patient (3), with supplier (1), sending a new medication plan (1), having regular staff meetings (1), remove Digoxin 0.25 from assortment because of safety reasons (1), to not perform vaccinations in absence of vaccination card (2), to clarify intolerances (2), to clarify interactions (1), to hand out an allergy card (1), to actualize patient records (3), to apply for insurance cost credit (1), to organize follow-up controls (20), other (5).

Proposal	N*	Percent*;%
Cross-check of medication lists	6 (14)	4.8 (11.2)
Patient instructions	2 (3)	1.6 (2.4)
Reduction of time pressure / stress	3 (6)	2.4 (4.8)
To observe adverse drug reactions also with “nature products”	1 (0)	0.8 (0.0)
Four eyes check when dispensing medication	2 (4)	1.6 (3.2)
No medication without prescription	3 (6)	2.4 (4.8)
To critically audit polymedication	3 (2)	2.4 (1.6)
To avoid similarly looking or named medication in drug master	2 (3)	1.6 (2.4)
To demand medication plans to be brought to the consultation	0 (4)	0.0 (3.2)
To provide medication plans routinely	2 (8)	1.6 (6.4)
In-deep checking before delivering “new” medication to the patient	4 (1)	3.2 (0.8)
To provide patients with allergy / intolerance cards	2 (1)	1.6 (0.8)
To let patients themselves write their medication card (and controlling afterwards)	0 (3)	0.0 (2.4)
To instruct patients to come into the practice immediately after hospitalization	3 (0)	2.4 (0.0)
To broach regularly “medication safety” on staff meetings	5 (6)	4.0 (4.8)
To provide information in patients’ native language	0 (2)	0.0 (1.6)
To share important information about patients with practice nurse	0 (2)	0.0 (1.6)
To improve information flow	11 (16)	8.8 (10.4)
To organize follow-up controls	10 (28)	8.0 (22.4)
To wait for laboratory results before prescribing	1 (0)	0.8 (0.0)
Other**	12 (9)	9.6 (7.2)
Multiple answering	53 (n.a.)	42.4 (n.a.)

Total	125 (118)	100.0 (94.4)
None	72 (n.a.)	(n.a.)

Table e6. Proposals to avoid further incidents. * Parenthesis denotes the additional naming within the category “multiple naming”. ** Among naming within the category “other” were: timely involving of community nurse, stopping of self-medication in multimorbid or poly-medicated patients, to clearly labelling desensitization suspensions, to separating adult and pediatric vaccines, to implementing timely new guidelines, to refusing to be in care of a patient with bad compliance, to clearly separating and labelling of medication prepared for different patients, to advice patients for separating short- and long-acting insulins at their home, to providing short-time prescriptions with a clear expiration date, to allowing only adequately educated people to align and deliver medication in homes for the elderly and that delivering may be done by the same person who did the preparing of medication boxes, to avoiding similar looking medication boxes being muddled-up by patients at the refectory room.
