# Supplementary material for: Medication incidents in primary care medicine: a prospective study in the Swiss Sentinel Surveillance Network (Sentinella)
Source: BMJ Open. 2017 Jul 26;7(7):e013658. doi: 10.1136/bmjopen-2016-013658 (PMC5642752; doi:10.1136/bmjopen-2016-013658)
Supplement: Author's manuscript [file bmjopen-2016-013658.draft_revisions.pdf]

Medication Incidents in Primary Care Medicine: a Prospective Study in the Swiss Sentinel Surveillance Network (Sentinella)

Journal:	*BMJ Open*
Manuscript ID	bmjopen-2016-013658
Article Type:	Research
Date Submitted by the Author:	29-Jul-2016

Complete List of Authors:	Gnädinger, Markus; University of Zurich, Institute for General Medicine<br>Conen, Dieter; Swiss Patient Safety,<br>Herzig, Lilli; University of Lausanne, Institute of General Medicine<br>Puhan, Milo; University of Zurich, Institute of Epidemiology, Biostatistics & Prevention<br>Staehelin, Alfred; Sentinel Surveillance Network, Swiss Federal Office of Public Health,<br>Zoller, Marco; Zurich University Hospital, Instiute for General Medicine<br>Ceschi, Alessandro; National Poisons Centre, Tox Info Suisse, Associated Institute of the University of Zurich, Division of Science, Head of ; University Hospital Zurich , Dept. Clinical Pharmacology & Toxicology

**Primary Subject Heading**:	General practice / Family practice
Secondary Subject Heading:	Pharmacology and therapeutics, Paediatrics
Keywords:	CLINICAL PHARMACOLOGY, Health & safety < HEALTH SERVICES ADMINISTRATION & MANAGEMENT, Adverse events < THERAPEUTICS

SCHOLARONE™
Manuscripts

Medication Incidents in Primary Care Medicine

A Prospective Study in the Swiss Sentinel Surveillance Network (*Sentinella*)

[1]Markus Gnädinger (corresponding author, markus.gnaedinger@hin.ch), [2]Dieter Conen, [3,4]Lilli Herzig, [5]Milo Puhan, [1,4]Alfred Staehelin, [1]Marco Zoller, [6]Alessandro Ceschi

[1]Institute of Primary Care, University of Zurich, [2]Patientensicherheit Schweiz, Zurich [3]Policlinique Médicale, University of Lausanne, [4]Sentinel Surveillance Network, Swiss Federal Office of Public Health, Bern (*Sentinella*), [5]Epidemiology, Biostatistics, and Prevention Institute, University of Zurich, [6]Division of Clinical Pharmacology and Toxicology, Department of Internal Medicine, Ente Ospedaliero Cantonale, Lugano, Switzerland and Department of Clinical Pharmacology and Toxicology, University Hospital Zurich, Zurich, Switzerland and National Poisons Centre, Tox Info Suisse, Associated Institute of the University of Zurich, Zurich, Switzerland

(**Correspondence:** Markus Gnädinger, Dr. med. Facharzt für Innere Medizin, Birkenweg 8, 9323 Steinach, 0041 71 446 04 64)

Objectives: To describe the type, frequency, seasonal and regional distribution of medication incidents in primary care in Switzerland and to elucidate possible risk factors for medication incidents.

Design: Prospective surveillance study.

Setting: Swiss primary health care, Swiss Sentinel Surveillance Network.

Participants: Patients with drug treatment who experienced any *erroneous* event related to the medication process and interfering with normal treatment course, as judged by their physician. The 180 physicians in the study were general practitioners or pediatricians participating in the Swiss Federal Sentinel reporting system in 2015.

Outcomes: *Primary:* medication incidents; *secondary:* potential risk factors like age, gender, poly-medication, morbidity, care-dependency, previous hospitalization.

Results: The mean rates of detected medication incidents were 2.07 per general practitioner and year (46.5 per 100,000 contacts) and 0.15 per pediatrician and year (2.8 per 100,000 contacts),

For peer review only

respectively. The following factors were associated with medication incidents (OR, 95% CI): higher age 1.004 per year (1.001;1.006), care-dependency 1.458 (1.025;2.073) for care by community nurse, and 1.802 (1.399;2.323) for care by an institution, chronic conditions 1.052 per condition (1.029;1.075), medications 1.052 per medication (1.030;1.074), as well as Thurgau Morbidity Index for stage 4 1.292 (1.004;1.662), 5 1.420 (1.078;1.868), and 6 1.680 (1.178;2.396), respectively. Most cases were linked to an incorrect dosage for a given patient, while prescription of an erroneous medication was the second most common error.

Conclusions: Medication incidents are common in general medicine whereas they rarely occur in pediatrics. Reasons for medication incidents are diverse but often seem to be linked to communication problems. Older and multimorbid patients are at a particularly high risk for medication incidents.

Trial registration: www.clinicaltrials.gov, NCT0229537

Keywords: Patient safety, pharmaceutical preparations, medication errors.

Strength and limitations

This is the first Swiss prospective and systematic collection of incident data in primary care.
It covers three linguistic regions and two drug distribution systems.
It was conducted by experienced physicians and with high response rates.

There was – as expected – bias from selective and underreporting or non-detection of medication incidents.

Proposed reviewers:

- Prof. Tobias Dreischulte, Population Health Sciences, School of Medicine The Mackenzie Building, Kirsty Semple Way, Dundee DD2 4BF, t.dreischulte@dundee.ac.uk
- Prof. Meredith A B Makeham, MPH(Hons), FRACGP, Lecturer and NHMRC Scholar, Discipline of General Practice, The University of Sydney, Sydney, NSW. makeham@ozemail.com.au

For peer review only

For peer review only - http://bmjopen.bmj.com/site/about/guidelines.xhtml

Introduction

Patient safety is a major concern in healthcare systems worldwide. Although most safety research has been conducted in the in-patient setting [1], evidence indicates that medical errors and adverse events pose a serious threat for patients in the primary care setting as well, since most patients receive ambulatory care [2-4]. The rationale of this project has been published in our study protocol [5]. The *aim* of the project was to describe the type, incidence, seasonal and regional distribution of medication incidents in primary care in Switzerland and to elucidate risk factors for medication incident.

Method

Study design

We conducted a prospective surveillance study among primary care patients during 2015 to identify cases of medication incidents.

Study population

The study population was any person undergoing drug treatment in general internal or pediatric practices participating the *Sentinella* network. The latter covers a representative sample of patients in primary care for Switzerland [6, *included to this manuscript*]. Founded in 1986, it was mainly designed to survey transmissible diseases. Later, it also assessed other health problems of public interest. It generates daily to weekly current data and covers the entire geographic and linguistic regions of our country. Children, the mentally handicapped or the elderly were also included, all of whom might be at increased risk for medication errors.

Medication incidents

We defined medication incidents as any erroneous event (as defined by the physician) related to the medication process and interfering with normal treatment course (e.g. administration of an erroneous medication). We did not include lack of treatment effect, adverse drug reactions or drug-drug or drug-disease interactions without detectable treatment error. Nor did we consider medication incidents if patients refused to have them reported to the *Sentinella* system.

For peer review only - http://bmjopen.bmj.com/site/about/guidelines.xhtml

Data sources

The study physicians recorded the patient's year of birth and gender on their weekly reporting form. After a maximum of four weeks they had to fill in a detailed incident questionnaire (Appendix A). It was comprised of their *Sentinella* number and the calendar week of notification. Concerning the patients, they reported the living situation, several supposed risk factors for an incident, as well as the following variables: hospitalization during the previous year, care-dependency, number of drugs used chronically, number of chronic conditions, and the Thurgau-Morbidity-Index (TMI), to be compared with a denominator analysis (below) [6]. We further received a detailed description of the incident and proposals to avoid future incidents.

We got the annual number of patient-to-physician contacts (PPC) per practice from the *Sentinella* administration, as well as morbidity data from a fortnight cross-sectional denominator analysis of all patients consulting a *Sentinella* practice during weeks 11 or 12 [6].

We received the anonymized list of participating physicians, their specialty, as well as the community size (Swiss Federal Statistical Office) and the linguistic region from the *Sentinella* administration. Information on Swiss medication sales in 2015 by ATC groups was derived from Interpharma Switzerland (Appendix F).

The questionnaires were completed either electronically or on a paper/pencil version, the former as online SurveyMonkey™ questionnaires, the latter as sealed envelopes sent from the *Sentinella* administration. We had three study questionnaires: a detailed incident questionnaire, an initial one, and a final one (Appendices A, B, and C). The initial questionnaire served to describe the physician's practices in terms of e.g. number of physicians, availability of electronic patient history, electronic drug-drug interaction control system, and drug distribution system (Appendix B). The final questionnaire investigated non-reporting and difficulties with coding the morbidity variables (Appendix C).

We calculated the variable "incident relevance" from the items "disturbance" and "endangering" of the patients; if any of them was graded with "medium" or higher, the variable relevance was set to "more", otherwise it was set to "less". Because of question ambiguity, we set coding of the item care-dependency to "missing" for patients younger than 20 years of age. Evans' Index – a prognostic index – was calculated by simple addition of the number of chronic drug treatments with the number of chronic conditions [7].

For peer review only - http://bmjopen.bmj.com/site/about/guidelines.xhtml

The following free text variables were manually coded: relationship of the incident to the suspected medication, preventability of the incident, reactions to the incident, and proposals to avoid further incidents.

Statistical methods

Values are given as frequencies, mean ± SD or median [interquartile range (IQR)], depending on non-normal distribution or non-interval scaled data level. To assess the association of medication incidents with potential risk factors we used the GENLINMIXED procedure. Clustering of patients was addressed by using a mixed binary logistic regression with the fixed factors of gender, year of birth, care-dependency, number of chronic drug treatments, number of chronic conditions, and TMI as well as the physicians' practice number as a random factor; if one item was missing, the whole record was excluded from the analysis. We used IBM SPSS 23.

Results

The Sentinella system

During the year 2015, 149 practices were enrolled to the *Sentinella* system. Of them, 144 practices were known to report regularly; their properties are listed in Table 1. The *Sentinella* physicians are representative for the overall Swiss physician population [5,6]. Drugs were auto-distributed by 42% of the study practices. Approx. half of the physicians had electronic and the other paper-based medical records. Systematic drug-drug interaction control systems were installed only by a minority (36.8%) of the physicians. During the year 2015 (which included unusually for calendar adaptation, 53 instead of 52 reporting weeks), the general practitioners (GPs) had 4,456±2,137 PPC; for the pediatricians (PEDs), these were 5,297±2,715.

Study flow

During the year 2015, we received 216 incident notifications (Figure 1). In 11 cases, we did not receive the detailed notification form. Eight cases had to be removed from the database because they fulfilled the exclusion criterion (i.e. "adverse drug reactions *without* detectable error"), and one case had to be removed because of double data entry. This led to 197 cases which could be analyzed. The distribution of the monthly incident notifications throughout 2015 is depicted in Figure e1 (Appendix D), and the distribution of the numbers of cases reported by each practice in Figure 2.

Description of patients

For peer review only

Table 2 lists age, gender and geographic distribution as well as the physician-to-patient relationship and the observer of the incident. Only three cases evolved in PED practices. No statistically significant differences were found between the two relevance classes of the incidents.

Number of incidents, non-reporting

In the GPs 194 incidents, 148 physicians, 4,456 yearly PPC led to 1.31 incidents per physician and year or 29.4 per 100,000 PPC; in PEDs 3 incidents, 32 physicians, 5,297 yearly PPC led to 0.1 incident per physician and year or 1.8 per 100,000 PPC.

To evaluate the *non-reporting of incidents*, we asked the physicians in the final study questionnaire. Out of 180 actively reporting physicians we received 145 questionnaires (80.6% response rate). To our question: “Did you **not** report medication incidents that you had noticed during the last year?”, they answered: “Never or almost” 110 (75.9%), “yes, but seldom” 22 (15.2%), “yes, frequently” 9 (6.2%), “always, or almost” 4 (2.8%). Reasons for not reporting incidents were “lack of time” or “forgetfulness”. If we speculate that these answers represent reporting rates of 76-100, 51-75, 26-50, or 0-25% respectively, we could divide the observed rates by the middle of the reporting classes: 0.875, 0.625, 0.375 and 0.125. By doing so, we calculated a rate of 50% of underreporting; if we furthermore consider the 5% of the incidents where the questionnaires were not sent, this rate increases to 58%. We therefore have to multiply the observed rates with a factor of 1.58 resulting in the following rates of detected incidents: GP 2.07 per physician and year, 46.5 per 100,000 PPC and PED 0.15 per physician and year, 2.8 per 100,000 PPC.

Types and causes of incidents, organ systems involved, preventability

The *types of error* are listed in Table e1 (Appendix D) and Figure 3; in 26 cases more than one was mentioned. Most cases were linked to an incorrect dosage for a given patient, while prescription of an erroneous medication was the second most common error.

Most errors concerned orally applied medication. *Parenterally* applied drugs led to fewer error reporting; in our study they comprised insulin and vaccinations. There were cases of an incorrect (influenza vs. anti-tetanus) or incomplete (Boostrix® vs. Boostrix-Polio®) vaccination and of undue vaccination (a third anti-HPV vaccine). The first case was due to a communication problem within the practice staff, the other to a vaccination in absence of the vaccination card.

In 89 of the 195 cases, *organ system* damage was reported, in 13 cases more than one organ system was involved, most frequently the central nervous system (Figure 4) (Table e2, Appendix D). Possible *triggers* of the incident were reported in 194 of the 197 cases; in 45 cases there was more than one single reason, most frequently lacking alertness of the reporting physicians or their staff

For peer review only - http://bmjopen.bmj.com/site/about/guidelines.xhtml

(Table e3). When asked who might be the "*responsible*" for the incident, 191 replied. The most common person or institution possibly responsible for the medication incident was: the reporting physician 41 (21.5%), followed by the practice nurse 26 (13.6%), the institution where the patient lives 33 (17.3%), the pharmacy 7 (3.7%), the hospital 12 (6.3%), the community nurse 4 (2.1%), the patients or their proxies 15 (7.9%), and the manufacturer 2 (1.0%); in 9 cases (4.6%) this was unclear, in 37 cases (19.4%), there was more than source one to blame. *Preventability* of the incidents was classified (by our study board): unlikely in 6 cases (3.0%), possible in 58 (29.4%), probable in 114 (57.9%), and definite in 19 (9.6%).

Endangering and disturbances

In 192 of the 197 cases we received information about patients' *endangering* as estimated by the physicians. In 39 cases (20.3%) there was no endangering, in 83 (43.2%) light, in 51 (26.6%) moderate, in 19 (9.9%) severe. The *disturbances* caused by the incident are listed in Table 3. Out of the 197 incidents, 74 (37.5%) were classified as "more" relevant (Table 2).

Interface problems, orientation, predictability, repeat incidents

The presence of *interface problems* was reported in 64 of 197 cases (32.4%); these were: with a hospital in 28 cases (43.8%), with an institution in 14 (21.9%), with a community nurse in 6 (9.4%), with a pharmacist in 9 (14.0%), with a specialist in 3 (4.7%), and with others in 4 (6.3%). In 184 cases, we received information about *orienting* patients about the incident; in 98 cases, the patient was oriented by the reporting physician or his staff (50.3%), non-orientation was stated: because the patient was not able to understand (children, demented) in 26 cases (14.1%), because the problem had already been solved and the notification would have unduly disturbed the confidence in 18 cases (9.8%), because the patient or the proxies had observed themselves in 23 cases (12.5%), because the patient had already been oriented by others in 7 cases (3.8%) or because of another reason in 12 cases (6.5%). As for *predictability* of the incident, we received 183 valid answers; of them 82 (44.8%) stated "yes, in the given constellation, the incident was to be expected". When asked whether they had already reported a *similar incident* in the study, 29 out of 196 (14.8%) answered in the affirmative.

Risk factors to undergo a medication incident

To detect patient *risk factors* to undergo an incident, we compared the incident data with those of a fortnight denominator analysis [6]; the following univariate factors accumulated preferentially in incident patients: higher age, care-dependency, higher numbers of chronic conditions or medications (or higher Evans' Index), as well as higher TMI (table 4). In the multivariate analysis only in-patient care by an institution remained a significant factor. Other suspected risk factors are listed in

For peer review only

Table e4 (Appendix D); these items were not included in the denominator study and therefore lack a comparator. When discerning patients with a more from them with a less relevant incident, only psychiatric illness reached a significantly increased proportion. We did not detect major differences between the two drug distribution systems.

Relationship between incidents and drug class, ATC-group

We assessed semi-quantitatively whether the class of the suspected drug was causally linked to the emergence of the incident; the frequencies were: none 49 (25.1%), unlikely 31 (15.9%), possible 77 (39.5%), probable 34 (17.4%), definite 4 (2.1%), did not apply 2. ATC codes of the suspected medications are listed in Table 5. Most naming concerned the groups C “cardiovascular” (23.2%) and N “nervous system” (22.1%). Among the medication classes judged to be *related* to the incident (n=37) the most frequently named group were oral anticoagulants: rivaroxaban 9, phenprocoumon 7, and acenocoumarol 2 cases; this explains the 7-fold increased relative risk of ATC group B as compared to sales. As an example for errors *without* relation to the drug class we must mention the 17 cases of institutionalized patients ingesting medications scheduled for other residents, in most cases this was person eating at the same table, but in other cases the person in care mixed up patient names.

Reactions to the incident and suggestions to prevent further ones

In 141 of the 197 cases, the physicians reported to have changed something after the incident, in 13 cases this was more than one *reaction*, mostly often named were communication with other caregivers or better instruction of patients (Table e5, Appendix D). The respondents were asked for proposals how to *prevent* future incidents of the reported type; 125 of them made a total of 243 suggestions (Table e6, Appendix D). Of these, 37 (15.2%) were related to accurate medication lists, 10 (4.1%) to better patient instructions, 38 (15.6%) to organization of regular follow-up controls, and 55 (22.6%) involved organizational changes within the practice and its staff.

Discussion

In a representative group of primary care physicians [8], we found approximately one case of a medication incident per GP and year.

Incident rates

We calculated the rates of detected medication incidents as follows: GP 2.07 per physician and year, 46.5 per 100,000 PPC and PED 0.15 per physician and year, 2.8 per 100,000 PPC. Medication incidents may make up a proportion of approximately one third of all incidents [9]; the rates for

For peer review only - http://bmjopen.bmj.com/site/about/guidelines.xhtml

all safety incidents may amount 6.20 per physician and year or 139.4 per 100,000 PPC in GPs and 0.45 per physician and year or 8.4 per 100,000 PPC in PEDs.

In Australian primary care patients an incident rate of 4.98 per GP and year was reported; this is close to our estimated rate of 6.20; hence, there was no sub-typing of the incidents [10]. In a three-year study, O'Beirne and colleagues reported a rate of 1.8 safety incidents per year and physician [9]. In a literature review of western countries' publications, Sandars and Esmail described a rate of 5 to 80 errors per 100,000 consultations [2], a rate somewhat lower than the 139.4 cases per 100,000 consultations estimated in our study. Kuo and colleagues reported a proportion of 15% of all incidents to be related to medication [11]; since we estimated a proportion of 33% this would give rise to even higher rates of all safety incidents when calculated from our study database. The 13-fold higher incident rate in GPs as compared to PEDs is not surprising given the lower medication rates in children. A recent British study confirmed this much lower rate of incidents in children; out of 46,902 family practice safety reports, 1,788 concerned children (26-times less than adults) [12].

Definition of incidents and reliability of reporting

On the other hand, incident rates may be influenced by their definition. Gandhi and colleagues coined the term "avoidable adverse drug reaction" [13]. In our study, we explicitly excluded adverse drug reactions *without* detectable error. Runciman and colleagues defined a patient safety incident as follows: "An event or a circumstance that could have resulted or did result in unnecessary harm to a patient" [14]. An intuitive definition of a medical error was given by Makeham et al: "That was a threat to patient wellbeing and should not happen. I don't want it to happen again." [15]. As shown in Figure e1 (Appendix D), reporting frequency was higher at the beginning of the study as compared to the later course of it. This could reflect some loss of interest or forgetfulness by the reporting physicians. As calculated from our final questionnaire after the study, the reporting physicians failed to report about one in three cases of the detected medication errors. Non-detection of incidents may even be more frequent than non-reporting of observed incidents. A missed possible drug-drug interaction may be detected by chart review, a documentation error would probably have been found only in 1:1 supervision, which is very time-consuming, costly and may additionally influence performance of the observed physician. It is therefore virtually impossible to decipher the real rate of non-detected incidents. The problems in detection of incidents were the reason to postulate a "mix of methods" as needed to identify adverse events in general practice [16].

Other approaches

In a retrospective, semi-quantitative analysis, Gehring and colleagues investigated safety incidents in Swiss primary care [17]; among 23 predefined classes of safety incidents, the respondents ad-

For peer review only - http://bmjopen.bmj.com/site/about/guidelines.xhtml

mitted 15 to have occurred at least yearly – four of them being linked to drug treatment. Another approach to incidents is to ask patients as performed by Mira and colleagues [18]; they interviewed patients (> 65 years with five or more chronic drug treatments) and found that 75% of the patients reported to have been affected by at least one medication error during the previous twelve months. We did not collect data on appropriateness of treatment; hence medication inappropriate for some groups of patients (elderly or patients with impaired renal function) may provoke incidents [19]. A recent Scottish study demonstrated an impressive reduction in high-risk prescriptions (nonsteroidal anti-inflammatory drugs, antiplatelet, and anticoagulation) as well as hospital admissions for gastrointestinal ulcers or heart failure by a combination of educational measures, informatics and financial incentives [20].

Type, consequences, causes and preventability of error

Most cases involved application of an erroneous dosing or of a wrong medication; non-application of necessary medication was also frequent. The prototype of wrong medication was confounding of prepared medication in home residents. The classic case of non-application of a necessary medication was the missed re-uptake of anticoagulation after an operation. Non-application of necessary drugs seems to be a relevant source of unnecessary harm to patients [21]. The repartition of the incidents was similar as reported by others [11,17,18,22]. More than half of the patients did not have any disturbances after the incident; otherwise in most cases the nervous system was affected. No fatalities were reported, but seven patients (3.5%) needed stationary care. In 2004 Pirohamed and colleagues published a study on adverse drug reactions as a cause for admission to hospital; they found that 6.5% of all hospitalizations and 4.0% of all hospital stays were caused by adverse drug reactions, 72% of them preventable and 2% leading to death [23], hence, this study included all cases, and therefore a majority of cases of adverse drug reactions *without* a detectable error. An older Swiss study was published by Livio and colleagues; out of 3,195 hospitalizations, she identified 229 cases (7.2%) as probably caused by adverse drug reactions [24]. In that study, 32% of the events were classified as being preventable (which sensu strictu does not mean that an error had caused the incident), and in 6% of the cases, fatalities were reported. Concerning circumstances, most naming was – as found in our patients – lacking alertness and communication problems within practice staff, but there was a large variety of other topics. In inpatients, one in ten drug administrations was described to be erroneous [25]. Medical errors seem to be the “third leading cause of death in the US” [26].

Risk factors

When looking for propensity factors, the univariate analysis comparing a fortnight denominator with

For peer review only - http://bmjopen.bmj.com/site/about/guidelines.xhtml

our data revealed all factors investigated (except gender) as significantly accumulated in the incident group; these were higher age, care-dependency, higher numbers of chronic conditions or medications (and higher Evans' Index), as well as higher TMI. When performing a multivariate analysis, only those living in a home for the elderly or handicapped remained a significant risk factor, but the small number of observations precluded possible less important risk factors to be detected. The only significant risk factor for undergoing an incident of higher *relevance* was psychiatric disease. In the literature there are several factors described to correlate with proneness to undergo a medication incident, which quite well reflects the results of our study. Mainly, this is evidently the number of drugs ingested [13,27]. Higher or young age [28], or morbidity [29,30] were also described. Most incidents concerned ATC-groups N "nervous system" or C "cardiovascular", which were also of the mostly sold drugs; an exception was group B with anticoagulants and a sevenfold increased relative risk as compared to Swiss sales in 2015. It seems wise to be alert to avoid errors when prescribing medication of these groups. The prevailing position of anticoagulants (18.5% of cases) was described also by Field and colleagues [30]. Otherwise the repartition of our suspected drugs was similar to other primary care studies [11,30], a literature review [31] or a theoretical paper [22].

Prospects

First, incidents were endured like stormy weather, and worse culprits were blamed. Thereafter – inspired by flight companies - critical incident analysis (safety I) was adopted in medicine, preferentially in anesthesia. In anonymous systems, incidents were analyzed and ideas come up with to avoid further emergence of similar incidents. Perhaps this is now the moment to think about *safety II*, meaning that systems should be organized in a resilient way insensitive to perturbations and tolerant to time pressure, misunderstandings and mistakes [32,33].

Conclusion

Medication incidents are common in general medicine whereas they rarely occur in pediatrics. Reasons for medication incidents are diverse but often seem to be linked to communication problems. Older and multimorbid patients are at a particularly high risk for medication incidents.

Abbreviations

GP general practitioner

PED pediatrician

For peer review only - http://bmjopen.bmj.com/site/about/guidelines.xhtml

PPC patient-to-physician contacts

TMI Thurgau Morbidity Index

Author affiliations

MG Institute of Primary Care, University of Zurich, Switzerland.

AC Division of Clinical Pharmacology and Toxicology, Department of Internal Medicine, Ente Ospedaliero Cantonale, Lugano, and Department of Clinical Pharmacology and Toxicology, University Hospital Zurich, Zurich, and National Poisons Centre, Tox Info Suisse, Associated Institute of the University of Zurich, Zurich, Switzerland

DC Patientensicherheit Schweiz, Zurich, Switzerland.

LH Policlinique Médicale, University of Lausanne, Switzerland and Sentinel Surveillance Network, Swiss Federal Office of Public Health, Bern, Switzerland.

MP Epidemiology, Biostatistics, and Prevention Institute, University of Zurich, Switzerland.

AS Sentinel Surveillance Network, Swiss Federal Office of Public Health, Bern, Switzerland.

MZ Institute of General Medicine, University of Zurich, Switzerland.

Acknowledgements

We are grateful to Lee Wennerberg for the English language corrections, Dr Sven Staender, Männedorf for the helpful comments, and Simon Gnädinger, Zurich for the manual coding of free text items. We thank the *Sentinella* program commission for their support, the reporting physicians of *Sentinella* for their unflagging enthusiastic collection of data, and the Federal Office of Public Health for providing data and translating the questionnaires into French.

Contributorship statement

MG lead the study, did the pilot study (questionnaire development, data entering and processing) wrote all documents, did all the contacts with the *Sentinella* administration, ethics committee, and others, programmed the electronic questionnaires, entered hand-written questionnaires into the database, did the data processing and wrote the publication after data collection.

AC is an expert on clinical pharmacology and drug safety.

DC is an expert on patient safety.

LH is French-speaking and helped to interpret the French questionnaires. She is an expert on multimorbidity. She is a member of the *Sentinella* program commission.

For peer review only

MP is head of Epidemiology, Biostatistics & Prevention Institute. He is responsible for the sound methodology.
AS had the idea for the study. He is vice president of the *Sentinella* program commission.
MZ is expert on electronic data exchange in primary care.
All of them have seen all the study documents and have contributed intellectually to their elaboration. All have contributed to revise the draft of this publication and approve the submitted version of this publication.

Funding

The study was funded by Bangerter-Rhyner Foundation, Basel.

Competing interests

The authors declare that they have no financial interest conflicts with this study.

Patient consent

Not necessary for anonymous data.

Ethics approval

The ethical committee of Canton Zurich decided that our study did not need formal approval because the data are completely anonymous (KEK-ZH 2014-0400). The study was recorded in www.ClinicalTrials.gov: NCT02295371, as well as in our national study registry (www.kofam.ch; SNCTP000001207).

Data sharing statement

No additional data are available.

Strobe statement

Whenever possible, the guidelines of the Strobe statement were followed (Appendix E).

References

1. **Manias E:** Detection of medication-related problems in hospital practice: a review. *Br J Clin Pharmaco*l 2012; 76 (1): 7-20.

For peer review only

2. **Sandars J, Esmail A:** The frequency and nature of medical error in primary care: understanding the diversity across studies. Fam Pract 20 (3): 231-6, 2003.
3. **Miller GC, Britth HC, Valenti L.** Adverse drug events in general practice patients in Australia. *Med J Aust.* 2006;184(7):321-324.
4. **Thomsen LA, Winterstein AG, Søndergaard B, Haugbølle LS, Melander A.** Systematic review of the incidence and characteristics of preventable adverse drug events in ambulatory care. *Ann Pharmacother.* 2007; 41(9):1411-1426.
5. **Gnädinger M, Ceschi A, Conen D, et al:** Medication incidents in primary care medicine: protocol of a study by the Swiss Federal Sentinel Reporting System. BMJ Open 04/2015; 5(4): e007773. DOI:10.1136/bmjopen-2015-007773.
6. **Gnädinger M, Herzig L, Ceschi A et al:** The Burden Related to Chronic Conditions and Multimorbidity in the Swiss Primary Care Population: A Prospective Study in the Swiss Sentinel Surveillance Network (*Sentinella*), 2016, to be submitted. **That manuscript is included in the present submission.**
7. **Evans DC, Cook CH, Christy JM et al:** Comorbidtiy-polypharmacy scoring facilitates outcome prediction in older trauma patients. J Am Geriatr Soc 60: 1465-70, 2012.
8. **Cohidon C, Cornuz C, Senn N:** Primary care in Switzerland: evolution of physicians‘ profile and activities in twenty years (1993-2012). BMC Fam Pract 16: 107, 2015.
9. **O'Beirne M, Sterling PD, Zwicker K et al:** Safety incidents in family medicine. BMJ Qual Saf 20: 1005-10, 2011.
10. **Makeham MAB, Kidd MR, Saltman DC et al:** The threats to Australian patient safety (TAPS) study: incidents of reported errors in general practice. Med J Aus 185: 95-98, 2006.
11. **Kuo GM, Phillips RL Graham D et al:** Medication errors reported by US family physicians and their office staff. Qual Saf Health Care 17: 286-90, 2008.
12. **Rees P, Edwards A, Panesar S et al:** Safety incidents in the primary care office setting. Pediatrics 135 (6): 1027-35, 2015.
13. **Gandhi TK, Weingart SN, Borus BA, et al:** Adverse drug events in ambulatory care. N Engl J Med 348: 1556-64, 2003.
14. **Runciman W, Hibbert P, Thomoson R et al:** Towards an international classification ofr patient safety: key concepts and terms. Int J Qual Health Care 21 (1): 18-26, 2009.
15. **Makeham MAB, Dovey SM, County M et al:** An international taxonomy for errors in general practice: a pilot study. Med J Aus 177:68-72; 2002.

For peer review only - http://bmjopen.bmj.com/site/about/guidelines.xhtml

16. **Wetzels R, Wolters R, van Weel C et al:** Mix of methods is needed to identify adverse events in general practice: a prospective observational study. BMC Fam Pract 2008; 9:35.

17. **Gehring K, Schwappach DLB, Battaglia M et al:** Frequency of and harm associated with primary care safety incidents. Am J Managed Care 18 (9): e323-7, 2012.

18. **Mira JJ, Orozco-Beltran D, Perez-Jover V et al:** Physician patient communication failure facilitates medication errors in older polymedicated patients with multiple comorbidities. Fam Pract 30: 56-63, 2013.

19. **Cooper JA, Moriarty F, Ryan C et al:** Potentially inappropriate prescribing in two populations with differing socio-economic profiles: a cross-sectional database study using the PROMPT criteria. Eur J Clin Pharmacol 72: 583-91, 2016.

20. **Dreischulte T, Donnan P, Grant A et al:** Safer prescribing – a trial of education, informatics, and financial incentives. New Engl J Med 374: 1053-64, 2016.

21. **O'Grady I, Gerrett D:** Minimising harm from missed drug doses. Nursing Times 111 (44): 12-15, 2015.

22. **Huges RG, Ortiz E:** Medication errors. Why they happen, and how they can be prevented. Am J Nursing 205 (3): 14-24, 2005.

23. **Pirmohamed M, James S, Meakin S, et al:** Adverse drug reactions as cause of admission to hospital: prospective analysis of 18 820 patients. BMJ 2004; 329:15–19.

24. **Livio F, Buclin T, Yersin B et al:** Hospitalisations pour effet indésirable médicamenteux. Recensement prospectif dans un service d'urgences médicales [French]. Raisons de Santé 23, 1998.

25. **Berdot S, Gillaizeau F, Caruba T et al:** Drug administration errors in hospital inpatients: a systematic review. PLoS ONE 8 (6): e68856, 2013; doi: 10.1371/journal.pone.0068856.

26. **Makary M, Daniel M :** Medical error – the third leading cause of detha in the US. BMJ 2016 ; 353: i2139.

27. **Nobili A, Pasina L, Tettamanti M et al:** Potentially severe drug interactions in elderly outpatients: results of an observational study of an administrative prescription database. J Clin Pharm Ther 34: 377-86, 2009.

28. **Avery AJ, Ghaleb M, Barber N, et al:** The prevalence and nature of prescribing and monitoring errors in English general practice: a retrospective case note review. Br. J Gen Pract e543, 2013.

For peer review only

For peer review only - http://bmjopen.bmj.com/site/about/guidelines.xhtml

29. **Field TS, Gurwitz JH, Avorn J et al:** Risk factors for adverse drug events among nursing home residents. Arch Intern Med 161: 1629-34, 2001.

30. **Field TS, Mazor KM, Briesacher B, et al:** Adverse drug events resulting from patient errors in older adults. J Am Geriatr Soc 55: 271-6, 2007.

31. **Saedder EA, Brock B, Nielsen LP et al:** Identifying high-risk medication: a systematic literature review. Eur J Clin Pharmacol 70: 637-45, 2014.

32. **Staender S, Kaufmann M.** Sicherheitsmanagement 2015: von “Safety I” zu “Safety II”. Schweiz Ärztezeitung 96 (5): 154-7, 2015.

33. **Gandhi TK, Lee TH:** Patient safety beyond the hospital. New Engl J Med 363 (11): 1001-3, 2010.

For peer review only

For peer review only - http://bmjopen.bmj.com/site/about/guidelines.xhtml

List of figures and tables

Figure 1: Study flow chart

Figure 2: Distribution of the number of cases reported by practice

Figure 3: Type of error

Figure 4: Organ system involved

Table 1: Characteristics of the reporting physicians in 2015

Table 2: General description of the cases

Table 3: Disturbances after the incident

Table 4: Possible risk factors for incident as compared to a denominator analysis during calendar weeks 11 and 12 [Gnädinger M 2016]

Table 5: ACT-Groups of suspected medications

For peer review only - http://bmjopen.bmj.com/site/about/guidelines.xhtml

For peer review only

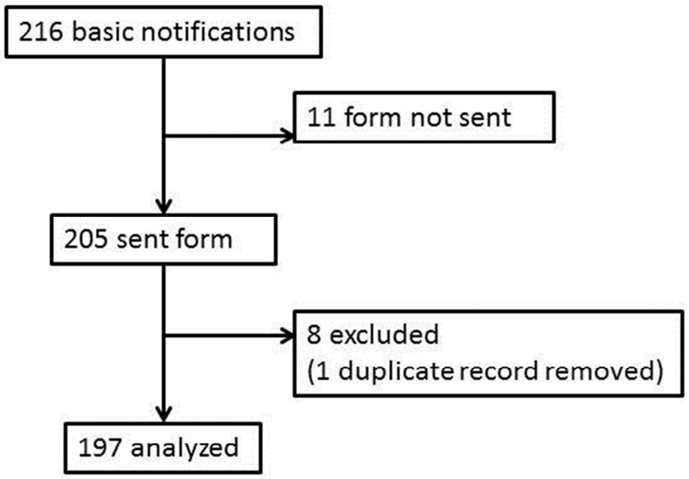

Figure 1. Study flow chart

For peer review only

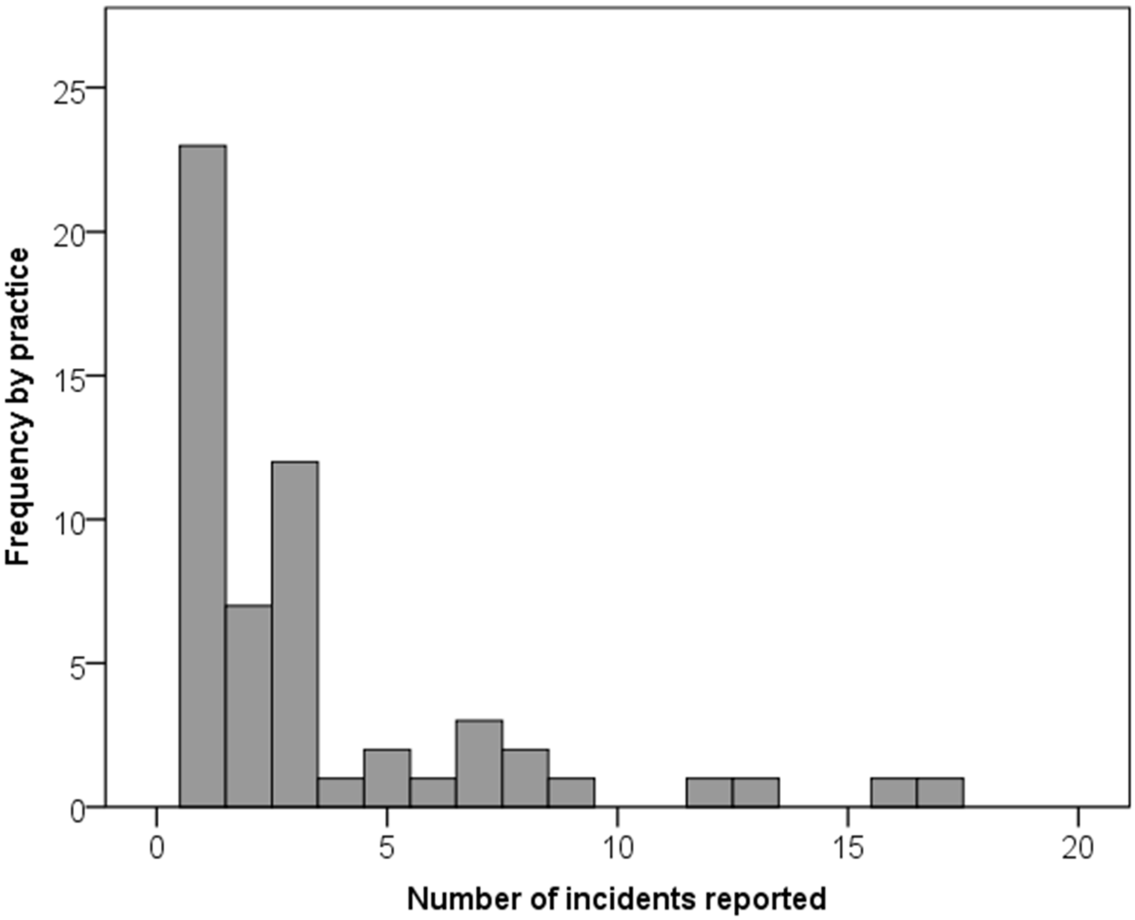

Figure 2. Distribution of the number of cases reported by practice

For peer review only - http://bmjopen.bmj.com/site/about/guidelines.xhtml

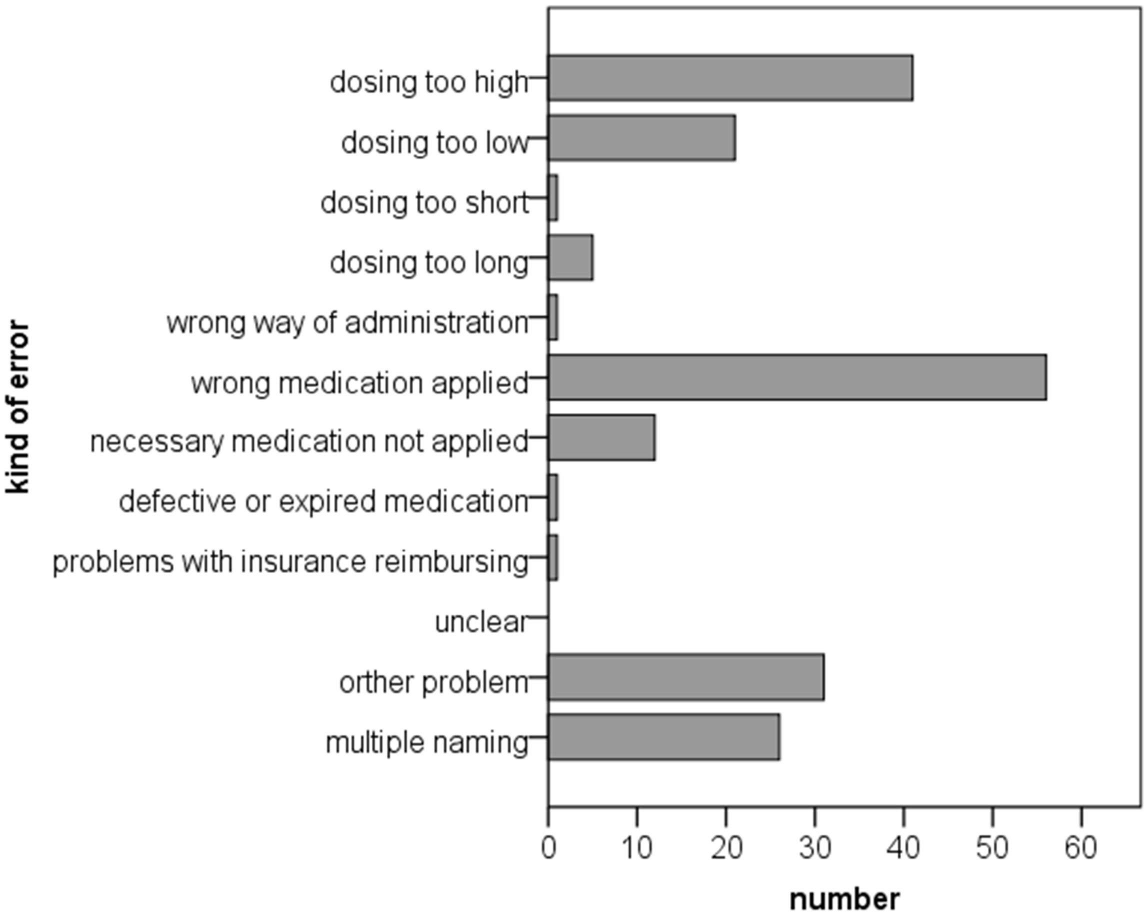

Figure 3. Type of error (n=197 questionnaires). For more detailed information, see Table e1 (Appendix D).

For peer review only - http://bmjopen.bmj.com/site/about/guidelines.xhtml

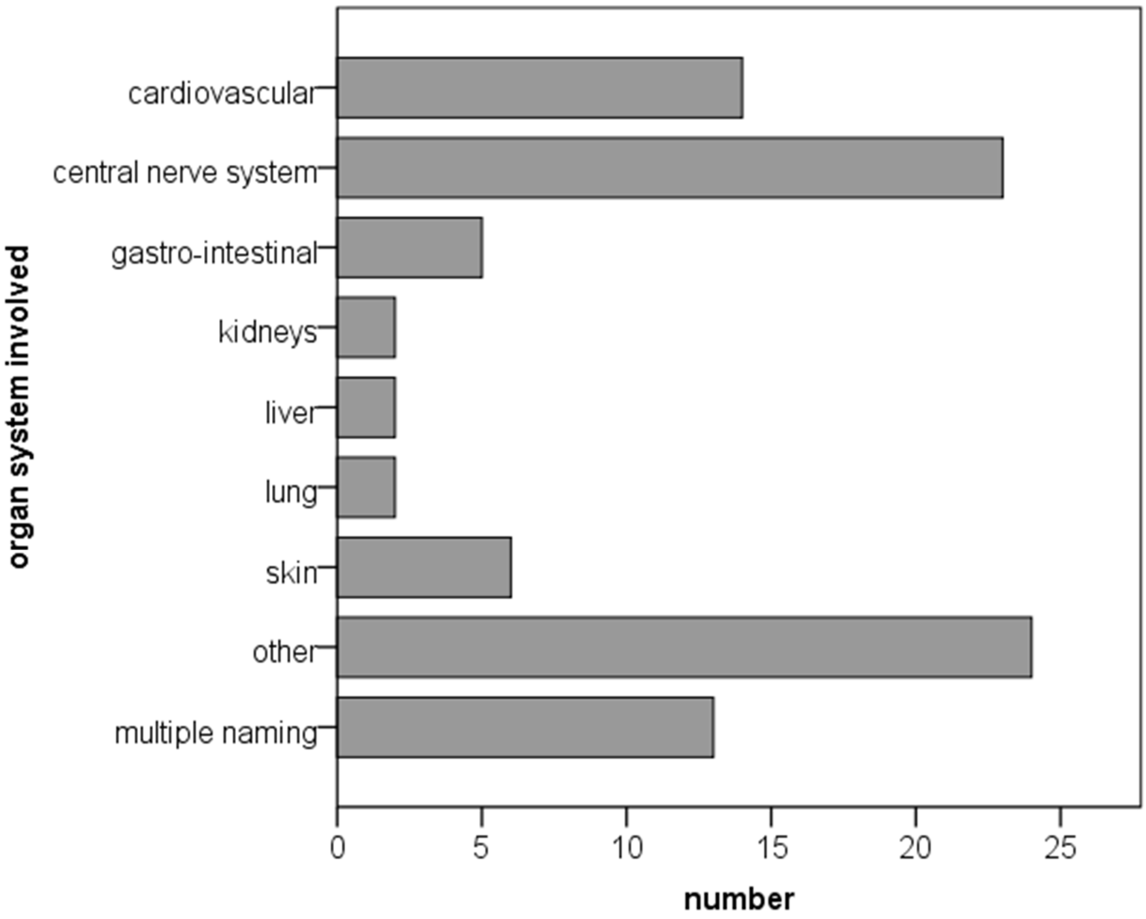

Figure 4. Organ system involved (n=91 of 197 questionnaires). For more detailed information, see Table e2 (Appendix D).

For peer review only - http://bmjopen.bmj.com/site/about/guidelines.xhtml

Physicians' gender	
- male	128 (71.1%)
- female	52 (28.9%)
Physicians' age class	
- < 40	12 (6.7%)
- 40-49	44 (24.4%)
- 50-59	66 (36.7%)
- 60 and over	58 (32.2%)
Specialty	
- GP	148 (82.2%)
- PED	32 (17.8%)
Number of physicians in practice (n=144)	
- 1	69 (47.8%)
- 2	39 (27.1%)
- 3	17 (11.8%)
- 4 to 5	8 (5.6%)
- 6 to 9	7 (4.9%)
- 10 and over	4 (2.8%)
Number of physicians per practice reporting to *Sentinella* (n=144)	
- 1	119 (82.6%)
- 2	19 (13.2%)
- 3	5 (3.5%)
- 8	1 (0.7%)
Linguistic region	
- German	122 (67.8%)
- French	44 (24.4%)
- Italian	14 (7.8%)
Urbanity of the practice	
- urban	93 (51.7%)
- agglomeration	60 (33.3%)
- rural	27 (15.0%)
Workload per week	
- < 15 h	9 (5.0%)
- 15-30 h	36 (20.0%)
- > 30 h	135 (75.0%)
Drug distribution system	
- dispensing by physician	73 (42.2%)
- mixed system	19 (10.6%)
- dispensing by pharmacy	85 (47.2%)
Electronic documentation	
- yes	89 (49.4%)
- no	91 (50.6%)
Electronic interaction control	
- yes	65 (36.1%)
- no	115 (63.9%)
Electronic prescription	
- yes, with thesaurus	62 (34.4%)
- yes, but without thesaurus	24 (13.3%)
- none	94 (52.2%)
Certification of the practice	
- yes	46 (25.6%)
- none	134 (74.4%)
Staff meetings	
- yes, at least monthly	69 (38.3%)
- yes, but less frequently	70 (38.9%)
- none	41 (22.8%)
Quality circle participation	
- yes, at least monthly	134 (74.4%)

For peer review only

-	yes, but less frequently	23 (12.8%)
-	none	23 (12.8%)

Table 1. Characteristics of the reporting physicians in 2015 (144 practices, 180 physicians)
.

For peer review only

For peer review only - http://bmjopen.bmj.com/site/about/guidelines.xhtml

	Relevance		All
	less	more	
Number of cases	124	73	197
Patients age	69.2 ± 20.6	69.4 ± 21.2	69.3 ± 20.8
Patients gender,			
% males	40.3	32.9	37.6
Physicians specialty			
% pediatricians	1.6	1.4	1.5
Linguistic region, %			
- German	75.0	68.5	72.6
- French	18.5	28.8	22.3
- Italian	6.5	2.7	5.1
Physician-to-patient relationship, %			
- own family physician	86.3	78.1	83.2
- urgency / holiday replacing	0.8	4.1	2.0
- institution physician	11.3	17.8	13.7
- other	1.6	0.0	1.0
Observer of the incident; %			
- physician / practice staff	50.0	50.7	50.3
- patient / proxies	21.8	23.3	22.3
- community nurse	1.6	4.1	2.5
- institution (where patient lives)	15.3	16.4	15.7
- hospital	0.8	1.4	1.0
- other physicians	2.4	0.0	1.5
- pharmacist	7.3	4.1	6.1
- other	0.8	0.0	0.5

Table 2. General description of the cases. Differences between “less” and “more” categories are not statistically significant. We calculated the variable “incident relevance” from the variables “dis-

For peer review only - http://bmjopen.bmj.com/site/about/guidelines.xhtml

turbance” and “endangering” of the patients; if any of them was graded with “medium” or higher, the variable relevance was set to “more”, otherwise to “less”.

For peer review only

For peer review only - http://bmjopen.bmj.com/site/about/guidelines.xhtml

Item	N	percent
Severity of disturbance		
- no symptoms but pathological laboratory tests	15	8.0
- light	44	23.4
- moderate	22	11.7
- severe	10	5.3
- fatality	0	0.0
Subtotal (this is the base of the next rows)	*91*	*48.4*
- no symptoms, normal (or no) laboratory tests	97	51.6
Total	188	100.0
Missing data	9	n.a.
All patients	197	n.a.
Time until recovery		
- hours	26	28.5
- days	41	45.1
- weeks	15	16.5
- not yet known or missing information	9	9.9
All patients with disturbances	91	100.0
Recovering		
- without sequels	78	85.8
- with light to moderate sequels	2	2.2
- with severe sequels or fatality*	5	5.4
- not yet known or missing information	6	6.6
All patients with disturbances	91	100.0
Treatment / surveillance		
- not needed	48	52.7
- ambulatory care	33	36.3
- hospital care**	7	7.7
- missing information	3	3.3
All patients with disturbances	91	100.0

For peer review only

Table 3. Disturbances after the incident. * In one case, there was a reduced kidney function, the other cases remained unclear, and no fatalities were reported. ** Two cases had to be surveilled in the emergency room, the hospital stays were: intoxications with thiethylperazine, with fenoterol plus ipratropium bromide, with zolpidem, further a derailed diabetes type 2 (after missed treatment with metformin), and a gastro-intestinal hemorrhage in a patient where antithrombotic treatment with rivaroxaban was not communicated to the physician.

For peer review only

For peer review only - http://bmjopen.bmj.com/site/about/guidelines.xhtml

Item	Patient group		OR (95% CI) (crude)	OR (95% CI) (adjusted)
	Denominator	Incidents		
Number of observations	26,852	197	/	/
Age (mean±SD), years	46.7 ± 27.5	69.3 ± 20.8	1.004 (1.001;1.006)**[a]	1.001 (0.996;1.005)
Gender,				
• male	47.0	37.6	1	1
• female	53.0	62.4	1.048 (0.916;1.197)	1.055 (0.878;1.196)
Care-dependency, number (%)* [d]				
• none	16,335 (85.5%)	96 (51.6%)	1	1
• yes, by proxies	954 (5.0%)	12 (6.5%)	1.121 (0.789;1.594)	0.979 (0.674;1.423)
• yes, by community nurse	723 (3.8%)	22 (11.8%)	1.458 (1.025;2.073)[a]	1.201 (0.821;1.758)
• yes, by institution	1,099 (5.8%)	56 (30.1%)	1.802 (1.399;2.323)[c]	1.528 (1.141;2.046) [a]
Number of conditions (median, IQR)	2 (0;4)	5 (3;7)	1.052 (1.029;1.075)**[c]	1.030 (0.994;1.067)**
Number of chronic active treatments (median, IQR)	1 (0;4)	6 (3;9)	1.052 (1.030;1.074)**[c]	1.030 (0.995;1.067)**
Evans' Index (median, IQR)	3 (0;8)	11 (6;17)	1.009 (1.005;1.013)** [c]	n.a.***
Thurgau Morbidity Index value (%)[d]			[d]	
• 0	8,463 (31.5%)	24 (12.2%)	1	1
• 1	3,611 (13.4%)	8 (4.1%)	0.989 (0.787;1.242)	0.908 (0.694;1.190)
• 2	4,102 (15.3%)	23 (11.7%)	1.049 (0.847;1.300)	0.898 (0.685;1.169)
• 3	3,877 (14.4%)	39 (19.8%)	1.131 (0.914;1.399)	0.830 (0.611;1.127)
• 4	2,119 (7.9%)	39 (19.8%)	1.292 (1.004;1.662)[a]	0.901 (0.643;1.265)
• 5	1,539 (5.7%)	38 (19.3%)	1.420 (1.078;1.868)[b]	0.823 (0.547;1.239)
• 6	709 (2.6%)	26 (13.2%)	1.680 (1.178;2.396)[c]	0.866 (0.523;1.436)
missing values	18	0		

For peer review only

Table 4. Possible risk factors for incident as compared to a denominator analysis during calendar weeks 11 and 12 [Gnädinger M 2016]. * Because of question ambiguity this analysis was restricted to adult patients (age > 19 years); this led to 19,812 valid observations in the denominator and 183 in the incident groups. ** Per one conditions, medication, year or index point. *** Because Evans' Index is a composite of condition and medication numbers; it was not included in the multiple regression analysis. Significance levels: [a] $p<0.05$, [b] $p<0.01$, [c] $p<0.001$. [d] Because GENLIN procedure was not able to process ordinal scaled variables, correlations between study group and TMI or care-dependency were tested with Spearman's rho: the correlation coefficients were +0.075 or +0.094, respectively, $p<0.001$.

For peer review only

ATC-Class		All incidents	Incidents with *probable* or *definite* relationship with medication	Relative Risk (percentages left by right column)	Swiss 2015 sales* (number of packages)
A	Alimentary tract and metabolism	28 (14.6%)	6 (15.8%)	1.06	31,455,252 (14.9%)
B	Blood and blood forming organs	23 (12.0%)	7 (18.4%)	7.08	5,507,624 (2.6%)
C	Cardiovascular system	44 (22.9%)	1 (2.6%)	0.35	16,027,143 (7.5%)
D	Dermatologics	1 (0.5%)	0 (0.0%)	0.00	17,314,810 (8.2%)
G	Genito-urinary system and sex hormones	1 (0.5%)	0 (0.0%)	0.00	7,936,641 (3.7%)
H	Systemic hormonal preparations (excluding sex hormones and insulins)	7 (3.6%)	1 (2.6%)	2.00	2,875,760 (1.3%)
J	Anti-infectives for systemic use	23 (12.0%)	6 (15.8%)	3.95	8,444,623 (4.0%)
K	Infusion liquids	0 (0.0%)	0 (0.0%)	0.00	24,158,749 (11.5%)
L	Anti-neoplastic and immunomodulating agents	5 (2.6%)	1 (2.6%)	2.89	1,934,950 (0.9%)
M	Musculo-skeletal system	4 (2.1%)	2 (5.3%)	0.76	14,787,413 (7.0%)
N	Nervous system	43 (22.4%)	10 (26.3%)	1.30	42,690,195 (20.2%)
P	Anti-parasitic products, insecticides and repellents	1 (0.5%)	0 (0.0%)	0.00	462,559 (0.2%)
R	Respiratory system	7 (3.6%)	3 (7.9%)	0.57	28,837,468 (13.7%)
S	Sensory organs	2 (1.0%)	0 (0.0%)	0.00	7,658,311 (3.6%)
T	Diagnostic use	0 (0.0%)	0 (0.0%)	0.00	43,184 (0.0%)
V	Various	3 (1.6%)	1 (2.6%)	6.50	855,707 (0.4%)
	Total	192 (100.0%)	38 (100%)	1.0	210,990,389 (100.0%)

For peer review only

For peer review only - http://bmjopen.bmj.com/site/about/guidelines.xhtml

does not apply	5	0	n.a.	n.a.

Table 5. ACT-Groups of suspected medications. Relative risk of drugs with probable or definite relationship with the incident as compared to sales proportions. * Information by Interpharma Switzerland (Appendix F).

For peer review only

For peer review only - http://bmjopen.bmj.com/site/about/guidelines.xhtml

Appendices

A) Incident questionnaire
B) Initial physicians' questionnaire
C) Final physicians' questionnaire
D) Electronic figures and tables
E) STROBE statement
F) Swiss drug sales 2015 by ATC groups

For peer review only

For peer review only - http://bmjopen.bmj.com/site/about/guidelines.xhtml

Appendix A Incident questionnaire

Medication Incidents in Primary Care (MIPC)
(Version 1.0, January 25th 2015) (Internet version not available in English)

Incident reporting form

Administrative Information

1. Sentinella identification number: ☐☐☐☐ **2. Week of reporting:** ☐☐

The Patient

3. Year of birth: ☐☐☐☐ **4. Gender:** m ☐ f ☐

5. What was your relationship to the patient when the incident happened? Were you the

family physician ☐ emergency / substitute physician ☐ institution physician ☐ other ☐
if other what kind……………………….

6. What is the patient's living situation?

with partner / family ☐ alone ☐ institution ☐ unknown ☐

7. Are there social problems?
yes ☐ no ☐ unknown ☐

For peer review only - http://bmjopen.bmj.com/site/about/guidelines.xhtml

8. Is the patient demented or otherwise mentally handicapped?

yes ☐ no ☐ unknown ☐

9. Does he suffer from psychological problems?

yes ☐ no ☐ unknown ☐

10. Does he take psychotropic drugs (esp. antidepressants, neuroleptics, benzodiazepines, opiates)?

yes ☐ no ☐ unknown ☐

11. Are there linguistic problems?

yes ☐ no ☐ unknown ☐

12. Does he or she smoke?

yes ☐ no ☐ unknown ☐

13. Is there substance abuse (other than nicotine)?

yes ☐ no ☐ unknown ☐, if yes, what substance? ……………………..

14. Does the patient have uncorrected / uncorrectable visual impairment?

yes ☐ no ☐ unknown ☐

15. Does the patient have uncorrected / uncorrectable hearing impairment?

yes ☐ no ☐ unknown ☐

For peer review only

16. Does the patient have uncorrected / uncorrectable mobility impairment?

yes ☐ no ☐ unknown ☐

17. Is there renal insufficiency (GFR: <60 ml/min/1.73 m^2)?

yes ☐ no ☐ unknown ☐

18. Is there hepatic insufficiency or liver cirrhosis?

yes ☐ no ☐ unknown ☐

19. Was the patient hospitalized in the past 12 months?

yes ☐ no ☐ unknown ☐

20. Is the patient taken care of by others? (*only one answer permitted*)?

yes, family / proxies ☐ yes, community nurse ☐ yes, institution ☐ no ☐ unknown ☐

21. Number of regularly applied active substances (including non-daily applied ones, see guidelines)?

☐☐ unknown ☐

22. Number of chronic conditions (see guidelines)?

☐☐ unknown ☐

23. Scale value of „Thurgau Morbidity Index" (chronic part, see guidelines)?

☐ unknown ☐

For peer review only

For peer review only - http://bmjopen.bmj.com/site/about/guidelines.xhtml

Details of the incident

24. Please, give a short **description** of the incident (in block letters):

25. Who observed the incident? (*multiple answers possible*)?

Physician / staff ☐ patient / relatives ☐ community nurse ☐ home / institution ☐ hospital ☐

other physicians ☐ pharmacist ☐ other ☐ unknown ☐

for „other" please specify: ……………………………

26. What happened (*multiple answers possible*)?

dosage too high ☐

dosage too low ☐

application too short ☐

application too long ☐

wrong administration route ☐

wrong medication ☐

indicated medication not received ☐

expired / defective medication ☐

problems with insurance reimbursement ☐

unclear / undefined ☐

other (please specify): ……………………… ☐

27. Please state the **trade name** of the medication used in the incident:

………………………………………………..

36

For peer review only

28. Please note other medication names, presuming they are relevant to the case.

none ☐ unknown ☐

29. How would you judge the degree of hazard to the patient during the incident?

mild ☐ medium ☐ severe ☐ none ☐ does not apply ☐ unknown ☐

30. How intense was the impairment (as judged by the patient)?

mild ☐ medium ☐ severe ☐ fatal ☐ no symptoms, but pathological lab values ☐
no impairment ☐ does not apply ☐ unknown ☐
If there wasn't *any* impairment, please skip to **question 34**.

31. How long did the impairment last?

hours ☐ days ☐ weeks ☐ longer ☐ unknown ☐

32. How was the recovery?
without residues ☐ with mild residues ☐ with severe residues / fatal ☐ unknown ☐

33. Which organ system was affected (*multiple answers possible*)?
cardiovascular ☐
central nervous ☐
gastro-enteral ☐
kidneys ☐
liver ☐
lung ☐
skin ☐
other, please specify ………………… ☐

For peer review only

34. Did you have to apply a specific surveillance or treatment for the incident?

yes, ambulatory ☐ yes, hospital ☐ no ☐ unknown ☐

If yes, please specify which? ……………………………………………..

35. What factors contributed causally to the emergence of the incident (*multiple answers possible*)?

off duty hours ☐

communication failure within practice ☐

generic substitution by pharmacist ☐

hand-written prescription incorrectly interpreted ☐

conflicting multiple prescriptions ☐

lack of alertness within practice ☐

lack of documentation ☐

insufficient patient instruction ☐

lack of aids (e.g. Dosette®) ☐

lack of cooperation by patient / relatives ☐

misleading package leaflet information ☐

patient's internet search ☐

administrative problems ☐

manufacturer related (defective medication) ☐

distributer related (out of stock) ☐

lack of maintenance (e.g. first aid kit) ☐

other, please specify……………………….. ☐

unknown ☐

36. Was there an interface problem? If yes, which (*multiple answers possible*)?

yes, with hospital ☐

yes, with institution ☐

yes, with community nurse ☐

yes, with pharmacist ☐

yes, with specialist physician ☐

yes, with other, please specify? …………… ☐

For peer review only - http://bmjopen.bmj.com/site/about/guidelines.xhtml

no ☐

If **no**, please skip to **question 38**.

37. Was there an **explicit comparison** of prescription lists with the institution / person?

yes, verbal / by phone ☐ yes, written / by fax ☐ no ☐ unknown ☐

38/39. Was the patient informed about the incident?

yes, by myself / practice staff ☐
no, because he was not able to understand the message (children, demented) ☐
no, because the problem was solved and communication would have impaired confidence ☐
no, because the patient had moved or was deceased ☐
no, this was not needed because patient / relatives themselves had observed the incident ☐
no, because others had already informed him ☐

no, because: ……………………………………………………………………………. ☐
unknown ☐

If yes, what was the patient's reaction? …………………………………………………….

40. What did you do as a result to prevent similar incidents in the future (*multiple answers possible*)?

change standard operations procedures ☐
better instruction of patients ☐
communication with institution(s) ☐
notification of manufacturer ☐
notification of liability insurer ☐
notification of drug authority („yellow leaflet") ☐
notification of the "critical incident reporting system" ☐
other, please specify? ……………………….. ☐
nothing ☐

41. Who or what was ultimately **responsible** for the occurrence of the incident?
…………………………………………..

For peer review only

42. In the given situation, do you think one could have **anticipated** the event?

yes ☐ no ☐

43. Did you already report an **identical** or very similar incident to this study?

yes ☐ no ☐

44. Please make any **suggestions** about the kind of measures that could be taken to generally reduce the frequency of such events (in block letters):

Please keep a copy of this questionnaire in the patient files. Thank you for filling it out!

For peer review only

Appendix B Initial questionnaire

Medication incidents in primary care (MIPC)

Initial reporting (Version 1.0, January 25th 2015)
Internet version: not available in English

1. Sentinella identification number: ………….

2. Number of physicians within your practice: ………….

3. Number of them who report to Sentinella ………….

4. Your weekly workload, h/w: <16 ☐, 16-30 ☐, >30 ☐

5. Number of hours within your practice (%): <50% ☐, ≥50% ☐

6. Approximate proportion of medication prescribed (as compared to directly delivered drugs):
<33% ☐, 33-66% ☐, >66% ☐

7. Do you have an X-ray (machine?)? yes ☐ no ☐

8. Do you have an ECG? yes ☐ no ☐

9. Do you have an ultrasound? yes ☐ no ☐

10. Do you have an electronic system for controlling electronic drug interaction?
yes ☐ no ☐

11. Do you have electronic patient history documentation?
yes ☐ no ☐

12. Do you prescribe electronically?
yes, with a medication thesaurus ☐ yes, but without one (use of a typewriter) ☐ no ☐

13. Is your practice certified (e.g. EQUAM)? yes ☐ no ☐

14. Do you regularly schedule team sessions?
Yes, at least monthly ☐, yes, but less frequently ☐, no ☐

For peer review only

For peer review only - http://bmjopen.bmj.com/site/about/guidelines.xhtml

15. Do you attend quality circle sessions (in accordance with "Hausärzte Schweiz")?
yes, regularly ☐, yes, now and then ☐, no ☐

16. Did you complete a special education (e.g. manual or psychosomatic medicine), or do you have special interests (e.g. toxic maniac patients)? yes ☐, no ☐
if yes, please specify : ……………………………………………………………………….

17. Are you contracted by an institution? yes ☐ no ☐
if yes, please specify (prison, home etc.): ………………………………………………………………

18. If yes, does this institution have specific problems with medication?
yes ☐, no ☐, if yes, please specify: …………………………………………………………

19. Are you involved in other special activities (teaching, research, insurance doctor)?
yes ☐ no ☐
If yes, please specify the kind of activity: …………………………………………………..

Thank you very much!

For peer review only

For peer review only - http://bmjopen.bmj.com/site/about/guidelines.xhtml

Appendix C Final questionnaire

Medication Incidents in Primary Care (MIPC)

Final questionnaire (Version 3.0 / December 14th 2015)

Online version: not available in English

Dear colleagues,

Thank you for your dedicated support of our study on medication incidents in 2015, regardless of whether you reported much of them or not. We would like to ask some final questions which will help us put the other information you sent us in the right context.

1. Sentinella identification number: ……………

2. Did you **not** report medication incidents that you had noticed during the last year (e.g. because of lack of time)?

never or almost never ☐, yes, but rarely ☐, yes, frequently ☐ always or almost always ☐

If this was frequently the case, please explain why:………………………………………..

3. Did your practice participate in the fortnight morbidity denominator study in March 2015 (calendar weeks 11 and 12)?
yes, fully ☐, yes, *but only partly (by omission of certain variables)* ☐, no ☐

If you did not fully participate or not at all, what was the reason?
…………………………………………………………

If you did not participate in the denominator study, please continue with **question 12**.

4. How big was your effort for coding the morbidity variables of the denominator study?
manageable ☐, rather big ☐, too much ☐, impossible ☐

Did you have any difficulties when coding the morbidity variables?

5. Hospitalisation during the previous 12 months? none ☐, a little ☐, considerable ☐, severe ☐

6. Care-dependency? none ☐, a little ☐, considerable ☐, severe ☐

7. Number of medications? none ☐, a little ☐, considerable ☐, severe ☐

8. Number of conditions? none ☐, a little ☐, considerable ☐, severe ☐

9. Thurgau Morbidity Index? none ☐, a little ☐, considerable ☐, severe ☐

10. Repeat consultation during the fortnight? none ☐, a little ☐, considerable ☐, severe ☐

If you named considerable to severe difficulties in questions 5 to 10, please list them in **item 13**.

For peer review only - http://bmjopen.bmj.com/site/about/guidelines.xhtml

11. How long did it take for you and your practice nurse together for coding all of the variables of **one patient** (Dr. Gnädinger needed less than 3 minutes)?
……………………………………………….. minutes

12. Would you be willing to be interviewed for a focus group on the subject of medication safety?

yes ☐, no ☐

13. Other comments:

Thank you very much!

For peer review only

For peer review only - http://bmjopen.bmj.com/site/about/guidelines.xhtml

Appendix D: electronic tables and figures

Figure e1: Distribution of the incident notifications over the year
Table e1: What went wrong with the incidents
Table e2: Organ system involved
Table e3: Causes of the incident
Table e4: Patient-sided possible risk factors
Table e5: Reactions to the incident
Table e6: Proposals to avoid further incidents

For peer review only

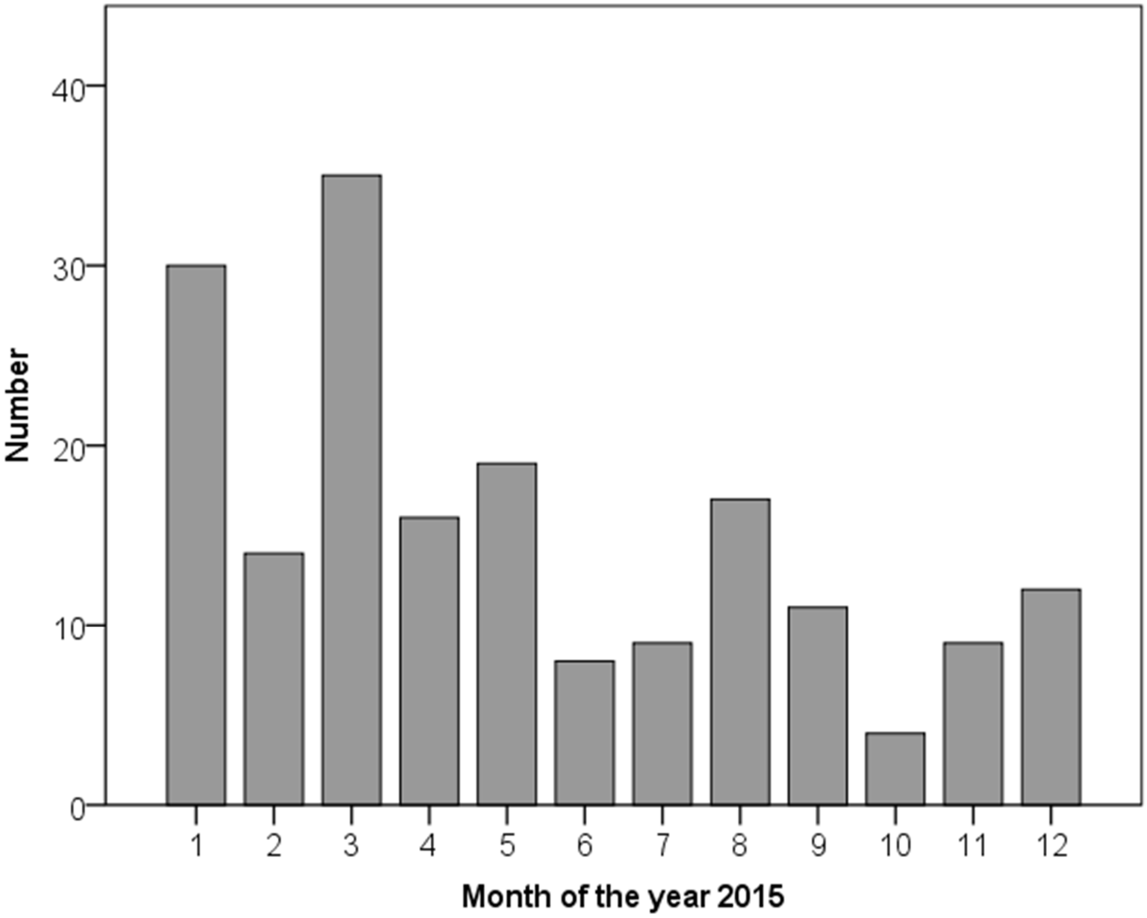

Figure e1. Distribution of the incident notifications over the year. Because only the week has been reported, the assignment to the month is approximate.

For peer review only - http://bmjopen.bmj.com/site/about/guidelines.xhtml

Type of error	N*	Percent*, %
Dosing too high	41 (12)	20.9 (6.1)
Dosing too low	21 (6)	10.7 (3.0)
Dosing too short	1 (2)	0.5 (1.0)
Dosing too long	5 (4)	2.6 (2.0)
Wrong way of administration	1 (1)	0.5 (0.5)
Wrong medication applied	56 (12)	28.6 (6.1)
Necessary medication not applied	12 (9)	6.1 (4.6)
Defective or expired medication applied	1 (1)	0.5 (0.5)
Problems with insurance reimbursing	1 (0)	0.5 (0.0)
Other problem**	31 (6)	15.8 (3.0)
Unknown	1 (0)	0.5 (0.0)
Multiple naming	26 (n.a.)	13.3 (n.a.)
Total	197 (53)	100.0 (26.9)

Table e1. What went wrong with the incidents. * Parenthesis denotes the additional naming within the category "multiple naming". ** Naming within the category "other" were: confusion of similar trade names (2), dosing error (6), erroneous package size (1), confusion of similar looking preparations (1), error when controlling blood levels (2), double dosing (4), missed discontinuation of medication (1), necessary treatment not applied (1), wrong vaccine applied (4), missed re-uptake of anticoagulation after operation (1), contra-indication overlooked (2), drug-drug interaction overlooked (3), known intolerance overlooked (3), necessary monitoring missed (2), transgression of maximal drug allowance (2), delivery of incomplete package (1), confusion by contradictory medication lists (3), incorrect handling of administering device (1), uncontrolled taking of "nature medicines" (1), unreliable compliance with medication plan (2), two medications of the same drug

For peer review only

For peer review only - http://bmjopen.bmj.com/site/about/guidelines.xhtml

class (3), forbidden bisection of pills (2), confusion of similar named patients (1), intake of medication of the neighbor resident in a home (1), other (9).

For peer review only

Organ system	N*	Percent*, %
Cardiovascular	14 (10)	15.4 (11.0)
Central nervous system	23 (10)	25.3 (11.0)
Gastro-intestinal	5 (3)	5.5 (3.3)
Kidneys	2 (2)	2.2 (2.2)
Liver	2 (0)	2.2 (2.2)
Lung	2 (1)	2.2 (1.1)
Skin	6 (0)	6.6 (0.0)
Other**	24 (6)	26.4 (6.6)
Multiple naming	13 (n.a.)	14.3 (n.a.)
Total	91 (32)	100 (35.2)
Question does not apply	106	/

Table e2. Organ system involved. * Parenthesis denotes the additional naming within the category “multiple naming”. ** Naming within the category “other” were: endocrine system (9), musculoskeletal (8), ear-nose-throat (2), psychic (1), other (9).

For peer review only

Causes of the incident	N*	Percent*;%
Out of hours	2 (3)	1.0 (1.5)
Communication problems within staff	10 (11)	5.1 (5.6)
Generic substitution of original trade medication by pharmacist	3 (3)	1.5 (1.5)
Difficulties when reading hand-written prescription	1 (1)	0.5 (0.5)
Multiple conflicting prescriptions	5 (8)	2.5 (4.1)
Lacking alertness of physician or practice staff	50 (17)	25.4 (8.6)
Insufficient documentation	3 (11)	1.5 (5.6)
Insufficient patient instruction	7 (17)	3.6 (8.6)
Lacking cooperation of patient / proxies	6 (17)	3.0 (8.6)
Confusion by reading package leaflet	1 (0)	0.5 (0.0)
Confusion after "Googleing"	1 (0)	0.5 (0.0)
Administrative problems	2 (3)	1.0 (1.5)
Defective medication as caused by manufacturer	3 (0)	1.5 (0.0)
Lacking maintenance (e.g. emergency case)	1 (0)	0.5 (0.0)
Lacking use of treatment aids (e.g. Dosett)	0 (6)	0.0 (3.0)
Other source of trouble**	54 (18)	27.2 (9.1)
Unknown	3 (0)	1.5 (0.0)
Multiple naming	45 (n.a.)	23.1 (n.a.)
Total	197 (115)	100.0 (58.4)

Table e3. Causes of the incident. * Parenthesis denotes the additional naming within the category "multiple naming". ** Naming within the category "other" were: Erroneous delivery in pharmacy (5), treatment delayed/hampered by patient (2), transcription error (2), missed discontinuation of an ongoing treatment (2), communication problem within helpers' network (16), similar trade names (2), inter-

For peer review only

face problems with hospital (4), reading error of patient / proxies (3), erroneous execution of a medical prescription (14), missed follow-up control (9), difficult handling of a preparation (2), disregard of possible interactions (2), transgression of competence by care workers (5), incomplete information by patient / proxies (8), stress / lack of time (12), other 25.

For peer review only

For peer review only - http://bmjopen.bmj.com/site/about/guidelines.xhtml

Item (number of missing observations in groups "less" / "more")	Relevance		All n (%)
	less n (%)	more n (%)	
Number of patients	124 (100.0)	73 (100.0)	197 (100.0)
Living situation (1/0)			
- together with mate / family	61 (49.6)	31 (42.5)	92 (46.9)
- alone	28 (22.8)	23 (31.5)	51 (26.0)
- institution	33 (27.6)	19 (26.0)	53 (27.0)
Social problems (2/4)			
- yes	22 (18.1)	18 (26.1)	49 (20.9)
Dementia or mental illness (2/2)			
- yes	31 (25.4)	18 (25.4)	49 (25.4)
Psychiatric problems (1/2)			
- yes	28 (22.8)	28 (38.4)*	56 (28.9)
Treatment with psychotropic drugs (0/0)			
- yes	50 (40.3)	39 (53.4)	89 (44.2)
Linguistic problems (0/1)			
- yes	11 (8.9)	5 (6.8)	16 (8.2)
Smoking (7/4)			
- yes	11 (9.4)	8 (11.6)	19 (10.2)
Substance abuse other than nicotine (2/1)			
- yes	3 (2.5)	5 (6.9)	8 (4.1)
Visual blurring (6/6)			
- yes	4 (3.4)	4 (6.0)	8 (4.3)
Hearing problems (3/5)			

For peer review only

- yes	8 (6.6)	4 (5.9)	12 (6.3)
Gait problems (2/2)			
- yes	40 (32.8)	18 (25.4)	58 (30.1)
Renal insufficiency (GFR<60 ml/min*1.73m^2) (6/2)			
- yes	24 (20.3)	21 (29.6)	45 (23.8)
Liver cirrhosis of other hepatic function problem (3/1)			
- yes	3 (2.5)	3 (4.2)	6 (3.1)

Table e4. Patient-sided possible risk factors. * p=0.021 "more" vs. "less" by chi-square testing. We calculated the variable "incident relevance" from the variables "disturbance" and "endangering" of the patients; if any of them was graded with "medium" or higher, the variable relevance was set to "more", otherwise to "less".

For peer review only

Reactions to the incident	N*	Percent*; %
Changing standard operating procedures of the practice	14 (3)	7.2 (1.5)
Better instruction of patients	23 (4)	11.7 (2.0)
Communication with other institutions	31 (3)	15.7 (1.5)
Notifying manufacturer	1 (2)	0.5 (1.0)
Reporting the incident to the critical incident reporting system	10 (6)	5.1 (3.0)
No reaction at all	55 (n.a.)	27.9 (n.a)
Other type of reactions to the incident**	49 (9)	24.9 (4.6)
Missing information	1 (0)	0.5 (0.0)
Multiple naming	13 (n.a.)	6.6 (n.a.)
Total	197 (27)	100.0 (13.7)

Table e5. Reactions to the incident. * Parenthesis denotes the additional naming within the category “multiple naming”. ** Naming within the category “other” were: Hire more workforce (1), arrangements with other physicians (2), with pharmacist (4), with community nurse (4), with institution (12), with practice nurse (6), with specialist (1), with patient (3), with supplier (1), sending a new medication plan (1), having regular staff meetings (1), remove Digoxin 0.25 from assortment because of safety reasons (1), to not perform vaccinations in absence of vaccination card (2), to clarify intolerances (2), to clarify interactions (1), to hand out an allergy card (1), to actualize patient records (3), to apply for insurance cost credit (1), to organize follow-up controls (20), other (5).

For peer review only

Proposal	N*	Percent*;%
Cross-check of medication lists	6 (14)	4.8 (11.2)
Patient instructions	2 (3)	1.6 (2.4)
Reduction of time pressure / stress	3 (6)	2.4 (4.8)
To observe adverse drug reactions also with “nature products”	1 (0)	0.8 (0.0)
Four eyes check when dispensing medication	2 (4)	1.6 (3.2)
No medication without prescription	3 (6)	2.4 (4.8)
To critically audit polymedication	3 (2)	2.4 (1.6)
To avoid similarly looking or named medication in drug master	2 (3)	1.6 (2.4)
To demand medication plans to be brought to the consul-tation	0 (4)	0.0 (3.2)
To provide medication plans routinely	2 (8)	1.6 (6.4)
In-deep checking before delivering “new” medication to the patient	4 (1)	3.2 (0.8)
To provide patients with allergy / intolerance cards	2 (1)	1.6 (0.8)
To let patients themselves write their medication card (and controlling afterwards)	0 (3)	0.0 (2.4)
To instruct patients to come into the practice immediately after hospitalization	3 (0)	2.4 (0.0)
To broach regularly “medication safety” on staff meetings	5 (6)	4.0 (4.8)
To provide information in patients’ native language	0 (2)	0.0 (1.6)
To share important information about patients with prac-tice nurse	0 (2)	0.0 (1.6)
To improve information flow	11 (16)	8.8 (10.4)
To organize follow-up controls	10 (28)	8.0 (22.4)
To wait for laboratory results before prescribing	1 (0)	0.8 (0.0)
Other**	12 (9)	9.6 (7.2)
Multiple answering	53 (n.a.)	42.4 (n.a.)
Total	125 (118)	100.0 (94.4)

For peer review only

None	72 (n.a.)	(n.a.)

Table e6. Proposals to avoid further incidents. * Parenthesis denotes the additional naming within the category “multiple naming”. ** Among naming within the category “other” were: timely involving of community nurse, stopping of self-medication in multimorbid or poly-medicated patients, to clearly labelling desensitization suspensions, to separating adult and pediatric vaccines, to implementing timely new guidelines, to refusing to be in care of a patient with bad compliance, to clearly separating and labelling of medication prepared for different patients, to advice patients for separating short- and long-acting insulins at their home, to providing short-time prescriptions with a clear expiration date, to allowing only adequately educated people to align and deliver medication in homes for the elderly and that delivering may be done by the same person who did the preparing of medication boxes, to avoiding similar looking medication boxes being muddled-up by patients at the refectory room.

For peer review only

For peer review only - http://bmjopen.bmj.com/site/about/guidelines.xhtml

Appendix E

STROBE Statement—Checklist of items that should be included in reports of ***cross-sectional studies***

	Item No	Recommendation
**Title and abstract**	1	(*a*) Indicate the study's design with a commonly used term in the title or the abstract **[yes]**
		(*b*) Provide in the abstract an informative and balanced summary of what was done and what was found **[yes]**
**Introduction**		
Background/rationale	2	Explain the scientific background and rationale for the investigation being reported **[yes]**
Objectives	3	State specific objectives, **[yes]** including any prespecified hypotheses **[no]**
**Methods**		
Study design	4	Present key elements of study design early in the paper **[yes]**
Setting	5	Describe the setting, locations, and relevant dates, including periods of recruitment, exposure, follow-up, and data collection **[yes]**
Participants	6	(*a*) Give the eligibility criteria, and the sources and methods of selection of participants **[yes]**
Variables	7	Clearly define all outcomes, exposures, predictors, potential confounders, and effect modifiers. **[yes]** Give diagnostic criteria, if applicable **[no]**
Data sources/ measurement	8*	For each variable of interest, give sources of data and details of methods of assessment (measurement). Describe comparability of assessment methods if there is more than one group **[yes]**
Bias	9	Describe any efforts to address potential sources of bias **[yes]**
Study size	10	Explain how the study size was arrived at **[yes]**
Quantitative variables	11	Explain how quantitative variables were handled in the analyses. If applicable, describe which groupings were chosen and why **[yes]**
Statistical methods	12	(*a*) Describe all statistical methods, including those used to control for confounding **[yes]**
		(*b*) Describe any methods used to examine subgroups and interactions **[yes]**
		(*c*) Explain how missing data were addressed **[yes]**
		(*d*) If applicable, describe analytical methods taking account of sampling strategy **[no]**
		(*e*) Describe any sensitivity analyses **[no]**

Results

For peer review only

Participants	13*	(a) Report numbers of individuals at each stage of study—eg numbers potentially eligible, examined for eligibility, confirmed eligible, included in the study, completing follow-up, and analysed **[no]**
		(b) Give reasons for non-participation at each stage **[yes]**
		(c) Consider use of a flow diagram **[yes]**
Descriptive data	14*	(a) Give characteristics of study participants (eg demographic, clinical, social) and information on exposures and potential confounders **[yes]**
		(b) Indicate number of participants with missing data for each variable of interest **[yes]**
Outcome data	15*	Report numbers of outcome events or summary measures **[yes]**
Main results	16	(*a*) Give unadjusted estimates and, if applicable, confounder-adjusted estimates and their precision (eg, 95% confidence interval). Make clear which confounders were adjusted for and why they were included **[yes]**
		(*b*) Report category boundaries when continuous variables were categorized **[yes]**
		(*c*) If relevant, consider translating estimates of relative risk into absolute risk for a meaningful time period **[no]**
Other analyses	17	Report other analyses done—eg analyses of subgroups and interactions, and sensitivity analyses **[yes]**
**Discussion**		
Key results	18	Summarise key results with reference to study objectives **[yes]**
Limitations	19	Discuss limitations of the study, taking into account sources of potential bias or imprecision. Discuss both direction and magnitude of any potential bias **[yes]**
Interpretation	20	Give a cautious overall interpretation of results considering objectives, limitations, multiplicity of analyses, results from similar studies, and other relevant evidence **[yes]**
Generalisability	21	Discuss the generalisability (external validity) of the study results **[yes]**
**Other information**		
Funding	22	Give the source of funding and the role of the funders for the present study and, if applicable, for the original study on which the present article is based **[yes]**

*Give information separately for exposed and unexposed groups.

Note: An Explanation and Elaboration article discusses each checklist item and gives methodological background and published examples of transparent reporting. The STROBE checklist is best used in conjunction with this article (freely available on the Web sites of PLoS Medicine at http://www.plosmedicine.org/, Annals of Internal Medicine at http://www.annals.org/, and Epidemiology at http://www.epidem.com/). Information on the STROBE Initiative is available at www.strobe-statement.org

For peer review only

For peer review only - http://bmjopen.bmj.com/site/about/guidelines.xhtml

Appendix F

Swiss Pharmaceutical Market: Number of packaged sold by ATC-groups, 2015

	2015 number of packaged sold
Total	210,992,389
A Alimentäres System und Stoffwechsel	31,455,252
A01 Stomatologika, Medizinische Präparate zur Mund- und Zahnpflege	1,473,520
A02 Antacida-Ulcustherapeutika, Antiflatulentia	6,051,251
A02A Antacida,Antiflatulentia	1,500,822
A02B Ulcustherapeutika	4,426,496
A02C Sonstige Magentherapeutika	123,933
A03 Produkte gegen funktionelle Magen-Darm-Störungen	2,087,299
A03A Antispasmodika+Anticholinergika rein	595,861
A03C Antispasmodika/Ataraktika-Kombinationen	25,411
A03E Antispasmodika, sonstige Kombinationen	222,102
A03F Gastroprokinetika	1,233,461
A03G Modulatoren der gastrointestinalen Sensomotorik	10,464
A04 Antiemetica und Antinausea	920,841
A05 Cholagoga und Leberschutz-Mittel	337,970
A05A Gallentherapeutica und Cholagoga	254,744
A05B Leberschutzpräparate	83,226
A06 Mittel gegen Verstopfung und Darmreinigung	4,583,118
A06A Mittel Gegen Verstopfung	4,329,037
A06B Darmreinigungsmittel	254,081
A07 Antidiarrhoica, Elektrolytzufuhr und intestinale Antiphlogistica	3,005,035
A07B Intestinale Adsorbierende Antidiarrhoica	228,076
A07E Intestinale Antiphlogistica	222,680
A07F Antidiarrhoica Micro-Organismen	1,340,432
A07G Orale Elektrolyt-Zufuhr	107,030
A07H Motilitätshemmer	1,095,216
A07X Übrige Antidiarrhoica inkl. A07A Antidiarrhoica intestinale Antiinfectiva	11,601
A08 Antiadiposita excl. Diätetica	51,654
A09 Digestiva und Enzyme	804,667
A10 Antidiabetica	3,376,580
A10C Humaninsulin und Analoga	1,078,495
A10H Sulfonylharnstoff Antidiabetika	321,219
A10J Biguanid Antidiabetika	1,217,578
A10K Glitazone Antidiabetika	25,305
A10M Glinide Antidiabetika	38,782
A10N DPP-IV Inhibitor Antidiabetika	483,559
A10P SGLT2-Hemmer Antidiabetika	45,927
A10S GLP-1 Agonisten Antidiabetika	156,656
A10X Übrige Antidiabetika inkl. Insulin tierischen Ursprungs und Alphaglukosidaseinhibitor Antidiabetika	8,616

A11 Vitamine	5,001,408
A11A Multivitamine mit Mineralstoffen	755,243
A11B Multivitamine ohne Mineralstoffe	17,507
A11C Vitamin A+D inkl. einfache Kombinationen	2,618,699
A11D Vitamin B1 und Kombinationen	170,884
A11E Vitamin B-Komplex	739,860
A11F Vitamin B 12 rein	123,445
A11G Vitamin C, inkl. Kombinationen mit Mineralstoffen	353,644
A11X Sonstige Vitamine	222,126
A12 Mineralverbindungen	3,490,891
A12A Mineralverbindung Calcium	1,254,334
A12B Mineralverbindung Kalium	200,438
A12C Sonstige Mineralverbindungen	2,036,119
A13 Tonica und Roborantia	229,735
A16 Übrige Präparate des alimentären und Stoffwechsel-Systems	34,295
B Blut und Blutbildende Organe	5,507,624
B01 Antithrombotika	4,009,465
B01A Vitamin-K-Antagonisten	399,963
B01B Heparine	714,872
B01C Thrombozytenaggregationshemmer	2,265,838
B01D Fibrinolytika	14,293
B01X Direkte Thrombin-Inhibit und direkte Faktor XA-Hemmber	598,766
B02 Blutgerinnungssystem – Sonstige Produkte	237,495
B02B Antagonisten (Antidot.Anticoag)	146,201
B02D Blutcoagulation	1,805
B02G Glitazone-Antidiabetika	48,969
B02X Übrige Blutgerinnungsprodukte (Tissues sealing preparations, Thrombopoietin Agonisten, Antifibrinolytica synthetisch etc.)	40,520
B03 Antianämica	1,259,896
B03A Antianämica mit Eisen und Kombinationen	862,744
B03C Erythropoietin-Präparate	67,258
B03X Sonstige Antianämica inkl. Folsäure/Folinsäure	329,894
C Herz-Kreislauf Therapie	16,027,143
C01 Cardica	954,962
C01B Anti-Arrhythmie-Präparate	213,952
C01C Cardiale Stimulantien excl. Herzglykoside	266,028
C01D Coronartherapeutica excl. Calcium-Antagonisten und Nitropräparate	121,815
C01E Nitropräparate und Analog	228,438
C01X Positive Inotrope, Herzglykoside und übrige Herzpräparate	124,729
C02 Antihypertonica	129,957
C03 Diuretica	1,815,738
C04 Cerebrale und periphere Vasotherapeutika	390,255
C05 Antivaricosa/Antihaemorrhoidalia	2,271,175
C05A Antihaemorrhoidalia topisch	773,800
C05B Antivaricosa topisch	1,029,010
C05C Antivaricosa systemisch	468,365
C06 Übrige Herz+Kreislaufmittel	339,930
C07 Betablocker	2,398,309
C07A Betablocker rein	2,272,988

For peer review only - http://bmjopen.bmj.com/site/about/guidelines.xhtml

C07B Betablocker Kombination	125,321
C08 Calciumantagonisten	1,157,267
C09 Stoffe mit Wirkung auf das Renin-Angiotensin-System	4,116,677
C09A Ace-Hemmer, rein	1,142,465
C09B Ace-Hemmer, Kombinationen	584,697
C09C Angiotensin-II-Antagonisten, rein	1,084,218
C09D Angiotensin-II-Antagonisten, Kombinationen	1,261,616
C09X Sonstige Stoffe mit Wirkung auf das Renin - Angiotensin - System	43,681
C10 Lipidregu/Antiarteriosklerotika	2,446,927
C10A Lipidregulatoren	2,341,963
C10A1 Statine (Cholesterolsynthesehemmer)	2,190,227
C10A2 Lipidsenker, Fibrate, Ionenaustauscher und sonstige Lipidsenker	151,736
C10C Lipidregulatoren kombiniert mit anderen Lipidregulatoren	104,964
C11 Übrige Präparate für die Herz-Kreislauf-Therapie	5,946
D Dermatologica	17,314,810
D01 Dermatologische Antimykotica	1,360,570
D02 Emollientia und Hautschutzmittel	2,703,273
D03 Wundheilmittel	4,389,791
D04 Antipruriginosa inkl. Antihistaminica und Anästhetica usw.	1,458,276
D05 Nichtsteroidale Präparate gegen entzündliche Hauterkrankungen	327,245
D06 Topische antibakterielle Produkte und antivirale Mittel	864,829
D06A Topische antibakterielle Produkte	379,013
D06D Topische Produkte gegen Virusinfektionen	485,816
D07 Corticosteroide topisch	1,982,173
D07A Reine Corticosteroide topisch	960,182
D07B Corticosteroid-Kombinationen topisch	1,021,991
D08 Antiseptica + Desinfizientia	1,836,788
D10 Aknemittel	1,227,850
D10A Topische Aknemittel	829,114
D10B Orale Aknemittel	398,736
D11 Übrige Dermatologica	1,164,015
G Urogenital-System+Sexualhormon	7,936,641
G01 Gynaekologische Antiinfektiva	812,985
G01A Trichomonadenmittel	185,395
G01B Gynaekologische Antimykotica	475,911
G01X Gynaekologische antibakterielle Produkte und Antiseptika	151,679
G02 Sonstige Gynaekologica	1,060,376
G02A Oxytocica	91,111
G02F Topische Sexualhormone	496,735
G02X Lokale Antikonzipientia, Prolactininhibitoren, wehenhemmende Mittel und sonstige Gynaekologika	472,530
G03 Sexualhormone und Stimulantien des Genitalsystems	3,917,793
G03A Hormonale Kontrazeptiva systemisch	2,477,919
G03B Androgene, excl.G3E,G3F	70,852
G03C Oestrogene, excl.G3A,G3E,G3F	397,050
G03D Progestogene, excl. G3A,G3F	245,149
G03F Oestrogen-Progestogen-Kombinationen	487,291
G03G Gonadotropin inl. sonstige Ovulationsstimulantien	100,879
G03X Androgen-Kombinationen mit weiblichen Sexualhormonen, SERMS und	138,653

sonstige Sexualhormone und ähnliche Produkte	
G04 Urologica	2,145,487
G04A Urologische Antiinfektiva und Harnantiseptika	410,545
G04C BPH Produkte (Benigne Prostata-Hyperplasie)	715,845
G04D Harninkontinenzprodukte	390,832
G04E Produkte gegen Erektionsstörungen	311,659
G04X Alle sonstigen Urologika	316,606
H Hormonales System, exkl. Sexualhormone	2,875,760
H01 Hypophysen-/Hypothalamus-Hormone	36,944
H02 Corticosteroide Systemisch	1,575,880
H03 Schilddruesentherapie	1,109,934
H04 Sonstige Hormone (wachstumshormone, antidiuretische Hormone, Anti-Parathyroid-Hormone etc.)	153,002
J Antiinfektiva systemisch	8,446,623
J01 System.Antibakter.Prod.	5,721,173
J01A Tetracycline und Kombinationen	155,659
J01C Breitspektrum Penicilline	2,353,136
J01D Cephalosporine	897,839
J01F Macrolide und vergleichbare Substanzen	778,785
J01G Fluorchinolone	817,056
J01H Mittel- und Schmalspektrum-Penicilline	111,484
J01K Aminoglykoside	46,447
J01P Sonstige Beta-Lactam. Antibiot. Penicilline, Cephalosporine (Monobactame, Peneme und Carbapeneme)	69,074
J01X Rifamycin und vergleichbare Substanzen, Trimethoprim, Kombinationen und vergleichbare Substanzen und sonstige antibakterielle Produkte	491,693
J02 Antimykotika, Systemisch	591,346
J04 Tuberkulosemittel	37,320
J05 Antivirale Mittel, systemisch	570,063
J05B Antivirale Mittel, exkl. Produkte gegen HIV	236,676
J05C Virustatika gegen HIV	333,387
J05C1 Nucleoside und Nucleotide Reverse Transkriptase Hemmer	108,107
J05C2 Protease Hemmer	98,581
J05CX Nicht-Nucleoside Reverse Transkriptase Hemmer, HIV Fusionshemmer, HIV-Antivirale Integrase Hemmer und sonst.HIV-Antivirale Mittel	126,699
J06 Sera und Gammaglobulin	23,164
J07 Vakzine	1,406,674
J07B Vakzine Kombinationen	463,631
J07B1 Kombinationen mit Tetanusimpfstoff	387,139
J07BX Mehrfach-Impfstoffe mit Masern und/oder Mumps und alle anderen Kombinationen	76,492
J07D Bakterielle Impfstoffe	164,542
J07E Virale Impfstoffe	687,366
J07E1 Influenza-Impfstoff	205,391
J07E4 Hepatitis-Impfstoff	212,503
J07E9 Alle uebrigen viralen Impfstoffe, inkl. HPV, Rotaviren, Varizellen etc.	269,472
J07X Alle uebrigen Impfstoffe und aehnliche Produkte	91,135
J08B Übrige Antiinfektiva	96,883
K Infusionslösungen	24,158,749
K01 Intravenoese Lösungen ab 100 ML	12,064,154
K01A Elektrolytlösungen	3,372,213

For peer review only - http://bmjopen.bmj.com/site/about/guidelines.xhtml

K01B Standardlösungen	8,461,628
K01D Fettemulsionen, inkl. Produkte zur totalen parenteralen Ernährung	156,484
K01E Aminosäuren-Lösungen	51,140
K01X Kalorische Lös. über 10% und Lösungen zur Osmotherapie	22,689
K02 Plasma Expander	70,547
K03 Blut- und Blutersatzinfusionslösungen	1,817
K04 Injektionslösungen/Infusionszusätze	12,005,621
K04A Elektrolytlösungen	752,499
K04B Standardlösungen	9,981,186
K04C Kalorische Lösungen < 100 ML	34,050
K04D Sonstige Injektionslösungen/Infusionszusätze < 100 ML	1,237,886
K05 Spüllösungen	16,610
L Antineoplastika und Immunmodulatoren	1,934,950
L01 Antineoplastika	984,072
L01A Alkylisierende Substanzen	46,698
L01B Antimetaboliten	323,741
L01C Vinca-Alkaloide & sonstige pflanzliche Produkte	157,793
L01D Antineoplastisch wirkende Antibiotika	46,619
L01F Platinhalt.Antineoplast.	95,347
L01G Monoklonale Antikörper zur antineoplastischen Therapie	159,208
L01H Proteinkinasehemmer zur antineoplastischen Therapie	33,895
L01X Sonstige Antineoplastika	120,771
L02 Cytostatische Hormonetherapie	183,379
L02A Cytostatische Hormone	43,050
L02B Cytostatische Hormonantagonisten	140,329
L03 Immunostimulantien	79,294
L03A Immunstimulantien	40,990
L03B Interferone	38,304
L04 Immunsuppressiva	688,205
L04B Anti-TNF-Produkte	340,857
L04X Sonstige Immunsuppressiva inkl. Interleukin Inhibitoren	347,348
M Muskel-und Skelettsystem	14,787,413
M01 Antirheumatica	6,373,707
M01A Corticoidfreie Antirheumatica	5,700,326
M01C Antirheumatica spezifisch	673,381
M02 Rheumaeinreibungen Rubefatientia	6,372,249
M03 Muskelrelaxantien	665,391
M03A Muskelrelaxantien peripher wirkend	85,757
M03B Muskelrelaxantien zentral wirkend	579,634
M04 Gichtmittel	368,020
M05 Übrigige Muskel-Skelettsystem-Präparate	1,008,046
N Nervensystem	42,690,195
N01 Anaesthetica	1,554,391
N01A Narkosemittel	1,078,794
N01A1 Narkosemittel Inhalationspräparate	20,582
N01A2 Narkosemittel Injektionspräparate	1,058,212
N01B Lokalanaesthetika	475,597
N01B1 Medizinische injizierbare Lokalanästhetika	198,964

For peer review only - http://bmjopen.bmj.com/site/about/guidelines.xhtml

N01B3 Topische Lokalanästhetika	270,962
N01B9 Sonstige Lokalanästhetika und dentale injizierbare Lokalanästhetika	5,671
N02 Analgetica	24,080,469
N02A Betäubungsmittel	1,661,607
N02B Analgetica und Antipyretica	21,918,098
N02B1 Analgetica rezeptpflichtig	8,592,792
N02B2 Analgetica rezeptfrei	13,325,306
N02C Migränemittel	500,764
N02C1 Triptane	432,741
N02C9 Sonstige Migränemittel	68,023
N03 Antiepileptica	1,957,745
N04 Parkinsonmittel	658,710
N05 Psycholeptica	9,075,955
N05A Antipsychotika	2,002,404
N05A1 Atypische Antipsychotika	1,521,618
N05A9 Konventionelle Antipsychotika	480,786
N05B Hypnotica und Sedativa	4,462,062
N05B1 Hypnotica und Sedativa, barbituratfreie Reinsubstanzen	2,830,382
N05B2 Hypnotica und Sedativa, barbituratfreie Kombinationen	129,461
N05B5 Pflanzliche Hypnotica und Sedativa	1,502,219
N05C Tranquilizer	2,611,489
N06 Psychoanaleptica Ex.Antiadipos	4,081,575
N06A Antidepressiva und stimmungsstabilisierende Produkte	3,503,509
N06A2 Pflanzliche Antidepressiva	245,372
N06A3 Stimmungsstabilisierende Produkte	50,396
N06A4 SSRI Antidepressiva	1,319,897
N06A5 SNRI Antidepressiva	664,070
N06A9 Sonstige Antidepressiva	1,223,774
N06B Psychostimulantia	341,172
N06X Psycholeptica-Psychoanaleptica-Kombinationen, Nootropica und Neurotonica sowie verschiedene andere Produkte	236,894
N07 Sonstige ZNS-wirksame Produkte	1,281,350
N07B Raucherentwöhnungsmittel	652,146
N07C Produkte gegen Schwindel	286,538
N07D Anti-Alzheimer Produkte	121,928
N07E Alkohol- und Opiatentwöhnungsmittel	126,973
N07X Alle anderen ZNS-wirksamen Präparate	93,765
P Parasitologie	462,559
P01 Antiprotozoenmittel und Anthelmintika	437,402
P01B Anthelmintica, excl. Schistosomiasis-Mittel	296,327
P01D Malariamittel	139,242
P02 Sonstige Antiparasitäre Präparate inkl. Insektizide und Repellentien	26,990
R Respirationssystem	28,837,468
R01 Rhinologica	7,246,454
R01A Rhinologica topisch	6,868,431
R01AX Corticosteroide Rhinologika	1,067,991
R01A6 Antiallergische Rhinologica	230,180
R01A7 Abschwellende Rhinologica	4,290,641
R01A9 Sonstige Rhinologica topisch inkl. Antiinfektive Rhinologika ohne	1,279,619

Corticosteroide	
R01B Rhinologica systemisch	378,023
R02 Halsschmerzmittel und Antiinfectiva	3,977,806
R03 Antiasthmatika und Produkte gegen chronisch-obstruktive Lungenerkrankungen	2,931,292
R03A B2-Stimulatoren	923,570
R03B Xanthine	16,188
R03C Degranulationshemmer	9,088
R03D Corticoide	258,075
R03F Komb. B2-Stimulatoren mit Corticoiden	1,172,896
R03G Anticholinergica rein und in Komb. mit B2-Stimulatoren	422,479
R03G3 Anticholinergica rein, Inhalate	269,418
R03G4 Anticholinergica – Kombinationen mit Beta-2-Stimulantien, Inhalate	153,061
R03J Antileukotrien Antiasthmatika	90,062
R03X Sonstige Antiasthmatika und Produkte gegen chronisch-obstruktive Lungenerkrankungen inkl. PDE4-Hemmer gegen Asthma / Chronisch-Obstruktive Lungenerkrankungen	38,934
R04 Percutane Einreibemittel und Inhalationspräparate	1,093,346
R05 Husten- und Erkältungspräparate	10,899,026
R05A Erkältungspräparate	2,498,594
R05C Expectorantia ohne Antiinfectiva	4,499,364
R05D Hustensedativa	3,297,144
R05D1 Hustenmittel plain (Einzelsubstanzen)	1,971,766
R05D2 Sonstige Hustensedativa, inkl. Kombinationen	1,325,378
R05F Sonstige Husten- und Erkältungspräparate	603,924
R06 Antihistamine systemisch	2,687,483
R07 Sonstige Präparate des Respirationssystems	2,061
S Sinnesorgane	7,658,311
S01 Ophthalmologica	7,214,097
S01A Ophtalmologische Antiinfectiva	451,604
S01B Ophtalmologische Corticoide rein	271,663
S01C Ophthalm. Antiphlogistika und Antiinfektiva Kombinationen	613,682
S01E Myotika und Mittel zur Glaukombehandlung	1,038,592
S01F Mydriatika und Cykloplegika	46,547
S01G Ophthalmologische Antiallergika, Abschwellende Mittel, Antiseptika	1,473,188
S01H Ophtalmolog. Lokalanesthetika	40,383
S01K Künstliche Tränen und Netzmittel für Augen	2,503,750
S01P Ophthalmologische Antineovaskularisationsprodukte	127,501
S01R Nonsteroidale entzündungshemmende Ophthalmologica (NSAID'S)	236,207
S01X Übrige Ophtalmologica inkl. Präparate für den Gebrauch von Kontaktlinsen, Augentonika und Augenvitamine, Präparate zur Verhütung von Katarakt und Antikataraktogenika, Ophthalmologische Operationshilfsmittel und Ophthalmologische Diagnostika	411,009
S02 Otologica	444,214
T Diagnostika	43,184
T01 Diagnostika für bildgebende Verfahren	18,885
T02 Testdiagnostika	24,299
V Verschiedenes	855,707
V01 Allergene	6,429
V03 Übrige Therapeutische Präparate	820,500
V03D Entgiftungspräparate für die Cytostatikatherapie	45,668

For peer review only - http://bmjopen.bmj.com/site/about/guidelines.xhtml

V03E Antidote	4,532
V03G Hyperkaliämie/Hyperphosphatämie-Produkte	42,259
V03X Sonstige therapeutische Präparate inkl. Entzündungshemmende Enzyme, Eisen-Chelatbildner und Radiopharmazeutika	728,041
V05 Chirurgische Antiseptica	134
V06 Allgemeine Nährmittel	5,612
V07 Alle übrigen nicht therapeutischen Präparate	23,032

Source: Interpharma with data from IMS Health Schweiz, 2016.

For peer review only

STROBE Statement—Checklist of items that should be included in reports of ***cross-sectional studies***

	Item No	Recommendation	Manu-script page
**Title and abstract**	1	(*a*) Indicate the study's design with a commonly used term in the title or the abstract **[yes]**	1
		(*b*) Provide in the abstract an informative and balanced summary of what was done and what was found **[yes]**	1-2
**Introduction**			
Background/rationale	2	Explain the scientific background and rationale for the investigation being reported **[yes]**	3
Objectives	3	State specific objectives, **[yes]** including any prespecified hypotheses **[no]**	3
**Methods**			
Study design	4	Present key elements of study design early in the paper **[yes]**	3
Setting	5	Describe the setting, locations, and relevant dates, including periods of recruitment, exposure, follow-up, and data collection **[yes]**	3
Participants	6	(*a*) Give the eligibility criteria, and the sources and methods of selection of participants **[yes]**	3
Variables	7	Clearly define all outcomes, exposures, predictors, potential confounders, and effect modifiers. **[yes]** Give diagnostic criteria, if applicable **[no]**	3
Data sources/ measurement	8*	For each variable of interest, give sources of data and details of methods of assessment (measurement). Describe comparability of assessment methods if there is more than one group **[yes]**	4
Bias	9	Describe any efforts to address potential sources of bias **[yes]**	4
Study size	10	Explain how the study size was arrived at **[yes]**	4-5
Quantitative variables	11	Explain how quantitative variables were handled in the analyses. If applicable, describe which groupings were chosen and why **[yes]**	4-5
Statistical methods	12	(*a*) Describe all statistical methods, including those used to control for confounding **[yes]**	5
		(*b*) Describe any methods used to examine subgroups and interactions **[yes]**	5
		(*c*) Explain how missing data were addressed **[yes]**	5
		(*d*) If applicable, describe analytical methods taking	n.a.

For peer review only

		account of sampling strategy [no]	
		(*e*) Describe any sensitivity analyses [no]	n.a.
**Results**			
Participants	13*	(a) Report numbers of individuals at each stage of study—eg numbers potentially eligible, examined for eligibility, confirmed eligible, included in the study, completing follow-up, and analysed [no]	5
		(b) Give reasons for non-participation at each stage [yes]	5
		(c) Consider use of a flow diagram [yes]	18
Descriptive data	14*	(a) Give characteristics of study participants (eg demographic, clinical, social) and information on exposures and potential confounders [yes]	22-24
		(b) Indicate number of participants with missing data for each variable of interest [yes]	5
Outcome data	15*	Report numbers of outcome events or summary measures [yes]	6
Main results	16	(*a*) Give unadjusted estimates and, if applicable, confounder-adjusted estimates and their precision (eg, 95% confidence interval). Make clear which confounders were adjusted for and why they were included [yes]	28
		(*b*) Report category boundaries when continuous variables were categorized n.a.	n.a.
		(*c*) If relevant, consider translating estimates of relative risk into absolute risk for a meaningful time period [no]	n.a.
Other analyses	17	Report other analyses done—eg analyses of subgroups and interactions, and sensitivity analyses [yes]	24
**Discussion**			
Key results	18	Summarise key results with reference to study objectives [yes]	8
Limitations	19	Discuss limitations of the study, taking into account sources of potential bias or imprecision. Discuss both direction and magnitude of any potential bias [yes]	2
Interpretation	20	Give a cautious overall interpretation of results considering objectives, limitations, multiplicity of analyses, results from similar studies, and other relevant evidence [yes]	8-11
Generalisability	21	Discuss the generalisability (external validity) of the study results [yes]	6
**Other information**			

For peer review only

Funding	22	Give the source of funding and the role of the funders for the present study and, if applicable, for the original study on which the present article is based **[yes]**	13

*Give information separately for exposed and unexposed groups.

Note: An Explanation and Elaboration article discusses each checklist item and gives methodological background and published examples of transparent reporting. The STROBE checklist is best used in conjunction with this article (freely available on the Web sites of PLoS Medicine at http://www.plosmedicine.org/, Annals of Internal Medicine at http://www.annals.org/, and Epidemiology at http://www.epidem.com/). Information on the STROBE Initiative is available at www.strobe-statement.org

For peer review only

Medication Incidents in Primary Care Medicine: a Prospective Study in the Swiss Sentinel Surveillance Network (Sentinella)

Journal:	*BMJ Open*
Manuscript ID	bmjopen-2016-013658.R1
Article Type:	Research
Date Submitted by the Author:	21-Nov-2016

Complete List of Authors:	Gnädinger, Markus; University of Zurich, Institute for General Medicine<br>Conen, Dieter; Swiss Patient Safety,<br>Herzig, Lilli; University of Lausanne, Institute of General Medicine<br>Puhan, Milo; University of Zurich, Institute of Epidemiology, Biostatistics & Prevention<br>Staehelin, Alfred; Sentinel Surveillance Network, Swiss Federal Office of Public Health,<br>Zoller, Marco; Zurich University Hospital, Instiute for General Medicine<br>Ceschi, Alessandro; National Poisons Centre, Tox Info Suisse, Associated Institute of the University of Zurich, Division of Science, Head of ; University Hospital Zurich , Dept. Clinical Pharmacology & Toxicology

**Primary Subject Heading**:	General practice / Family practice
Secondary Subject Heading:	Pharmacology and therapeutics, Paediatrics, Medical management, Nursing
Keywords:	Health & safety < HEALTH SERVICES ADMINISTRATION & MANAGEMENT, Adverse events < THERAPEUTICS, Patient safety, Pharmaceutical preparations, Medication errors

For peer review only

Medication Incidents in Primary Care Medicine

A Prospective Study in the Swiss Sentinel Surveillance Network (*Sentinella*)

[1]Markus Gnädinger (corresponding author, markus.gnaedinger@hin.ch), [2]Dieter Conen, [3,4]Lilli Herzig, [5]Milo A. Puhan, [1,4]Alfred Staehelin, [1]Marco Zoller, [6]Alessandro Ceschi

[1]Institute of Primary Care, University of Zurich, [2]Patientensicherheit Schweiz, Zurich [3]Policlinique Médicale, University of Lausanne, [4]Sentinel Surveillance Network, Swiss Federal Office of Public Health, Bern (*Sentinella*), [5]Epidemiology, Biostatistics, and Prevention Institute, University of Zurich, [6]Division of Clinical Pharmacology and Toxicology, Department of Internal Medicine, Ente Ospedaliero Cantonale, Lugano, Switzerland and Department of Clinical Pharmacology and Toxicology, University Hospital Zurich, Zurich, Switzerland and National Poisons Centre, Tox Info Suisse, Associated Institute of the University of Zurich, Zurich, Switzerland

(**Correspondence:** Markus Gnädinger, Dr. med. Facharzt für Innere Medizin, Birkenweg 8, 9323 Steinach, 0041 71 446 04 64)

Abstract

Objectives: To describe the type, frequency, seasonal and regional distribution of medication incidents in primary care in Switzerland and to elucidate possible risk factors for medication incidents.

Design: Prospective surveillance study.

Setting: Swiss primary health care, Swiss Sentinel Surveillance Network.

Participants: Patients with drug treatment who experienced any *erroneous* event related to the medication process and interfering with normal treatment course, as judged by their physician. The 180 physicians in the study were general practitioners or pediatricians participating in the Swiss Federal Sentinel reporting system in 2015.

Outcomes: *Primary:* medication incidents; *secondary:* potential risk factors like age, gender, poly-medication, morbidity, care-dependency, previous hospitalization.

For peer review only

Results: The mean rates of detected medication incidents were 2.07 per general practitioner per year (46.5 per 100,000 contacts) and 0.15 per pediatrician per year (2.8 per 100,000 contacts), respectively. The following factors were associated with medication incidents (OR, 95% CI): higher age 1.004 per year (1.001;1.006), care by community nurse 1.458 (1.025;2.073) , and care by an institution 1.802 (1.399;2.323) , chronic conditions 1.052 (1.029;1.075) per condition, medications 1.052 (1.030;1.074) per medication, as well as Thurgau Morbidity Index for stage 4: 1.292 (1.004;1.662), 5: 1.420 (1.078;1.868), and 6: 1.680 (1.178;2.396), respectively. Most cases were linked to an incorrect dosage for a given patient, while prescription of an erroneous medication was the second most common error.

Conclusions: Medication incidents are common in adult primary care whereas they rarely occur in pediatrics. Older and multimorbid patients are at a particularly high risk for medication incidents. Reasons for medication incidents are diverse but often seem to be linked to communication problems.

Trial registration: www.clinicaltrials.gov, NCT0229537

Keywords: Patient safety, pharmaceutical preparations, medication errors.

Strength and limitations

This is the first prospective and systematic collection of incident data in primary care in Switzerland that is characterized by three linguistic regions and two drug distribution systems.
It was conducted by experienced physicians and with high response rates.

There is likely – as expected – bias from selective and underreporting or non-detection of medication incidents.

Introduction

Patient safety is a major concern in healthcare systems worldwide. Although most safety research has been conducted in the in-patient setting [1], evidence indicates that medical errors and adverse events pose a serious threat for patients in the primary care setting as well, since most patients receive ambulatory care [2-4]. The rationale of this project has been published in our study protocol [5]. The *aim* of the project was to describe the type, incidence, seasonal and regional distribution of medication incidents in primary care in Switzerland and to elucidate risk factors for medication incident.

For peer review only

For peer review only - http://bmjopen.bmj.com/site/about/guidelines.xhtml

Method

Study design

We conducted a prospective surveillance study among primary care patients during 2015 to identify cases of medication incidents.

Study population

The study population was any person undergoing drug treatment in general internal or pediatric practices participating the *Sentinella* network. The latter covers a representative sample of patients in primary care for Switzerland [6, *included to this manuscript*]. Founded in 1986, it was mainly designed to survey transmissible diseases. Later, it also assessed other health problems of public interest. It generates daily to weekly current data and covers the entire geographic and linguistic regions of our country. Children, the mentally handicapped or the elderly were also included, all of whom might be at increased risk for medication errors.

Medication incidents

We defined medication incidents as any erroneous event (as defined by the physician) related to the medication process and interfering with normal treatment course (e.g. administration of an erroneous medication). We did not include lack of treatment effect, adverse drug reactions or drug-drug or drug-disease interactions without detectable treatment error. Nor did we consider medication incidents if patients refused to have them reported to the *Sentinella* system.

Data sources

The study physicians recorded the patient's year of birth and gender on their weekly reporting form. After a maximum of four weeks they had to fill in a detailed incident questionnaire (Appendix A). It was comprised of their *Sentinella* number and the calendar week of notification. Concerning the patients, they reported the living situation, several supposed risk factors for an incident, as well as the following variables: hospitalization during the previous year, care-dependency, number of drugs used chronically, number of chronic conditions, and the Thurgau-Morbidity-Index (TMI), to be compared with a denominator analysis (below) [6]. We further received a detailed description of the incident and proposals to avoid future incidents.

For peer review only

For peer review only - http://bmjopen.bmj.com/site/about/guidelines.xhtml

We got the annual number of patient-to-physician contacts (PPC) per practice from the *Sentinella* administration, as well as morbidity data from a fortnight cross-sectional denominator analysis of all patients consulting a *Sentinella* practice during weeks 11 or 12 [6].

We received the anonymized list of participating physicians, their specialty, as well as the community size (Swiss Federal Statistical Office) and the linguistic region from the *Sentinella* administration. Information on Swiss medication sales in 2015 by ATC groups was derived from Interpharma Switzerland (Appendix F).

The questionnaires were completed either electronically or on a paper/pencil version, the former as online SurveyMonkey™ questionnaires, the latter as sealed envelopes sent from the *Sentinella* administration. We had three study questionnaires: a detailed incident questionnaire, an initial one, and a final one (Appendices A, B, and C). The initial questionnaire served to describe the physician's practices in terms of e.g. number of physicians, availability of electronic patient history, electronic drug-drug interaction control system, and drug distribution system (Appendix B). The final questionnaire investigated non-reporting and difficulties with coding the morbidity variables (Appendix C).

We estimated "incident relevance" from the items "disturbance" and "endangering" of the patients; if any of them was graded with "medium" or higher, the variable relevance was set to "more", otherwise it was set to "less". Because of question ambiguity, we set coding of the item care-dependency to "missing" for patients younger than 20 years of age. Evans' Index – a prognostic index – was calculated by simple addition of the number of chronic drug treatments with the number of chronic conditions [7].

The following free text variables were manually coded: relationship of the incident to the suspected medication, preventability of the incident, reactions to the incident, and proposals to avoid further incidents.

Statistical methods

Values are given as frequencies, mean ± SD or median [interquartile range (IQR)] (as denoted with (first quartile; forth quartile), depending on non-normal distribution or non-interval scaled data level. To assess the association of medication incidents with potential risk factors we used the SPSS-GENLINMIXED procedure. Clustering of patients was addressed by using a mixed binary logistic regression with the fixed factors of gender, year of birth, care-dependency, number of chronic drug treatments, number of chronic conditions, and TMI as well as the physicians' practice number as a random factor; if one item was missing, the whole record was excluded from the analysis. Numera-

For peer review only

tor was affiliation to the case group; denominator was all PPC during weeks 11 and 12. We used IBM SPSS 23.

Results

The Sentinella system

During the year 2015, 149 practices were enrolled to the *Sentinella* system. Of them, 144 practices were known to report regularly; their properties are listed in Table 1. The *Sentinella* physicians are representative for the overall Swiss physician population [5,6]. Drugs were auto-distributed by 42% of the study practices. Approx. half of the physicians had electronic and the other paper-based medical records. Systematic drug-drug interaction control systems were installed only by a minority (36.8%) of the physicians. During the year 2015 (which included unusually for calendar adaptation, 53 instead of 52 reporting weeks), the general practitioners (GPs) had 4,456±2,137 PPC; for the pediatricians (PEDs), these were 5,297±2,715 (mean±SD). In their initial study questionnaire, 11.1% of the reporting physicians admitted to be working part time (i.e. less than 30 h per week).

Study flow

During the year 2015, we received 216 incident notifications (Figure 1). In 11 cases, we did not receive the detailed notification form. Eight cases had to be removed from the database because they fulfilled the exclusion criterion (i.e. “adverse drug reactions *without* detectable error”), and one case had to be removed because of double data entry. This led to 197 cases which could be analyzed. The distribution of the monthly incident notifications throughout 2015 is depicted in Figure e1 (Appendix D), and the distribution of the numbers of cases reported by each practice in Figure 2.

Description of patients

Table 2 lists age, gender and geographic distribution as well as the physician-to-patient relationship and the observer of the incident. Only three cases evolved in PED practices. No statistically significant differences were found between the two relevance classes of the incidents.

Number of incidents, non-reporting

In the GPs 194 incidents, 148 physicians, 4,456 yearly PPC led to 1.31 incidents per physician per year or 29.4 per 100,000 PPC; in PEDs 3 incidents, 32 physicians, 5,297 yearly PPC led to 0.1 incident per physician per year or 1.8 per 100,000 PPC.

To evaluate the *non-reporting of incidents*, we asked the physicians in the final study questionnaire. Out of 180 actively reporting physicians we received 145 questionnaires (80.6% response

For peer review only

rate). To our question: “Did you **not** report medication incidents that you had noticed during the last year?”, they answered: “Never or almost” 110 (75.9%), “yes, but seldom” 22 (15.2%), “yes, frequently” 9 (6.2%), “always, or almost” 4 (2.8%). Reasons for not reporting incidents were “lack of time” or “forgetfulness”. If we speculate that these answers represent reporting rates of 76-100, 51-75, 26-50, or 0-25% respectively, we could divide the observed rates by the middle of the reporting classes: 0.875, 0.625, 0.375 and 0.125. By doing so, we calculated a rate of 50% of underreporting; if we furthermore consider the 5% of the incidents where the questionnaires were not sent, this rate increases to 58%. We therefore have to multiply the observed rates with a factor of 1.58 resulting in the following rates of detected incidents: GP 2.07 per physician per year, 46.5 per 100,000 PPC and PED 0.15 per physician per year, 2.8 per 100,000 PPC.

Types and causes of incidents, organ systems involved, preventability

The *types of error* are listed in Table e1 (Appendix D) and Figure 3; in 26 cases more than one was mentioned. Most cases were linked to an incorrect dosage for a given patient, while prescription of an erroneous medication was the second most common error.

Most errors concerned orally ingested medication. *Parenteral drugs* led to fewer error reporting; in our study, they comprised insulin and vaccinations. There were cases of an incorrect (influenza vs. anti-tetanus) or incomplete (Boostrix® vs. Boostrix-Polio®) vaccination and of undue vaccination (a third anti-HPV vaccine). The first case was due to a communication problem within the practice staff, the other to a vaccination in absence of the vaccination card.

Three cases concerned pediatric patients, all were linked to inadequate dosing, two times of an antibiotic prescription (Cotrimoxazole, Co-Amoxicillin) and one time of an antiemetic drug (Thiethylperazine).

In 89 of the 195 cases, *organ system* damage was reported, in 13 cases more than one organ system was involved, most frequently the central nervous system (Figure 4) (Table e2, Appendix D). Possible *triggers* of the incident were reported in 194 of the 197 cases; in 45 cases there was more than one single reason, most frequently lacking alertness of the reporting physicians or their staff (Table e3). When asked who might be the “*responsible*” for the incident, 191 replied. The most common person or institution possibly responsible for the medication incident was: the reporting physician 41 (21.5%), followed by the practice nurse 26 (13.6%), the institution where the patient lives 33 (17.3%), the pharmacy 7 (3.7%), the hospital 12 (6.3%), the community nurse 4 (2.1%), the patients or their proxies 15 (7.9%), and the manufacturer 2 (1.0%); in 9 cases (4.6%) this was unclear, in 37 cases (19.4%), there was more than source one to blame. *Preventability* of the inci-

For peer review only - http://bmjopen.bmj.com/site/about/guidelines.xhtml

dents was classified (by our study board): unlikely in 6 cases (3.0%), possible in 58 (29.4%), probable in 114 (57.9%), and definite in 19 (9.6%).

Endangering and disturbances

In 192 of the 197 cases we received information about patients' *endangering* as estimated by the physicians. In 39 cases (20.3%) there was no endangering, in 83 (43.2%) light, in 51 (26.6%) moderate, in 19 (9.9%) severe. The *disturbances* caused by the incident are listed in Table 3. Out of the 197 incidents, 74 (37.5%) were classified as "more" relevant (Table 2).

Interface problems, orientation, predictability, repeat incidents

The presence of *interface problems* was reported in 64 of 197 cases (32.4%); these were: with a hospital in 28 cases (43.8%), with an institution in 14 (21.9%), with a community nurse in 6 (9.4%), with a pharmacist in 9 (14.0%), with a specialist in 3 (4.7%), and with others in 4 (6.3%). In 184 cases, we received information about *orienting* patients about the incident; in 98 cases, the patient was oriented by the reporting physician or his staff (50.3%), non-orientation was stated: because the patient was not able to understand (children, demented) in 26 cases (14.1%), because the problem had already been solved and the notification would have unduly disturbed the confidence in 18 cases (9.8%), because the patient or the proxies had observed themselves in 23 cases (12.5%), because the patient had already been oriented by others in 7 cases (3.8%) or because of another reason in 12 cases (6.5%). As for *predictability* of the incident, we received 183 valid answers; of them 82 (44.8%) stated "yes, in the given constellation, the incident was to be expected". When asked whether they had already reported a *similar incident* in the study, 29 out of 196 (14.8%) answered in the affirmative.

Risk factors to undergo a medication incident

To detect patient *risk factors* to undergo an incident, we compared the incident data with those of a fortnight denominator analysis [6]; the following univariate factors accumulated preferentially in incident patients: higher age, care-dependency, higher numbers of chronic conditions or medications (or higher Evans' Index), as well as higher TMI (table 4). In the multivariate analysis only inpatient care by an institution remained a significant factor. Other suspected risk factors are listed in Table e4 (Appendix D); these items were not included in the denominator study and therefore lack a comparator. Within the patient group with a more as compared to a less relevant incident, only psychiatric illness reached a significantly increased proportion. We did not detect major differences between the two drug distribution systems.

For peer review only - http://bmjopen.bmj.com/site/about/guidelines.xhtml

Relationship between incidents and drug class, ATC-group

We assessed semi-quantitatively whether the class of the suspected drug was causally linked to the emergence of the incident; the frequencies were: none 49 (25.1%), unlikely 31 (15.9%), possible 77 (39.5%), probable 34 (17.4%), definite 4 (2.1%), did not apply 2 (n.a). ATC codes of the suspected medications are listed in Table 5. Most naming concerned the groups C “cardiovascular” (23.2%) and N “nervous system” (22.1%). Among the medication classes judged to be *related* to the incident (n=37) the most frequently named group were oral anticoagulants: rivaroxaban 9, phenprocoumon 7, and acenocoumarol 2 cases; this explains the 7-fold increased relative risk of ATC group B as compared to sales. As an example for errors *without* relation to the drug class we must mention the 17 cases of institutionalized patients ingesting medications scheduled for other residents, in most cases this was person eating at the same table, but in other cases the person in care mixed up patient names.

Reactions to the incident and suggestions to prevent further ones

In 141 of the 197 cases, the physicians reported to have changed something after the incident, in 13 cases this was more than one *reaction*, mostly often named were communication with other caregivers or better instruction of patients (Table e5, Appendix D). The respondents were asked for proposals how to *prevent* future incidents of the reported type; 125 of them made a total of 243 suggestions (Table e6, Appendix D). Of these, 37 (15.2%) were related to accurate medication lists, 10 (4.1%) to better patient instructions, 38 (15.6%) to organization of regular follow-up controls, and 55 (22.6%) involved organizational changes within the practice and its staff.

Discussion

In a representative group of primary care physicians [8], we found approximately one case of a medication incident per GP and year.

Incident rates

We calculated the rates of detected medication incidents as follows: GP 2.07 per physician and year, 46.5 per 100,000 PPC and PED 0.15 per physician and year, 2.8 per 100,000 PPC. Medication incidents may make up a proportion of approximately one third of all incidents [9]; the rates for *all safety incidents* may amount 6.20 per physician and year or 139.4 per 100,000 PPC in GPs and 0.45 per physician and year or 8.4 per 100,000 PPC in PEDs.

In Australian primary care patients, an incident rate of 4.98 per GP and year was reported; this is close to our estimated rate of 6.20; however, there was no sub-typing of the incidents [10]. In a three-year study, O’Beirne and colleagues reported a rate of 1.8 safety incidents per year and phy-

For peer review only

sician [9]. In a literature review of western countries' publications, Sandars and Esmail described a rate of 5 to 80 errors per 100,000 consultations [2], a rate somewhat lower than the 139.4 cases per 100,000 consultations estimated in our study. Kuo and colleagues reported a proportion of 15% of all incidents to be related to medication [11]; since we estimated a proportion of 33% this would give rise to even higher rates of all safety incidents when calculated from our study database. A 11% group of the physicians worked part time, the rate per full time physician per year may therefore be somewhat higher than calculated. The 13-fold higher incident rate in GPs as compared to PEDs is not surprising given the lower medication rates in children. A recent British study confirmed this much lower rate of incidents in children; out of 46,902 family practice safety reports, 1,788 concerned children (26-times less than adults) [12].

Definition of incidents and reliability of reporting

On the other hand, incident rates may be influenced by their definition. Gandhi and colleagues coined the term "avoidable adverse drug reaction" [13]. In our study, we explicitly excluded adverse drug reactions *without* detectable error. Runciman and colleagues defined a patient safety incident as follows: "An event or a circumstance that could have resulted or did result in unnecessary harm to a patient" [14]. An intuitive definition of a medical error was given by Makeham et al: "That was a threat to patient wellbeing and should not happen. I don't want it to happen again." [15]. As shown in Figure e1 (Appendix D), reporting frequency was higher at the beginning of the study as compared to the later course of it. This could reflect some loss of interest or forgetfulness by the reporting physicians. As calculated from our final questionnaire after the study, the reporting physicians failed to report about one in three cases of the detected medication errors. Non-detection of incidents may even be more frequent than non-reporting of observed incidents. A missed possible drug-drug interaction may be detected by chart review, a documentation error would probably have been found only in 1:1 supervision, which is very time-consuming, costly and may additionally influence performance of the observed physician. It is therefore virtually impossible to decipher the real rate of non-detected incidents. The problems in detection of incidents were the reason to postulate a "mix of methods" as needed to identify adverse events in general practice [16].

Other approaches

In a retrospective, semi-quantitative analysis, Gehring and colleagues investigated safety incidents in Swiss primary care [17]; among 23 predefined classes of safety incidents, the respondents admitted 15 to have occurred at least yearly – four of them being linked to drug treatment. Another approach to incidents is to ask patients as performed by Mira and colleagues [18]; they interviewed patients (> 65 years with five or more chronic drug treatments) and found that 75% of the patients reported to have been affected by at least one medication error during the previous twelve months.

For peer review only - http://bmjopen.bmj.com/site/about/guidelines.xhtml

We did not collect data on appropriateness of treatment; admittedly medication inappropriate for some groups of patients (elderly or patients with impaired renal function) may provoke incidents [19]. A recent Scottish study demonstrated an impressive reduction in high-risk prescriptions (non-steroidal anti-inflammatory drugs, antiplatelet, and anticoagulation) as well as hospital admissions for gastrointestinal ulcers or heart failure by a combination of educational measures, informatics and financial incentives [20].

Type, consequences, causes and preventability of error

Most cases involved application of an erroneous dosing or of a wrong medication; non-application of necessary medication was also frequent. When a wrong medication was dispensed, the error was often caused by confounding of prepared medication in home residents. The classic case of non-application of a necessary medication was the missed re-uptake of anticoagulation after an operation. Non-application of necessary drugs seems to be a relevant source of unnecessary harm to patients [21]. The causing of the incidents was similar as reported by others [11,17,18,22]. Most studies found – as in our patients – lacking alertness of and communication problems within practice staff, but there was a large variety of other topics. All three pediatric cases were linked to prescription of an inadequate dosing for age and weight. More than half of the patients did not have any disturbances after the incident; otherwise in most cases the nervous system was affected. No fatalities were reported, but seven patients (3.5%) needed stationary care. In 2004 Pirohamed and colleagues published a study on adverse drug reactions as a cause for admission to hospital; they found that 6.5% of all hospitalizations and 4.0% of all hospital days were caused by adverse drug reactions, 72% of them being preventable and 2% leading to death [23], however, this study included all cases, and therefore a majority of cases of adverse drug reactions *without* a detectable error. An older Swiss study was published by Livio and colleagues; out of 3,195 hospitalizations, she identified 229 cases (7.2%) as probably caused by adverse drug reactions [24]. In that study, 32% of the events were classified as being preventable (which does not strictly mean that an error had caused the incident), and in 6% of the cases, fatalities were reported. The results on outpatients contrast to inpatients where one in ten drug administrations was described to be erroneous [25]. Medical errors seem to be the “third leading cause of death in the US” [26].

Risk factors

When looking for factors accumulating in the incident group, the univariate analysis comparing a fortnight denominator with our data revealed all factors investigated (except gender) as significantly accumulated in the incident group; these were higher age, care-dependency, higher numbers of chronic conditions or medications (and higher Evans’ Index), as well as higher TMI. When perform-

For peer review only - http://bmjopen.bmj.com/site/about/guidelines.xhtml

ing a multivariate analysis, only those living in a home for the elderly or handicapped remained a significant risk factor, but the small number of observations precluded possible less important risk factors to be detected. The only significant risk factor for undergoing an incident of higher *relevance* was psychiatric disease. In the literature there are several factors described to correlate with proneness to undergo a medication incident, which quite well reflects the results of our study. Mainly, this is evidently the number of drugs ingested [13,27]. Higher or young age [28], or morbidity [29,30] were also described. Most incidents concerned ATC-groups N “nervous system” or C “cardiovascular”, which were also of the mostly sold drugs; an exception was group B with anticoagulants and a sevenfold increased relative risk as compared to Swiss sales in 2015. It seems wise to be alert to avoid errors when prescribing medication of these groups. The prevailing position of anticoagulants (18.5% of cases) was described also by Field and colleagues [30]. Otherwise the repartition of our suspected drugs was similar to other primary care studies [11,30], a literature review [31] or a theoretical paper [22].

Limitations

There is likely – as expected – bias from selective and underreporting or non-detection of medication incidents. Therefore, the true rate of incidents could only be estimated, and the proportions of incident characteristics would probably substantially have been altered by a more complete recording of incidents.

Conclusion

Medication incidents are common in general medicine whereas they rarely occur in pediatrics. Reasons for medication incidents are diverse but often seem to be linked to communication problems. Older and multimorbid patients are at a particularly high risk for medication incidents.

Abbreviations

GP general practitioner

PED pediatrician

PPC patient-to-physician contacts

TMI Thurgau Morbidity Index

For peer review only

Author affiliations

MG Institute of Primary Care, University of Zurich, Switzerland.

AC Division of Clinical Pharmacology and Toxicology, Department of Internal Medicine, Ente Ospedaliero Cantonale, Lugano, and Department of Clinical Pharmacology and Toxicology, University Hospital Zurich, Zurich, and National Poisons Centre, Tox Info Suisse, Associated Institute of the University of Zurich, Zurich, Switzerland

DC Patientensicherheit Schweiz, Zurich, Switzerland.

LH Policlinique Médicale, University of Lausanne, Switzerland and Sentinel Surveillance Network, Swiss Federal Office of Public Health, Bern, Switzerland.

MP Epidemiology, Biostatistics, and Prevention Institute, University of Zurich, Switzerland.

AS Sentinel Surveillance Network, Swiss Federal Office of Public Health, Bern, Switzerland.

MZ Institute of General Medicine, University of Zurich, Switzerland.

Acknowledgements

We are grateful to Lee Wennerberg for the English language corrections, Dr Sven Staender, Männedorf for the helpful comments, and Simon Gnädinger, Zurich for the manual coding of free text items. We thank the *Sentinella* program commission for their support, the reporting physicians of *Sentinella* for their unflagging enthusiastic collection of data, and the Federal Office of Public Health for providing data and translating the questionnaires into French.

Contributorship statement

MG lead the study, did the pilot study (questionnaire development, data entering and processing) wrote all documents, did all the contacts with the *Sentinella* administration, ethics committee, and others, programmed the electronic questionnaires, entered hand-written questionnaires into the database, did the data processing and wrote the publication after data collection.

AC is an expert on clinical pharmacology and drug safety.

DC is an expert on patient safety.

LH is French-speaking and helped to interpret the French questionnaires. She is an expert on multimorbidity. She is a member of the *Sentinella* program commission.

MP is head of Epidemiology, Biostatistics & Prevention Institute. He is responsible for the sound methodology.

AS had the idea for the study. He is vice president of the *Sentinella* program commission.

MZ is expert on electronic data exchange in primary care.

For peer review only

All of them have seen all the study documents and have contributed intellectually to their elaboration. All have contributed to revise the draft of this publication and approve the submitted version of this publication.

Funding

The study was funded by Bangerter-Rhyner Foundation, Basel.

Competing interests

The authors declare that they have no financial interest conflicts with this study.

Patient consent

Not necessary for anonymous data.

Ethics approval

The ethical committee of Canton Zurich decided that our study did not need formal approval because the data are completely anonymous (KEK-ZH 2014-0400). The study was recorded in www.ClinicalTrials.gov: NCT02295371, as well as in our national study registry (www.kofam.ch; SNCTP000001207).

Data sharing statement

No additional data are available.

Strobe statement

Whenever possible, the guidelines of the Strobe statement were followed (Appendix E).

References

1. **Manias E:** Detection of medication-related problems in hospital practice: a review. *Br J Clin Pharmaco*l 2012; 76 (1): 7-20.
2. **Sandars J, Esmail A:** The frequency and nature of medical error in primary care: understanding the diversity across studies. Fam Pract 20 (3): 231-6, 2003.
3. **Miller GC, Britth HC, Valenti L.** Adverse drug events in general practice patients in Australia. *Med J Aust.* 2006;184(7):321-324.

For peer review only - http://bmjopen.bmj.com/site/about/guidelines.xhtml

4. **Thomsen LA, Winterstein AG, Søndergaard B, Haugbølle LS, Melander A.** Systematic review of the incidence and characteristics of preventable adverse drug events in ambulatory care. *Ann Pharmacother.* 2007; 41(9):1411-1426.

5. **Gnädinger M, Ceschi A, Conen D, et al:** Medication incidents in primary care medicine: protocol of a study by the Swiss Federal Sentinel Reporting System. BMJ Open 04/2015; 5(4): e007773. DOI:10.1136/bmjopen-2015-007773.

6. **Gnädinger M, Herzig L, Ceschi A et al:** The Burden Related to Chronic Conditions and Multimorbidity in the Swiss Primary Care Population: A Prospective Study in the Swiss Sentinel Surveillance Network (*Sentinella*), 2016, to be submitted. **That manuscript is included in the present submission.**

7. **Evans DC, Cook CH, Christy JM et al:** Comorbidtiy-polypharmacy scoring facilitates outcome prediction in older trauma patients. J Am Geriatr Soc 60: 1465-70, 2012.

8. **Cohidon C, Cornuz C, Senn N:** Primary care in Switzerland: evolution of physicians' profile and activities in twenty years (1993-2012). BMC Fam Pract 16: 107, 2015.

9. **Makeham M, Dovey S, Runciman W, et al:** Methods and Measures used in Primary Care Patient Safety Research. Results of a literature review. WHO report on patient safety Qual Safety Health Care 2008.

10. **Makeham MAB, Kidd MR, Saltman DC et al:** The threats to Australian patient safety (TAPS) study: incidents of reported errors in general practice. Med J Aus 185: 95-98, 2006.

11. **Kuo GM, Phillips RL Graham D et al:** Medication errors reported by US family physicians and their office staff. Qual Saf Health Care 17: 286-90, 2008.

12. **Rees P, Edwards A, Panesar S et al:** Safety incidents in the primary care office setting. Pediatrics 135 (6): 1027-35, 2015.

13. **Gandhi TK, Weingart SN, Borus BA, et al:** Adverse drug events in ambulatory care. N Engl J Med 348: 1556-64, 2003.

14. **Runciman W, Hibbert P, Thomoson R et al:** Towards an international classification ofr patient safety: key concepts and terms. Int J Qual Health Care 21 (1): 18-26, 2009.

15. **Makeham MAB, Dovey SM, County M et al:** An international taxonomy for errors in general practice: a pilot study. Med J Aus 177:68-72; 2002.

16. **Wetzels R, Wolters R, van Weel C et al:** Mix of methods is needed to identify adverse events in general practice: a prospective observational study. BMC Fam Pract 2008; 9:35.

For peer review only - http://bmjopen.bmj.com/site/about/guidelines.xhtml

17. **Gehring K, Schwappach DLB, Battaglia M et al:** Frequency of and harm associated with primary care safety incidents. Am J Managed Care 18 (9): e323-7, 2012.

18. **Mira JJ, Orozco-Beltran D, Perez-Jover V et al:** Physician patient communication failure facilitates medication errors in older polymedicated patients with multiple comorbidities. Fam Pract 30: 56-63, 2013.

19. **Cooper JA, Moriarty F, Ryan C et al:** Potentially inappropriate prescribing in two populations with differing socio-economic profiles: a cross-sectional database study using the PROMPT criteria. Eur J Clin Pharmacol 72: 583-91, 2016.

20. **Dreischulte T, Donnan P, Grant A et al:** Safer prescribing – a trial of education, informatics, and financial incentives. New Engl J Med 374: 1053-64, 2016.

21. **O'Grady I, Gerrett D:** Minimising harm from missed drug doses. Nursing Times 111 (44): 12-15, 2015.

22. **Huges RG, Ortiz E:** Medication errors. Why they happen, and how they can be prevented. Am J Nursing 205 (3): 14-24, 2005.

23. **Pirmohamed M, James S, Meakin S, et al:** Adverse drug reactions as cause of admission to hospital: prospective analysis of 18 820 patients. BMJ 2004; 329:15–19.

24. **Livio F, Buclin T, Yersin B et al:** Hospitalisations pour effet indésirable médicamenteux. Recensement prospectif dans un service d'urgences médicales [French]. Raisons de Santé 23, 1998.

25. **Berdot S, Gillaizeau F, Caruba T et al:** Drug administration errors in hospital inpatients: a systematic review. PLoS ONE 8 (6): e68856, 2013; doi: 10.1371/journal.pone.0068856.

26. **Makary M, Daniel M :** Medical error – the third leading cause of death in the US. BMJ 2016 ; 353: i2139.

27. **Nobili A, Pasina L, Tettamanti M et al:** Potentially severe drug interactions in elderly outpatients: results of an observational study of an administrative prescription database. J Clin Pharm Ther 34: 377-86, 2009.

28. **Avery AJ, Ghaleb M, Barber N, et al:** The prevalence and nature of prescribing and monitoring errors in English general practice: a retrospective case note review. Br. J Gen Pract e543, 2013.

29. **Field TS, Gurwitz JH, Avorn J et al:** Risk factors for adverse drug events among nursing home residents. Arch Intern Med 161: 1629-34, 2001.

30. **Field TS, Mazor KM, Briesacher B, et al:** Adverse drug events resulting from patient errors in older adults. J Am Geriatr Soc 55: 271-6, 2007.

For peer review only

31. **Saedder EA, Brock B, Nielsen LP et al:** Identifying high-risk medication: a systematic literature review. Eur J Clin Pharmacol 70: 637-45, 2014.

For peer review only

For peer review only - http://bmjopen.bmj.com/site/about/guidelines.xhtml

List of figures and tables

Figure 1: Study flow chart

Figure 2: Distribution of the number of cases reported by practice

Figure 3: Type of error

Figure 4: Organ system involved

Table 1: Characteristics of the reporting physicians in 2015

Table 2: General description of the cases

Table 3: Disturbances after the incident

Table 4: Possible risk factors for incident as compared to a denominator analysis during calendar weeks 11 and 12 [Gnädinger M 2016]

Table 5: ACT-Groups of suspected medications

For peer review only

Physicians' gender	
- male	128 (71.1%)
- female	52 (28.9%)
Physicians' age class	
- < 40	12 (6.7%)
- 40-49	44 (24.4%)
- 50-59	66 (36.7%)
- 60 and over	58 (32.2%)
Specialty	
- GP	148 (82.2%)
- PED	32 (17.8%)
Number of physicians in practice (n=144)	
- 1	69 (47.8%)
- 2	39 (27.1%)
- 3	17 (11.8%)
- 4 to 5	8 (5.6%)
- 6 to 9	7 (4.9%)
- 10 and over	4 (2.8%)
Number of physicians per practice reporting to *Sentinella* (n=144)	
- 1	119 (82.6%)
- 2	19 (13.2%)
- 3	5 (3.5%)
- 8	1 (0.7%)
Linguistic region	
- German	122 (67.8%)
- French	44 (24.4%)
- Italian	14 (7.8%)
Urbanity of the practice	
- urban	93 (51.7%)
- agglomeration	60 (33.3%)
- rural	27 (15.0%)
Workload per week	
- < 15 h	9 (5.0%)
- 15-30 h	36 (20.0%)
- > 30 h	135 (75.0%)
Drug distribution system	
- dispensing by physician	73 (42.2%)
- mixed system	19 (10.6%)
- dispensing by pharmacy	85 (47.2%)
Electronic documentation	
- yes	89 (49.4%)
- no	91 (50.6%)
Electronic interaction control	
- yes	65 (36.1%)
- no	115 (63.9%)
Electronic prescription	
- yes, with thesaurus	62 (34.4%)
- yes, but without thesaurus	24 (13.3%)
- none	94 (52.2%)
Certification of the practice	
- yes	46 (25.6%)
- none	134 (74.4%)
Staff meetings	
- yes, at least monthly	69 (38.3%)
- yes, but less frequently	70 (38.9%)
- none	41 (22.8%)
Quality circle participation	
- yes, at least monthly	134 (74.4%)

For peer review only

-	yes, but less frequently	23 (12.8%)
-	none	23 (12.8%)

Table 1. Characteristics of the reporting physicians in 2015 (144 practices, 180 physicians)
.

For peer review only

For peer review only - http://bmjopen.bmj.com/site/about/guidelines.xhtml

	Relevance		All
	less	more	
Number of cases	124	73	197
Patients age	69.2 ± 20.6	69.4 ± 21.2	69.3 ± 20.8
Patients gender,			
% males	40.3	32.9	37.6
Physicians specialty			
% pediatricians	1.6	1.4	1.5
Linguistic region, %			
- German	75.0	68.5	72.6
- French	18.5	28.8	22.3
- Italian	6.5	2.7	5.1
Physician-to-patient relationship, %			
- own family physician	86.3	78.1	83.2
- urgency / holiday replacing	0.8	4.1	2.0
- institution physician	11.3	17.8	13.7
- other	1.6	0.0	1.0
Observer of the incident; %			
- physician / practice staff	50.0	50.7	50.3
- patient / proxies	21.8	23.3	22.3
- community nurse	1.6	4.1	2.5
- institution (where patient lives)	15.3	16.4	15.7
- hospital	0.8	1.4	1.0
- other physicians	2.4	0.0	1.5
- pharmacist	7.3	4.1	6.1
- other	0.8	0.0	0.5

Table 2. General description of the cases. Differences between “less” and “more” categories are not statistically significant. We calculated the variable “incident relevance” from the variables “dis-

For peer review only

For peer review only - http://bmjopen.bmj.com/site/about/guidelines.xhtml

turbance” and “endangering” of the patients; if any of them was graded with “medium” or higher, the variable relevance was set to “more”, otherwise to “less”.

For peer review only

For peer review only - http://bmjopen.bmj.com/site/about/guidelines.xhtml

Item	N	percent
Severity of disturbance		
- no symptoms but pathological laboratory tests	15	8.0
- light	44	23.4
- moderate	22	11.7
- severe	10	5.3
- fatality	0	0.0
Subtotal (this is the base of the next rows)	*91*	*48.4*
- no symptoms, normal (or no) laboratory tests	97	51.6
Total	188	100.0
Missing data	9	n.a.
All patients	197	n.a.
Time until recovery		
- hours	26	28.5
- days	41	45.1
- weeks	15	16.5
- not yet known or missing information	9	9.9
All patients with disturbances	91	100.0
Recovering		
- without sequels	78	85.8
- with light to moderate sequels	2	2.2
- with severe sequels or fatality*	5	5.4
- not yet known or missing information	6	6.6
All patients with disturbances	91	100.0
Treatment / surveillance		
- not needed	48	52.7
- ambulatory care	33	36.3
- hospital care**	7	7.7
- missing information	3	3.3
All patients with disturbances	91	100.0

For peer review only

Table 3. Disturbances after the incident. * In one case, there was a reduced kidney function, the other cases remained unclear, and no fatalities were reported. ** Two cases had to be surveilled in the emergency room, the hospital stays were: intoxications with thiethylperazine, with fenoterol plus ipratropium bromide, with zolpidem, further a derailed diabetes type 2 (after missed treatment with metformin), and a gastro-intestinal hemorrhage in a patient where antithrombotic treatment with rivaroxaban was not communicated to the physician.

For peer review only

For peer review only - http://bmjopen.bmj.com/site/about/guidelines.xhtml

Item	Patient group		OR (95% CI) (crude)	OR (95% CI) (adjusted)
	Denominator	Incidents		
Number of observations	26,852	197	/	/
Age (mean±SD), years	46.7 ± 27.5	69.3 ± 20.8	1.004 (1.001;1.006)**[a]	1.001 (0.996;1.005)
Gender,				
● male	47.0	37.6	1	1
● female	53.0	62.4	1.048 (0.916;1.197)	1.055 (0.878;1.196)
Care-dependency, number (%)* [d]				
● none	16,335 (85.5%)	96 (51.6%)	1	1
● yes, by proxies	954 (5.0%)	12 (6.5%)	1.121 (0.789;1.594)	0.979 (0.674;1.423)
● yes, by community nurse	723 (3.8%)	22 (11.8%)	1.458 (1.025;2.073)[a]	1.201 (0.821;1.758)
● yes, by institution	1,099 (5.8%)	56 (30.1%)	1.802 (1.399;2.323)[c]	1.528 (1.141;2.046) [a]
Number of conditions (median, IQR)	2 (0;4)	5 (3;7)	1.052 (1.029;1.075)**[c]	1.030 (0.994;1.067)**
Number of chronic active treatments (median, IQR)	1 (0;4)	6 (3;9)	1.052 (1.030;1.074)**[c]	1.030 (0.995;1.067)**
Evans' Index (median, IQR)	3 (0;8)	11 (6;17)	1.009 (1.005;1.013)** [c]	n.a.***
Thurgau Morbidity Index value (%)[d]			[d]	
● 0	8,463 (31.5%)	24 (12.2%)	1	1
● 1	3,611 (13.4%)	8 (4.1%)	0.989 (0.787;1.242)	0.908 (0.694;1.190)
● 2	4,102 (15.3%)	23 (11.7%)	1.049 (0.847;1.300)	0.898 (0.685;1.169)
● 3	3,877 (14.4%)	39 (19.8%)	1.131 (0.914;1.399)	0.830 (0.611;1.127)
● 4	2,119 (7.9%)	39 (19.8%)	1.292 (1.004;1.662)[a]	0.901 (0.643;1.265)
● 5	1,539 (5.7%)	38 (19.3%)	1.420 (1.078;1.868)[b]	0.823 (0.547;1.239)
● 6	709 (2.6%)	26 (13.2%)	1.680 (1.178;2.396)[c]	0.866 (0.523;1.436)
missing values	18	0		

For peer review only - http://bmjopen.bmj.com/site/about/guidelines.xhtml

Table 4. Possible risk factors for incident as compared to a denominator analysis during calendar weeks 11 and 12 [Gnädinger M 2016]. * Because of question ambiguity this analysis was restricted to adult patients (age > 19 years); this led to 19,812 valid observations in the denominator and 183 in the incident groups. ** Per one conditions, medication, year or index point. *** Because Evans' Index is a composite of condition and medication numbers; it was not included in the multiple regression analysis. Significance levels: [a] $p<0.05$, [b] $p<0.01$, [c] $p<0.001$. [d] Because GENLIN procedure was not able to process ordinal scaled variables, correlations between study group and TMI or care-dependency were tested with Spearman's rho: the correlation coefficients were +0.075 or +0.094, respectively, $p<0.001$.

For peer review only

For peer review only - http://bmjopen.bmj.com/site/about/guidelines.xhtml

ATC-Class		All incidents	Incidents with *probable* or *definite* relationship with medication	Relative Risk (percentages left by right column)	Swiss 2015 sales* (number of packages)
A	Alimentary tract and metabolism	28 (14.6%)	6 (15.8%)	1.06	31,455,252 (14.9%)
B	Blood and blood forming organs	23 (12.0%)	7 (18.4%)	7.08	5,507,624 (2.6%)
C	Cardiovascular system	44 (22.9%)	1 (2.6%)	0.35	16,027,143 (7.5%)
D	Dermatologics	1 (0.5%)	0 (0.0%)	0.00	17,314,810 (8.2%)
G	Genito-urinary system and sex hormones	1 (0.5%)	0 (0.0%)	0.00	7,936,641 (3.7%)
H	Systemic hormonal preparations (excluding sex hormones and insulins)	7 (3.6%)	1 (2.6%)	2.00	2,875,760 (1.3%)
J	Anti-infectives for systemic use	23 (12.0%)	6 (15.8%)	3.95	8,444,623 (4.0%)
K	Infusion liquids	0 (0.0%)	0 (0.0%)	0.00	24,158,749 (11.5%)
L	Anti-neoplastic and immunomodulating agents	5 (2.6%)	1 (2.6%)	2.89	1,934,950 (0.9%)
M	Musculo-skeletal system	4 (2.1%)	2 (5.3%)	0.76	14,787,413 (7.0%)
N	Nervous system	43 (22.4%)	10 (26.3%)	1.30	42,690,195 (20.2%)
P	Anti-parasitic products, insecticides and repellents	1 (0.5%)	0 (0.0%)	0.00	462,559 (0.2%)
R	Respiratory system	7 (3.6%)	3 (7.9%)	0.57	28,837,468 (13.7%)
S	Sensory organs	2 (1.0%)	0 (0.0%)	0.00	7,658,311 (3.6%)
T	Diagnostic use	0 (0.0%)	0 (0.0%)	0.00	43,184 (0.0%)
V	Various	3 (1.6%)	1 (2.6%)	6.50	855,707 (0.4%)
	Total	192 (100.0%)	38 (100%)	1.0	210,990,389 (100.0%)

For peer review only

For peer review only - http://bmjopen.bmj.com/site/about/guidelines.xhtml

does not apply	5	0	n.a.	n.a.

Table 5. ACT-Groups of suspected medications. Relative risk of drugs with probable or definite relationship with the incident as compared to sales proportions. * Information by Interpharma Switzerland (Appendix F).

For peer review only

For peer review only - http://bmjopen.bmj.com/site/about/guidelines.xhtml

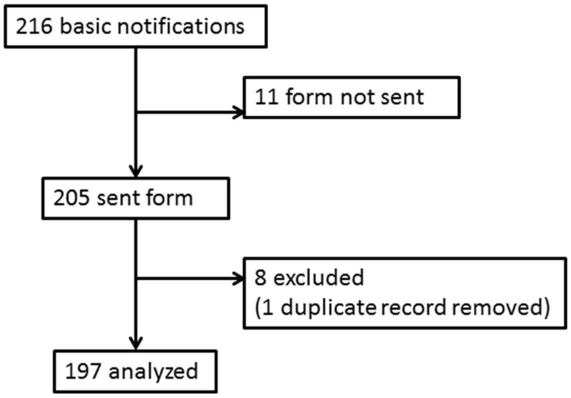

Study flow chart

254x190mm (96 x 96 DPI)

For peer review only - http://bmjopen.bmj.com/site/about/guidelines.xhtml

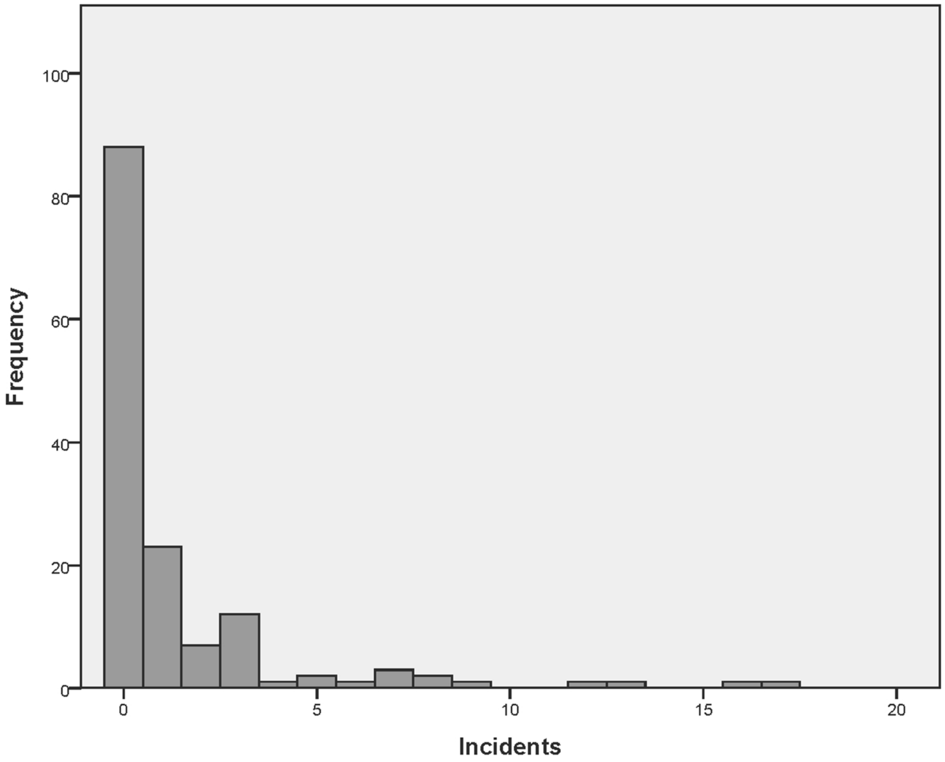

Distribution of the number of cases reported by practice

340x283mm (96 x 96 DPI)

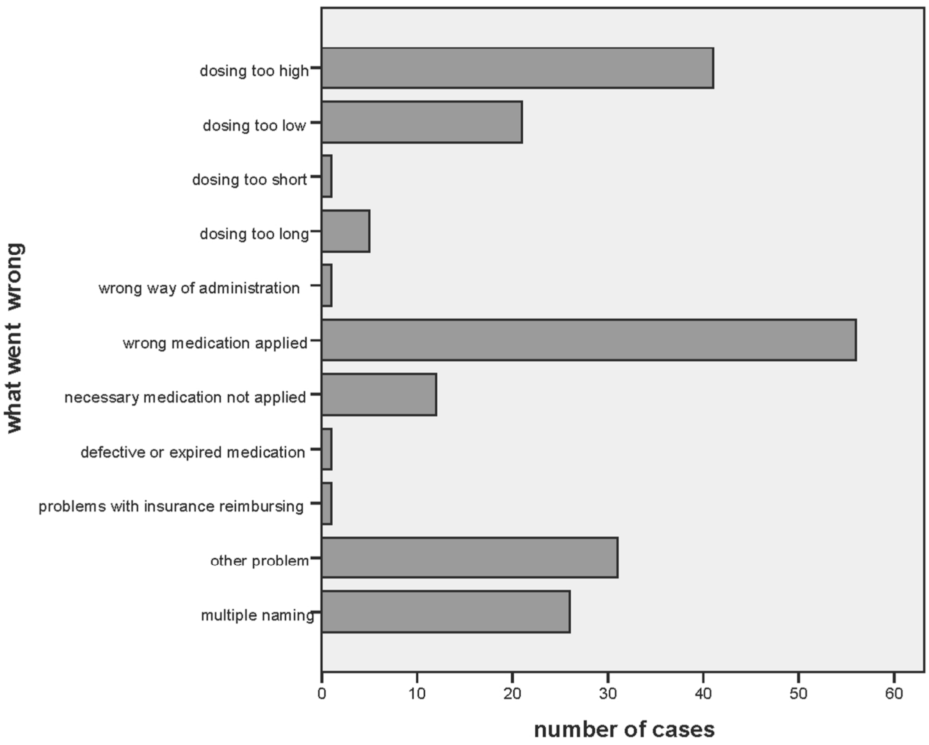

Type of error

348x288mm (96 x 96 DPI)

review only

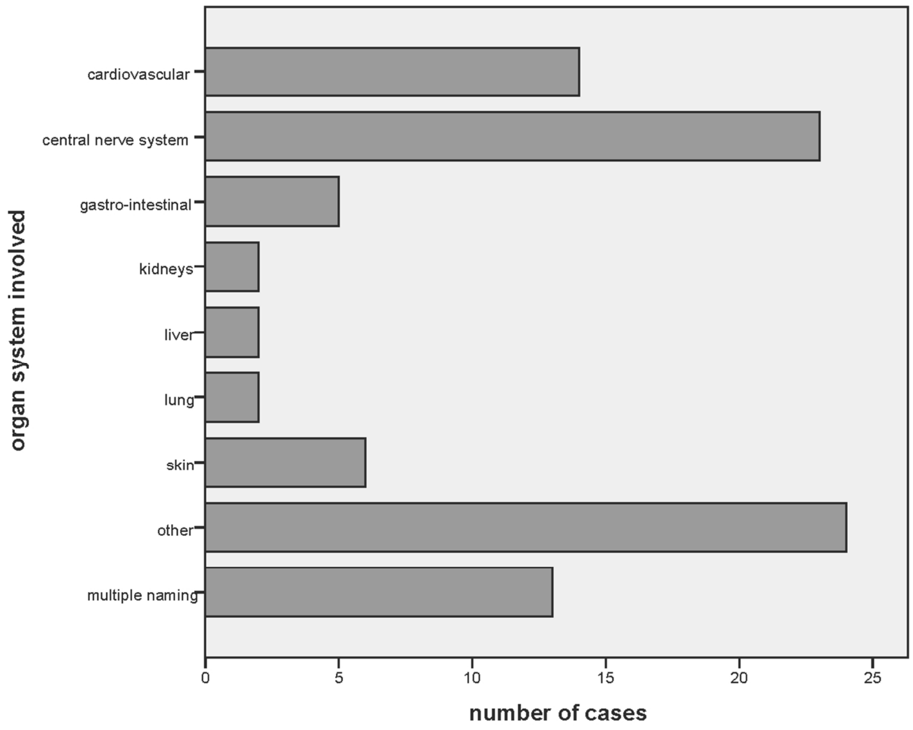

Organ system involved

356x290mm (96 x 96 DPI)

w only

Appendix A Incident questionnaire

Medication Incidents in Primary Care (MIPC)

(Version 1.0, January 25th 2015) (Internet version not available in English)

Incident reporting form

Administrative Information

1. Sentinella identification number: ☐☐☐☐ **2. Week of reporting:** ☐☐

The Patient

3. Year of birth: ☐☐☐☐ **4. Gender:** m ☐ f ☐

5. What was your relationship to the patient when the incident happened? Were you the

family physician ☐ emergency / substitute physician ☐ institution physician ☐ other ☐
if other what kind………………………

6. What is the patient's living situation?

with partner / family ☐ alone ☐ institution ☐ unknown ☐

7. Are there social problems?
yes ☐ no ☐ unknown ☐

8. Is the patient demented or otherwise mentally handicapped?

1

For peer review only - http://bmjopen.bmj.com/site/about/guidelines.xhtml

yes ☐ no ☐ unknown ☐

9. Does he suffer from psychological problems?

yes ☐ no ☐ unknown ☐

10. Does he take psychotropic drugs (esp. antidepressants, neuroleptics, benzodiazepines, opiates)?

yes ☐ no ☐ unknown ☐

11. Are there linguistic problems?

yes ☐ no ☐ unknown ☐

12. Does he or she smoke?

yes ☐ no ☐ unknown ☐

13. Is there substance abuse (other than nicotine)?

yes ☐ no ☐ unknown ☐**,** if yes, what substance? ……………………..

14. Does the patient have uncorrected / uncorrectable visual impairment?

yes ☐ no ☐ unknown ☐

15. Does the patient have uncorrected / uncorrectable hearing impairment?

yes ☐ no ☐ unknown ☐

16. Does the patient have uncorrected / uncorrectable mobility impairment?

yes ☐ no ☐ unknown ☐

17. Is there renal insufficiency (GFR: <60 ml/min/1.73 m^2)?

yes ☐ no ☐ unknown ☐

18. Is there hepatic insufficiency or liver cirrhosis?

yes ☐ no ☐ unknown ☐

19. Was the patient hospitalized in the past 12 months?
yes ☐ no ☐ unknown ☐

20. Is the patient taken care of by others? (*only one answer permitted*)?

yes, family / proxies ☐ yes, community nurse ☐ yes, institution ☐ no ☐ unknown ☐

21. Number of regularly applied active substances (including non-daily applied ones, see guidelines)?

☐☐ unknown ☐

22. Number of chronic conditions (see guidelines)?

☐☐ unknown ☐

23. Scale value of „Thurgau Morbidity Index” (chronic part, see guidelines)?

☐ unknown ☐

For peer review only - http://bmjopen.bmj.com/site/about/guidelines.xhtml

Details of the incident

24. Please, give a short **description** of the incident (in block letters):

25. Who observed the incident? (*multiple answers possible*)?

Physician / staff ☐ patient / relatives ☐ community nurse ☐ home / institution ☐ hospital ☐

other physicians ☐ pharmacist ☐ other ☐ unknown ☐

for „other" please specify: ……………………………

26. What happened (*multiple answers possible*)?

dosage too high ☐
dosage too low ☐
application too short ☐
application too long ☐
wrong administration route ☐
wrong medication ☐
indicated medication not received ☐
expired / defective medication ☐
problems with insurance reimbursement ☐

unclear / undefined ☐
other (please specify): ……………………… ☐

For peer review only - http://bmjopen.bmj.com/site/about/guidelines.xhtml

27. Please state the **trade name** of the medication used in the incident:

………………………………………………..

28. Please note other medication names, presuming they are relevant to the case.

none ☐ unknown ☐

29. How would you judge the degree of hazard to the patient during the incident?

mild ☐ medium ☐ severe ☐ none ☐ does not apply ☐ unknown ☐

30. How intense was the impairment (as judged by the patient)?

mild ☐ medium ☐ severe ☐ fatal ☐ no symptoms, but pathological lab values ☐
no impairment ☐ does not apply ☐ unknown ☐
If there wasn't *any* impairment, please skip to **question 34**.

31. How long did the impairment last?

hours ☐ days ☐ weeks ☐ longer ☐ unknown ☐

32. How was the recovery?
without residues ☐ with mild residues ☐ with severe residues / fatal ☐ unknown ☐

33. Which organ system was affected (*multiple answers possible*)?
cardiovascular ☐
central nervous ☐
gastro-enteral ☐
kidneys ☐
liver ☐
lung ☐
skin ☐
other, please specify ………………… ☐

For peer review only - http://bmjopen.bmj.com/site/about/guidelines.xhtml

34. Did you have to apply a specific surveillance or treatment for the incident?

yes, ambulatory ☐ yes, hospital ☐ no ☐ unknown ☐

If yes, please specify which? …………………………………………..

35. What factors contributed causally to the emergence of the incident (*multiple answers possible*)?

- off duty hours ☐
- communication failure within practice ☐
- generic substitution by pharmacist ☐
- hand-written prescription incorrectly interpreted ☐
- conflicting multiple prescriptions ☐
- lack of alertness within practice ☐
- lack of documentation ☐
- insufficient patient instruction ☐
- lack of aids (e.g. Dosette®) ☐
- lack of cooperation by patient / relatives ☐
- misleading package leaflet information ☐
- patient's internet search ☐
- administrative problems ☐
- manufacturer related (defective medication) ☐
- distributer related (out of stock) ☐
- lack of maintenance (e.g. first aid kit) ☐
- other, please specify……………………….. ☐
- unknown ☐

36. Was there an interface problem? If yes, which (*multiple answers possible*)?

- yes, with hospital ☐
- yes, with institution ☐
- yes, with community nurse ☐
- yes, with pharmacist ☐

For peer review only - http://bmjopen.bmj.com/site/about/guidelines.xhtml

yes, with specialist physician ☐
yes, with other, please specify? …………… ☐

no ☐

If **no**, please skip to **question 38**.

37. Was there an **explicit comparison** of prescription lists with the institution / person?

yes, verbal / by phone ☐ yes, written / by fax ☐ no ☐ unknown ☐

38/39. Was the patient informed about the incident?

yes, by myself / practice staff ☐
no, because he was not able to understand the message (children, demented) ☐
no, because the problem was solved and communication would have impaired confidence ☐
no, because the patient had moved or was deceased ☐
no, this was not needed because patient / relatives themselves had observed the incident ☐
no, because others had already informed him ☐

no, because: ………………………………………………………………………. ☐
unknown ☐

If yes, what was the patient's reaction? …………………………………………………

40. What did you do as a result to prevent similar incidents in the future (*multiple answers possible*)?

change standard operations procedures ☐
better instruction of patients ☐
communication with institution(s) ☐
notification of manufacturer ☐
notification of liability insurer ☐
notification of drug authority („yellow leaflet") ☐
notification of the "critical incident reporting system" ☐
other, please specify? ……………………….. ☐
nothing ☐

For peer review only - http://bmjopen.bmj.com/site/about/guidelines.xhtml

41. Who or what was ultimately **responsible** for the occurrence of the incident?
…………………………………………

42. In the given situation, do you think one could have **anticipated** the event?
yes ☐ no ☐

43. Did you already report an **identical** or very similar incident to this study?
yes ☐ no ☐

44. Please make any **suggestions** about the kind of measures that could be taken to generally reduce the frequency of such events (in block letters):

Please keep a copy of this questionnaire in the patient files. Thank you for filling it out!

For peer review only - http://bmjopen.bmj.com/site/about/guidelines.xhtml

Appendix B Initial questionnaire

Medication incidents in primary care (MIPC)

Initial reporting (Version 1.0, January 25th 2015)
Internet version: not available in English

1. Sentinella identification number: ………….

2. Number of physicians within your practice: ………….

3. Number of them who report to Sentinella ………….

4. Your weekly workload, h/w: <16 ☐, 16-30 ☐, >30 ☐

5. Number of hours within your practice (%): <50% ☐, ≥50% ☐

6. Approximate proportion of medication prescribed (as compared to directly delivered drugs):
<33% ☐, 33-66% ☐, >66% ☐

7. Do you have an X-ray (machine?)? yes ☐ no ☐

8. Do you have an ECG? yes ☐ no ☐

9. Do you have an ultrasound? yes ☐ no ☐

10. Do you have an electronic system for controlling electronic drug interaction?
yes ☐ no ☐

11. Do you have electronic patient history documentation?
yes ☐ no ☐

12. Do you prescribe electronically?
yes, with a medication thesaurus ☐ yes, but without one (use of a typewriter) ☐ no ☐

13. Is your practice certified (e.g. EQUAM)? yes ☐ no ☐

14. Do you regularly schedule team sessions?
Yes, at least monthly ☐, yes, but less frequently ☐, no ☐

For peer review only - http://bmjopen.bmj.com/site/about/guidelines.xhtml

15. Do you attend quality circle sessions (in accordance with "Hausärzte Schweiz")?
yes, regularly ☐, yes, now and then ☐, no ☐

16. Did you complete a special education (e.g. manual or psychosomatic medicine), or do you have special interests (e.g. toxic maniac patients)? yes ☐, no ☐
if yes, please specify : ……………………………………………………………………….

17. Are you contracted by an institution? yes ☐ no ☐
if yes, please specify (prison, home etc.): ……………………………………………………………

18. If yes, does this institution have specific problems with medication?
yes ☐, no ☐, if yes, please specify: …………………………………………………………

19. Are you involved in other special activities (teaching, research, insurance doctor)?
yes ☐ no ☐
If yes, please specify the kind of activity: …………………………………………………..

Thank you very much!

For peer review only - http://bmjopen.bmj.com/site/about/guidelines.xhtml

Appendix C Final questionnaire

Medication Incidents in Primary Care (MIPC)

Final questionnaire (Version 3.0 / December 14th 2015)

Online version: not available in English

Dear colleagues,

Thank you for your dedicated support of our study on medication incidents in 2015, regardless of whether you reported much of them or not. We would like to ask some final questions which will help us put the other information you sent us in the right context.

1. Sentinella identification number: ……………

2. Did you **not** report medication incidents that you had noticed during the last year (e.g. because of lack of time)?

never or almost never ☐, yes, but rarely ☐, yes, frequently ☐ always or almost always ☐

If this was frequently the case, please explain why:………………………………………..

3. Did your practice participate in the fortnight morbidity denominator study in March 2015 (calendar weeks 11 and 12)?
yes, fully ☐, yes, *but only partly (by omission of certain variables)* ☐, no ☐

If you did not fully participate or not at all, what was the reason?
………………………………………………………

If you did not participate in the denominator study, please continue with **question 12**.

4. How big was your effort for coding the morbidity variables of the denominator study?
manageable ☐, rather big ☐, too much ☐, impossible ☐

Did you have any difficulties when coding the morbidity variables?

5. Hospitalisation during the previous 12 months? none ☐, a little ☐, considerable ☐, severe ☐

6. Care-dependency? none ☐, a little ☐, considerable ☐, severe ☐

7. Number of medications? none ☐, a little ☐, considerable ☐, severe ☐

8. Number of conditions? none ☐, a little ☐, considerable ☐, severe ☐

9. Thurgau Morbidity Index? none ☐, a little ☐, considerable ☐, severe ☐

10. Repeat consultation during the fortnight? none ☐, a little ☐, considerable ☐, severe ☐

If you named considerable to severe difficulties in questions 5 to 10, please list them in **item 13**.

For peer review only - http://bmjopen.bmj.com/site/about/guidelines.xhtml

11. How long did it take for you and your practice nurse together for coding all of the variables of **one patient** (Dr. Gnädinger needed less than 3 minutes)?
………………………………………………. minutes

12. Would you be willing to be interviewed for a focus group on the subject of medication safety?

yes ☐, no ☐

13. Other comments:

Thank you very much!

For peer review only - http://bmjopen.bmj.com/site/about/guidelines.xhtml

Appendix D: electronic tables and figures

Figure e1: Distribution of the incident notifications over the year
Table e1: What went wrong with the incidents
Table e2: Organ system involved
Table e3: Causes of the incident
Table e4: Patient-sided possible risk factors
Table e5: Reactions to the incident
Table e6: Proposals to avoid further incidents

For peer review only - http://bmjopen.bmj.com/site/about/guidelines.xhtml

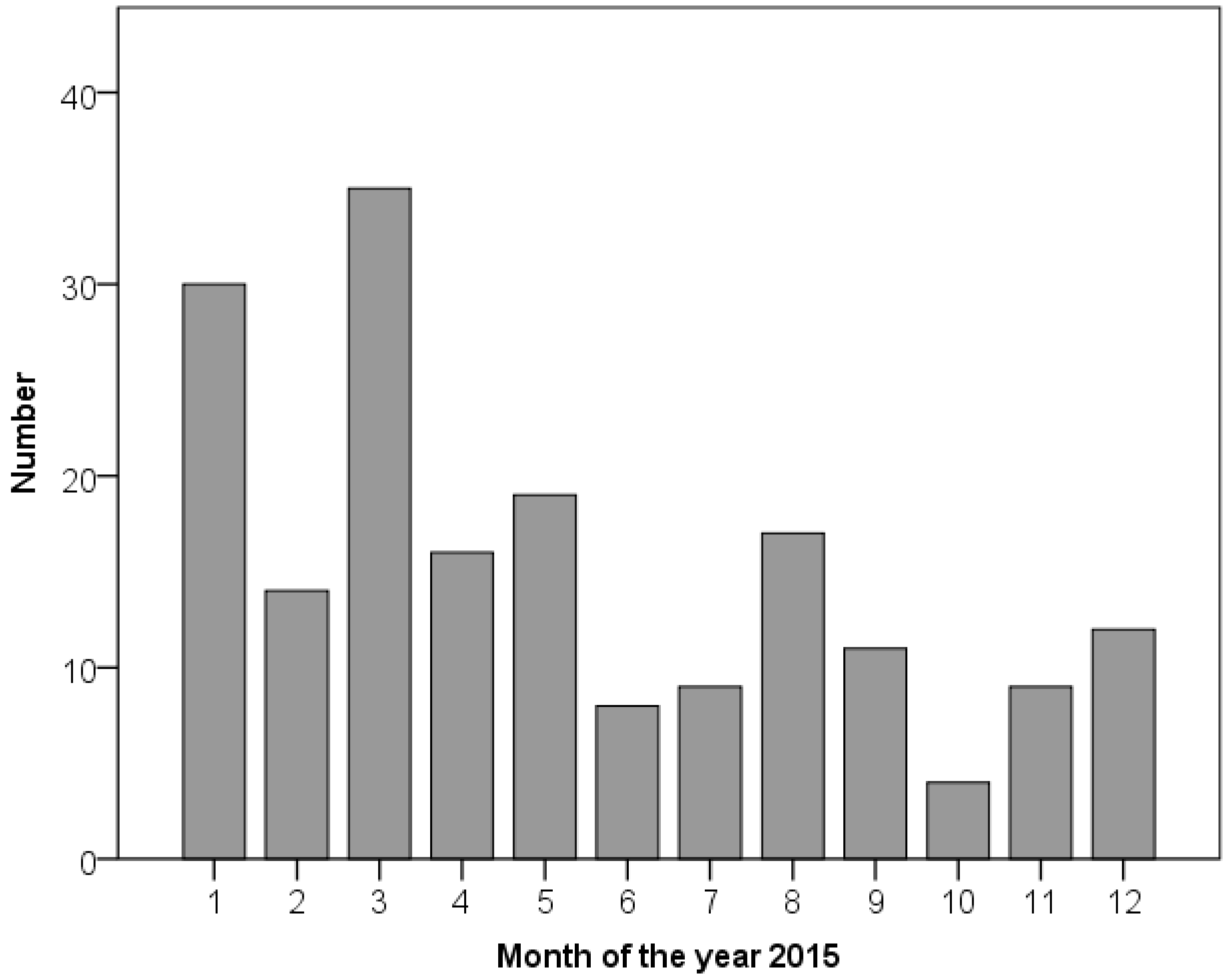

Figure e1. Distribution of the incident notifications over the year. Because only the week has been reported, the assignment to the month is approximate.

For peer review only - http://bmjopen.bmj.com/site/about/guidelines.xhtml

Type of error	N*	Percent*, %
Dosing too high	41 (12)	20.9 (6.1)
Dosing too low	21 (6)	10.7 (3.0)
Dosing too short	1 (2)	0.5 (1.0)
Dosing too long	5 (4)	2.6 (2.0)
Wrong way of administration	1 (1)	0.5 (0.5)
Wrong medication applied	56 (12)	28.6 (6.1)
Necessary medication not applied	12 (9)	6.1 (4.6)
Defective or expired medication applied	1 (1)	0.5 (0.5)
Problems with insurance reimbursing	1 (0)	0.5 (0.0)
Other problem**	31 (6)	15.8 (3.0)
Unknown	1 (0)	0.5 (0.0)
Multiple naming	26 (n.a.)	13.3 (n.a.)
Total	197 (53)	100.0 (26.9)

Table e1. What went wrong with the incidents. * Parenthesis denotes the additional naming within the category “multiple naming”. ** Naming within the category “other” were: confusion of similar trade names (2), dosing error (6), erroneous package size (1), confusion of similar looking preparations (1), error when controlling blood levels (2), double dosing (4), missed discontinuation of medication (1), necessary treatment not applied (1), wrong vaccine applied (4), missed re-uptake of anticoagulation after operation (1), contra-indication overlooked (2), drug-drug interaction overlooked (3), known intolerance overlooked (3), necessary monitoring missed (2), transgression of maximal drug allowance (2), delivery of incomplete package (1), confusion by contradictory medication lists (3), incorrect handling of administering device (1), uncontrolled taking of “nature medicines” (1), unreliable compliance with medication plan (2), two medications of the same drug

For peer review only - http://bmjopen.bmj.com/site/about/guidelines.xhtml

class (3), forbidden bisection of pills (2), confusion of similar named patients (1), intake of medication of the neighbor resident in a home (1), other (9).

For peer review only - http://bmjopen.bmj.com/site/about/guidelines.xhtml

Organ system	N*	Percent*, %
Cardiovascular	14 (10)	15.4 (11.0)
Central nervous system	23 (10)	25.3 (11.0)
Gastro-intestinal	5 (3)	5.5 (3.3)
Kidneys	2 (2)	2.2 (2.2)
Liver	2 (0)	2.2 (2.2)
Lung	2 (1)	2.2 (1.1)
Skin	6 (0)	6.6 (0.0)
Other**	24 (6)	26.4 (6.6)
Multiple naming	13 (n.a.)	14.3 (n.a.)
Total	91 (32)	100 (35.2)
Question does not apply	106	/

Table e2. Organ system involved. * Parenthesis denotes the additional naming within the category “multiple naming”. ** Naming within the category “other” were: endocrine system (9), musculo-skeletal (8), ear-nose-throat (2), psychic (1), other (9).

For peer review only - http://bmjopen.bmj.com/site/about/guidelines.xhtml

Causes of the incident	N*	Percent*;%
Out of hours	2 (3)	1.0 (1.5)
Communication problems within staff	10 (11)	5.1 (5.6)
Generic substitution of original trade medication by pharmacist	3 (3)	1.5 (1.5)
Difficulties when reading hand-written prescription	1 (1)	0.5 (0.5)
Multiple conflicting prescriptions	5 (8)	2.5 (4.1)
Lacking alertness of physician or practice staff	50 (17)	25.4 (8.6)
Insufficient documentation	3 (11)	1.5 (5.6)
Insufficient patient instruction	7 (17)	3.6 (8.6)
Lacking cooperation of patient / proxies	6 (17)	3.0 (8.6)
Confusion by reading package leaflet	1 (0)	0.5 (0.0)
Confusion after “Googleing”	1 (0)	0.5 (0.0)
Administrative problems	2 (3)	1.0 (1.5)
Defective medication as caused by manufacturer	3 (0)	1.5 (0.0)
Lacking maintenance (e.g. emergency case)	1 (0)	0.5 (0.0)
Lacking use of treatment aids (e.g. Dosett)	0 (6)	0.0 (3.0)
Other source of trouble**	54 (18)	27.2 (9.1)
Unknown	3 (0)	1.5 (0.0)
Multiple naming	45 (n.a.)	23.1 (n.a.)
Total	197 (115)	100.0 (58.4)

Table e3. Causes of the incident. * Parenthesis denotes the additional naming within the category “multiple naming”. ** Naming within the category “other” were: Erroneous delivery in pharmacy (5), treatment delayed/hampered by patient (2), transcription error (2), missed

For peer review only - http://bmjopen.bmj.com/site/about/guidelines.xhtml

discontinuation of an ongoing treatment (2), communication problem within helpers' network (16), similar trade names (2), interface problems with hospital (4), reading error of patient / proxies (3), erroneous execution of a medical prescription (14), missed follow-up control (9), difficult handling of a preparation (2), disregard of possible interactions (2), transgression of competence by care workers (5), incomplete information by patient / proxies (8), stress / lack of time (12), other 25.

For peer review only - http://bmjopen.bmj.com/site/about/guidelines.xhtml

Item (number of missing observations in groups "less" / "more")	Relevance		All
	less n (%)	more n (%)	n (%)
Number of patients	124 (100.0)	73 (100.0)	197 (100.0)
Living situation (1/0)			
- together with mate / family	61 (49.6)	31 (42.5)	92 (46.9)
- alone	28 (22.8)	23 (31.5)	51 (26.0)
- institution	33 (27.6)	19 (26.0)	53 (27.0)
Social problems (2/4)			
- yes	22 (18.1)	18 (26.1)	49 (20.9)
Dementia or mental illness (2/2)			
- yes	31 (25.4)	18 (25.4)	49 (25.4)
Psychiatric problems (1/2)			
- yes	28 (22.8)	28 (38.4)*	56 (28.9)
Treatment with psychotropic drugs (0/0)			
- yes	50 (40.3)	39 (53.4)	89 (44.2)
Linguistic problems (0/1)			
- yes	11 (8.9)	5 (6.8)	16 (8.2)
Smoking (7/4)			
- yes	11 (9.4)	8 (11.6)	19 (10.2)
Substance abuse other than nicotine (2/1)			
- yes	3 (2.5)	5 (6.9)	8 (4.1)
Visual blurring (6/6)			
- yes	4 (3.4)	4 (6.0)	8 (4.3)

For peer review only - http://bmjopen.bmj.com/site/about/guidelines.xhtml

Hearing problems (3/5)			
- yes	8 (6.6)	4 (5.9)	12 (6.3)
Gait problems (2/2)			
- yes	40 (32.8)	18 (25.4)	58 (30.1)
Renal insufficiency (GFR<60 ml/min*1.73m^2) (6/2)			
- yes	24 (20.3)	21 (29.6)	45 (23.8)
Liver cirrhosis of other hepatic function problem (3/1)	3 (2.5)	3 (4.2)	6 (3.1)
- yes			

Table e4. Patient-sided possible risk factors. * p=0.021 “more” vs. “less” by chi-square testing. We calculated the variable “incident relevance” from the variables “disturbance” and “endangering” of the patients; if any of them was graded with “medium” or higher, the variable relevance was set to “more”, otherwise to “less”.

Reactions to the incident	N*	Percent*; %
Changing standard operating procedures of the practice	14 (3)	7.2 (1.5)
Better instruction of patients	23 (4)	11.7 (2.0)
Communication with other institutions	31 (3)	15.7 (1.5)
Notifying manufacturer	1 (2)	0.5 (1.0)
Reporting the incident to the critical incident reporting system	10 (6)	5.1 (3.0)
No reaction at all	55 (n.a.)	27.9 (n.a)
Other type of reactions to the incident**	49 (9)	24.9 (4.6)
Missing information	1 (0)	0.5 (0.0)
Multiple naming	13 (n.a.)	6.6 (n.a.)
Total	197 (27)	100.0 (13.7)

Table e5. Reactions to the incident. * Parenthesis denotes the additional naming within the category “multiple naming”. ** Naming within the category “other” were: Hire more workforce (1), arrangements with other physicians (2), with pharmacist (4), with community nurse (4), with institution (12), with practice nurse (6), with specialist (1), with patient (3), with supplier (1), sending a new medication plan (1), having regular staff meetings (1), remove Digoxin 0.25 from assortment because of safety reasons (1), to not perform vaccinations in absence of vaccination card (2), to clarify intolerances (2), to clarify interactions (1), to hand out an allergy card (1), to actualize patient records (3), to apply for insurance cost credit (1), to organize follow-up controls (20), other (5).

For peer review only - http://bmjopen.bmj.com/site/about/guidelines.xhtml

Proposal	N*	Percent*;%
Cross-check of medication lists	6 (14)	4.8 (11.2)
Patient instructions	2 (3)	1.6 (2.4)
Reduction of time pressure / stress	3 (6)	2.4 (4.8)
To observe adverse drug reactions also with “nature products”	1 (0)	0.8 (0.0)
Four eyes check when dispensing medication	2 (4)	1.6 (3.2)
No medication without prescription	3 (6)	2.4 (4.8)
To critically audit polymedication	3 (2)	2.4 (1.6)
To avoid similarly looking or named medication in drug master	2 (3)	1.6 (2.4)
To demand medication plans to be brought to the consultation	0 (4)	0.0 (3.2)
To provide medication plans routinely	2 (8)	1.6 (6.4)
In-deep checking before delivering “new” medication to the patient	4 (1)	3.2 (0.8)
To provide patients with allergy / intolerance cards	2 (1)	1.6 (0.8)
To let patients themselves write their medication card (and controlling afterwards)	0 (3)	0.0 (2.4)
To instruct patients to come into the practice immediately after hospitalization	3 (0)	2.4 (0.0)
To broach regularly “medication safety” on staff meetings	5 (6)	4.0 (4.8)
To provide information in patients’ native language	0 (2)	0.0 (1.6)
To share important information about patients with practice nurse	0 (2)	0.0 (1.6)
To improve information flow	11 (16)	8.8 (10.4)
To organize follow-up controls	10 (28)	8.0 (22.4)
To wait for laboratory results before prescribing	1 (0)	0.8 (0.0)
Other**	12 (9)	9.6 (7.2)
Multiple answering	53 (n.a.)	42.4 (n.a.)

For peer review only - http://bmjopen.bmj.com/site/about/guidelines.xhtml

Total	125 (118)	100.0 (94.4)
None	72 (n.a.)	(n.a.)

Table e6. Proposals to avoid further incidents. * Parenthesis denotes the additional naming within the category “multiple naming”. ** Among naming within the category “other” were: timely involving of community nurse, stopping of self-medication in multimorbid or poly-medicated patients, to clearly labelling desensitization suspensions, to separating adult and pediatric vaccines, to implementing timely new guidelines, to refusing to be in care of a patient with bad compliance, to clearly separating and labelling of medication prepared for different patients, to advice patients for separating short- and long-acting insulins at their home, to providing short-time prescriptions with a clear expiration date, to allowing only adequately educated people to align and deliver medication in homes for the elderly and that delivering may be done by the same person who did the preparing of medication boxes, to avoiding similar looking medication boxes being muddled-up by patients at the refectory room.

For peer review only - http://bmjopen.bmj.com/site/about/guidelines.xhtml

Appendix E

STROBE Statement—Checklist of items that should be included in reports of ***cross-sectional studies***

	Item No	Recommendation
**Title and abstract**	1	(*a*) Indicate the study's design with a commonly used term in the title or the abstract **[yes]**
		(*b*) Provide in the abstract an informative and balanced summary of what was done and what was found **[yes]**
**Introduction**		
Background/rationale	2	Explain the scientific background and rationale for the investigation being reported **[yes]**
Objectives	3	State specific objectives, **[yes]** including any prespecified hypotheses **[no]**
**Methods**		
Study design	4	Present key elements of study design early in the paper **[yes]**
Setting	5	Describe the setting, locations, and relevant dates, including periods of recruitment, exposure, follow-up, and data collection **[yes]**
Participants	6	(*a*) Give the eligibility criteria, and the sources and methods of selection of participants **[yes]**
Variables	7	Clearly define all outcomes, exposures, predictors, potential confounders, and effect modifiers. **[yes]** Give diagnostic criteria, if applicable **[no]**
Data sources/ measurement	8*	For each variable of interest, give sources of data and details of methods of assessment (measurement). Describe comparability of assessment methods if there is more than one group **[yes]**
Bias	9	Describe any efforts to address potential sources of bias **[yes]**
Study size	10	Explain how the study size was arrived at **[yes]**
Quantitative variables	11	Explain how quantitative variables were handled in the analyses. If applicable, describe which groupings were chosen and why **[yes]**
Statistical methods	12	(*a*) Describe all statistical methods, including those used to control for confounding **[yes]**
		(*b*) Describe any methods used to examine subgroups and interactions **[yes]**
		(*c*) Explain how missing data were addressed **[yes]**
		(*d*) If applicable, describe analytical methods taking account of sampling strategy **[no]**
		(*e*) Describe any sensitivity analyses **[no]**
**Results**		

For peer review only - http://bmjopen.bmj.com/site/about/guidelines.xhtml

Participants	13*	(a) Report numbers of individuals at each stage of study—eg numbers potentially eligible, examined for eligibility, confirmed eligible, included in the study, completing follow-up, and analysed [no]
		(b) Give reasons for non-participation at each stage [yes]
		(c) Consider use of a flow diagram [yes]
Descriptive data	14*	(a) Give characteristics of study participants (eg demographic, clinical, social) and information on exposures and potential confounders [yes]
		(b) Indicate number of participants with missing data for each variable of interest [yes]
Outcome data	15*	Report numbers of outcome events or summary measures [yes]
Main results	16	(*a*) Give unadjusted estimates and, if applicable, confounder-adjusted estimates and their precision (eg, 95% confidence interval). Make clear which confounders were adjusted for and why they were included [yes]
		(*b*) Report category boundaries when continuous variables were categorized [yes]
		(*c*) If relevant, consider translating estimates of relative risk into absolute risk for a meaningful time period [no]
Other analyses	17	Report other analyses done—eg analyses of subgroups and interactions, and sensitivity analyses [yes]
**Discussion**		
Key results	18	Summarise key results with reference to study objectives [yes]
Limitations	19	Discuss limitations of the study, taking into account sources of potential bias or imprecision. Discuss both direction and magnitude of any potential bias [yes]
Interpretation	20	Give a cautious overall interpretation of results considering objectives, limitations, multiplicity of analyses, results from similar studies, and other relevant evidence [yes]
Generalisability	21	Discuss the generalisability (external validity) of the study results [yes]
**Other information**		
Funding	22	Give the source of funding and the role of the funders for the present study and, if applicable, for the original study on which the present article is based [yes]

*Give information separately for exposed and unexposed groups.

Note: An Explanation and Elaboration article discusses each checklist item and gives methodological background and published examples of transparent reporting. The STROBE checklist is best used in conjunction with this article (freely available on the Web sites of PLoS Medicine at http://www.plosmedicine.org/, Annals of Internal Medicine at http://www.annals.org/, and Epidemiology at http://www.epidem.com/). Information on the STROBE Initiative is available at www.strobe-statement.org

For peer review only - http://bmjopen.bmj.com/site/about/guidelines.xhtml

Appendix F

Swiss Pharmaceutical Market: Number of packaged sold by ATC-groups, 2015

	2015
	number of packa-ged sold
Total	210,992,389
A Alimentäres System und Stoffwechsel	31,455,252
A01 Stomatologika, Medizinische Präparate zur Mund- und Zahnpflege	1,473,520
A02 Antacida-Ulcustherapheutika, Antiflatulentia	6,051,251
A02A Antacida,Antiflatulentia	1,500,822
A02B Ulcustherapeutika	4,426,496
A02C Sonstige Magentherapeutika	123,933
A03 Produkte gegen funktionelle Magen-Darm-Störungen	2,087,299
A03A Antispasmodika+Anticholinergika rein	595,861
A03C Antispasmodika/Ataraktika-Kombinationen	25,411
A03E Antispasmodika, sonstige Kombinationen	222,102
A03F Gastroprokinetika	1,233,461
A03G Modulatoren der gastrointestinalen Sensomotorik	10,464
A04 Antiemetica und Antinausea	920,841
A05 Cholagoga und Leberschutz-Mittel	337,970
A05A Gallentherapeutica und Cholagoga	254,744
A05B Leberschutzpräparate	83,226
A06 Mittel gegen Verstopfung und Darmreinigung	4,583,118
A06A Mittel Gegen Verstopfung	4,329,037
A06B Darmreinigungsmittel	254,081
A07 Antidiarrhoica, Elektrolytzufuhr und intestinale Antiphlogistica	3,005,035
A07B Intestinale Adsorbierende Antidiarrhoica	228,076
A07E Intestinale Antiphlogistica	222,680
A07F Antidiarrhoica Micro-Organismen	1,340,432
A07G Orale Elektrolyt-Zufuhr	107,030
A07H Motilitätshemmer	1,095,216
A07X Übrige Antidiarrhoica inkl. A07A Antidiarrhoica intestinale Antiinfectiva	11,601
A08 Antiadiposita excl. Diätetica	51,654
A09 Digestiva und Enzyme	804,667
A10 Antidiabetica	3,376,580
A10C Humaninsulin und Analoga	1,078,495
A10H Sulfonylharnstoff Antidiabetika	321,219
A10J Biguanid Antidiabetika	1,217,578
A10K Glitazone Antidiabetika	25,305
A10M Glinide Antidiabetika	38,782
A10N DPP-IV Inhibitor Antidiabetika	483,559
A10P SGLT2-Hemmer Antidiabetika	45,927
A10S GLP-1 Agonisten Antidiabetika	156,656
A10X Übrige Antidiabetika inkl. Insulin tierischen Ursprungs und Alphaglukosidaseinhibitor Antidiabetika	8,616

1

A11 Vitamine	5,001,408
A11A Multivitamine mit Mineralstoffen	755,243
A11B Multivitamine ohne Mineralstoffe	17,507
A11C Vitamin A+D inkl. einfache Kombinationen	2,618,699
A11D Vitamin B1 und Kombinationen	170,884
A11E Vitamin B-Komplex	739,860
A11F Vitamin B 12 rein	123,445
A11G Vitamin C, inkl. Kombinationen mit Mineralstoffen	353,644
A11X Sonstige Vitamine	222,126
A12 Mineralverbindungen	3,490,891
A12A Mineralverbindung Calcium	1,254,334
A12B Mineralverbindung Kalium	200,438
A12C Sonstige Mineralverbindungen	2,036,119
A13 Tonica und Roborantia	229,735
A16 Übrige Präparate des alimentären und Stoffwechsel-Systems	34,295
B Blut und Blutbildende Organe	5,507,624
B01 Antithrombotika	4,009,465
B01A Vitamin-K-Antagonisten	399,963
B01B Heparine	714,872
B01C Thrombozytenaggregationshemmer	2,265,838
B01D Fibrinolytika	14,293
B01X Direkte Thrombin-Inhibit und direkte Faktor XA-Hemmber	598,766
B02 Blutgerinnungssystem – Sonstige Produkte	237,495
B02B Antagonisten (Antidot.Anticoag)	146,201
B02D Blutcoagulation	1,805
B02G Glitazone-Antidiabetika	48,969
B02X Übrige Blutgerinnungsprodukte (Tissues sealing preparations, Thrombopoietin Agonisten, Antifibrinolytica synthetisch etc.)	40,520
B03 Antianämica	1,259,896
B03A Antianämica mit Eisen und Kombinationen	862,744
B03C Erythropoietin-Präparate	67,258
B03X Sonstige Antianämica inkl. Folsäure/Folinsäure	329,894
C Herz-Kreislauf Therapie	16,027,143
C01 Cardica	954,962
C01B Anti-Arrhythmie-Präparate	213,952
C01C Cardiale Stimulantien excl. Herzglykoside	266,028
C01D Coronartherapeutica excl. Calcium-Antagonisten und Nitropräparate	121,815
C01E Nitropräparate und Analog	228,438
C01X Positive Inotrope, Herzglykoside und übrige Herzpräparate	124,729
C02 Antihypertonica	129,957
C03 Diuretica	1,815,738
C04 Cerebrale und periphere Vasotherapeutika	390,255
C05 Antivaricosa/Antihaemorrhoidalia	2,271,175
C05A Antihaemorrhoidalia topisch	773,800
C05B Antivaricosa topisch	1,029,010
C05C Antivaricosa systemisch	468,365
C06 Übrige Herz+Kreislaufmittel	339,930
C07 Betablocker	2,398,309
C07A Betablocker rein	2,272,988

For peer review only - http://bmjopen.bmj.com/site/about/guidelines.xhtml

C07B Betablocker Kombination	125,321
C08 Calciumantagonisten	1,157,267
C09 Stoffe mit Wirkung auf das Renin-Angiotensin-System	4,116,677
C09A Ace-Hemmer, rein	1,142,465
C09B Ace-Hemmer, Kombinationen	584,697
C09C Angiotensin-II-Antagonisten, rein	1,084,218
C09D Angiotensin-II-Antagonisten, Kombinationen	1,261,616
C09X Sonstige Stoffe mit Wirkung auf das Renin - Angiotensin - System	43,681
C10 Lipidregu/Antiarteriosklerotika	2,446,927
C10A Lipidregulatoren	2,341,963
C10A1 Statine (Cholesterolsynthesehemmer)	2,190,227
C10A2 Lipidsenker, Fibrate, Ionenaustauscher und sonstige Lipidsenker	151,736
C10C Lipidregulatoren kombiniert mit anderen Lipidregulatoren	104,964
C11 Übrige Präparate für die Herz-Kreislauf-Therapie	5,946
D Dermatologica	17,314,810
D01 Dermatologische Antimykotica	1,360,570
D02 Emollientia und Hautschutzmittel	2,703,273
D03 Wundheilmittel	4,389,791
D04 Antipruriginosa inkl. Antihistaminica und Anästhetica usw.	1,458,276
D05 Nichtsteroidale Präparate gegen entzündliche Hauterkrankungen	327,245
D06 Topische antibakterielle Produkte und antivirale Mittel	864,829
D06A Topische antibakterielle Produkte	379,013
D06D Topische Produkte gegen Virusinfektionen	485,816
D07 Corticosteroide topisch	1,982,173
D07A Reine Corticosteroide topisch	960,182
D07B Corticosteroid-Kombinationen topisch	1,021,991
D08 Antiseptica + Desinfizientia	1,836,788
D10 Aknemittel	1,227,850
D10A Topische Aknemittel	829,114
D10B Orale Aknemittel	398,736
D11 Übrige Dermatologica	1,164,015
G Urogenital-System+Sexualhormon	7,936,641
G01 Gynaekologische Antiinfektiva	812,985
G01A Trichomonadenmittel	185,395
G01B Gynaekologische Antimykotica	475,911
G01X Gynaekologische antibakterielle Produkte und Antiseptika	151,679
G02 Sonstige Gynaekologica	1,060,376
G02A Oxytocica	91,111
G02F Topische Sexualhormone	496,735
G02X Lokale Antikonzipientia, Prolactininhibitoren, wehenhemmende Mittel und sonstige Gynaekologika	472,530
G03 Sexualhormone und Stimulantien des Genitalsystems	3,917,793
G03A Hormonale Kontrazeptiva systemisch	2,477,919
G03B Androgene, excl.G3E,G3F	70,852
G03C Oestrogene, excl.G3A,G3E,G3F	397,050
G03D Progestogene, excl. G3A,G3F	245,149
G03F Oestrogen-Progestogen-Kombinationen	487,291
G03G Gonadotropin inl. sonstige Ovulationsstimulantien	100,879

For peer review only - http://bmjopen.bmj.com/site/about/guidelines.xhtml

G03X Androgen-Kombinationen mit weiblichen Sexualhormonen, SERMS und sonstige Sexualhormone und ähnliche Produkte	138,653
G04 Urologica	2,145,487
G04A Urologische Antiinfektiva und Harnantiseptika	410,545
G04C BPH Produkte (Benigne Prostata-Hyperplasie)	715,845
G04D Harninkontinenzprodukte	390,832
G04E Produkte gegen Erektionsstörungen	311,659
G04X Alle sonstigen Urologika	316,606
H Hormonales System, exkl. Sexualhormone	2,875,760
H01 Hypophysen-/Hypothalamus-Hormone	36,944
H02 Corticosteroide Systemisch	1,575,880
H03 Schilddruesentherapie	1,109,934
H04 Sonstige Hormone (wachstumshormone, antidiuretische Hormone, Anti-Parathyroid-Hormone etc.)	153,002
J Antiinfektiva systemisch	8,446,623
J01 System.Antibakter.Prod.	5,721,173
J01A Tetracycline und Kombinationen	155,659
J01C Breitspektrum Penicilline	2,353,136
J01D Cephalosporine	897,839
J01F Macrolide und vergleichbare Substanzen	778,785
J01G Fluorchinolone	817,056
J01H Mittel- und Schmalspektrum-Penicilline	111,484
J01K Aminoglykoside	46,447
J01P Sonstige Beta-Lactam. Antibiot. Penicilline, Cephalosporine (Monobactame, Peneme und Carbapeneme)	69,074
J01X Rifamycin und vergleichbare Substanzen, Trimethoprim, Kombinationen und vergleichbare Substanzen und sonstige antibakterielle Produkte	491,693
J02 Antimykotika, Systemisch	591,346
J04 Tuberkulosemittel	37,320
J05 Antivirale Mittel, systemisch	570,063
J05B Antivirale Mittel, exkl. Produkte gegen HIV	236,676
J05C Virustatika gegen HIV	333,387
J05C1 Nucleoside und Nucleotide Reverse Transkriptase Hemmer	108,107
J05C2 Protease Hemmer	98,581
J05CX Nicht-Nucleoside Reverse Transkriptase Hemmer, HIV Fusionshemmer, HIV-Antivirale Integrase Hemmer und sonst.HIV-Antivirale Mittel	126,699
J06 Sera und Gammaglobulin	23,164
J07 Vakzine	1,406,674
J07B Vakzine Kombinationen	463,631
J07B1 Kombinationen mit Tetanusimpfstoff	387,139
J07BX Mehrfach-Impfstoffe mit Masern und/oder Mumps und alle anderen Kombinationen	76,492
J07D Bakterielle Impfstoffe	164,542
J07E Virale Impfstoffe	687,366
J07E1 Influenza-Impfstoff	205,391
J07E4 Hepatitis-Impfstoff	212,503
J07E9 Alle uebrigen viralen Impfstoffe, inkl. HPV, Rotaviren, Varizellen etc.	269,472
J07X Alle uebrigen Impfstoffe und aehnliche Produkte	91,135
J08B Übrige Antiinfektiva	96,883
K Infusionslösungen	24,158,749
K01 Intravenoese Lösungen ab 100 ML	12,064,154

For peer review only - http://bmjopen.bmj.com/site/about/guidelines.xhtml

K01A Elektrolytlösungen	3,372,213
K01B Standardlösungen	8,461,628
K01D Fettemulsionen, inkl. Produkte zur totalen parenteralen Ernährung	156,484
K01E Aminosäuren-Lösungen	51,140
K01X Kalorische Lös. über 10% und Lösungen zur Osmotherapie	22,689
K02 Plasma Expander	70,547
K03 Blut- und Blutersatzinfusionslösungen	1,817
K04 Injektionslösungen/Infusionszusätze	12,005,621
K04A Elektrolytlösungen	752,499
K04B Standardlösungen	9,981,186
K04C Kalorische Lösungen < 100 ML	34,050
K04D Sonstige Injektionslösungen/Infusionszusätze < 100 ML	1,237,886
K05 Spüllösungen	16,610
L Antineoplastika und Immunmodulatoren	1,934,950
L01 Antineoplastika	984,072
L01A Alkylisierende Substanzen	46,698
L01B Antimetaboliten	323,741
L01C Vinca-Alkaloide & sonstige pflanzliche Produkte	157,793
L01D Antineoplastisch wirkende Antibiotika	46,619
L01F Platinhalt.Antineoplast.	95,347
L01G Monoklonale Antikörper zur antineoplastischen Therapie	159,208
L01H Proteinkinasehemmer zur antineoplastischen Therapie	33,895
L01X Sonstige Antineoplastika	120,771
L02 Cytostatische Hormonetherapie	183,379
L02A Cytostatische Hormone	43,050
L02B Cytostatische Hormonantagonisten	140,329
L03 Immunostimulantien	79,294
L03A Immunstimulantien	40,990
L03B Interferone	38,304
L04 Immunsuppressiva	688,205
L04B Anti-TNF-Produkte	340,857
L04X Sonstige Immunsuppressiva inkl. Interleukin Inhibitoren	347,348
M Muskel-und Skelettsystem	14,787,413
M01 Antirheumatica	6,373,707
M01A Corticoidfreie Antirheumatica	5,700,326
M01C Antirheumatica spezifisch	673,381
M02 Rheumaeinreibungen Rubefatientia	6,372,249
M03 Muskelrelaxantien	665,391
M03A Muskelrelaxantien peripher wirkend	85,757
M03B Muskelrelaxantien zentral wirkend	579,634
M04 Gichtmittel	368,020
M05 Übrigige Muskel-Skelettsystem-Präparate	1,008,046
N Nervensystem	42,690,195
N01 Anaesthetica	1,554,391
N01A Narkosemittel	1,078,794
N01A1 Narkosemittel Inhalationspräparate	20,582
N01A2 Narkosemittel Injektionspräparate	1,058,212
N01B Lokalanaesthetika	475,597

N01B1 Medizinische injizierbare Lokalanästhetika	198,964
N01B3 Topische Lokalanästhetika	270,962
N01B9 Sonstige Lokalanästhetika und dentale injizierbare Lokalanästhetika	5,671
N02 Analgetica	24,080,469
N02A Betäubungsmittel	1,661,607
N02B Analgetica und Antipyretica	21,918,098
N02B1 Analgetica rezeptpflichtig	8,592,792
N02B2 Analgetica rezeptfrei	13,325,306
N02C Migränemittel	500,764
N02C1 Triptane	432,741
N02C9 Sonstige Migränemittel	68,023
N03 Antiepileptica	1,957,745
N04 Parkinsonmittel	658,710
N05 Psycholeptica	9,075,955
N05A Antipsychotika	2,002,404
N05A1 Atypische Antipsychotika	1,521,618
N05A9 Konventionelle Antipsychotika	480,786
N05B Hypnotica und Sedativa	4,462,062
N05B1 Hypnotica und Sedativa, barbituratfreie Reinsubstanzen	2,830,382
N05B2 Hypnotica und Sedativa, barbituratfreie Kombinationen	129,461
N05B5 Pflanzliche Hypnotica und Sedativa	1,502,219
N05C Tranquilizer	2,611,489
N06 Psychoanaleptica Ex.Antiadipos	4,081,575
N06A Antidepressiva und stimmungsstabilisierende Produkte	3,503,509
N06A2 Pflanzliche Antidepressiva	245,372
N06A3 Stimmungsstabilisierende Produkte	50,396
N06A4 SSRI Antidepressiva	1,319,897
N06A5 SNRI Antidepressiva	664,070
N06A9 Sonstige Antidepressiva	1,223,774
N06B Psychostimulantia	341,172
N06X Psycholeptica-Psychoanaleptica-Kombinationen, Nootropica und Neurotonica sowie verschiedene andere Produkte	236,894
N07 Sonstige ZNS-wirksame Produkte	1,281,350
N07B Raucherentwöhnungsmittel	652,146
N07C Produkte gegen Schwindel	286,538
N07D Anti-Alzheimer Produkte	121,928
N07E Alkohol- und Opiatentwöhnungsmittel	126,973
N07X Alle anderen ZNS-wirksamen Präparate	93,765
P Parasitologie	462,559
P01 Antiprotozoenmittel und Anthelmintika	437,402
P01B Anthelmintica, excl. Schistosomiasis-Mittel	296,327
P01D Malariamittel	139,242
P02 Sonstige Antiparasitäre Präparate inkl. Insektizide und Repellentien	26,990
R Respirationssystem	28,837,468
R01 Rhinologica	7,246,454
R01A Rhinologica topisch	6,868,431
R01AX Corticosteroide Rhinologika	1,067,991
R01A6 Antiallergische Rhinologica	230,180
R01A7 Abschwellende Rhinologica	4,290,641

For peer review only - http://bmjopen.bmj.com/site/about/guidelines.xhtml

R01A9 Sonstige Rhinologica topisch inkl. Antiinfektive Rhinologika ohne Corticosteroide	1,279,619
R01B Rhinologica systemisch	378,023
R02 Halsschmerzmittel und Antiinfectiva	3,977,806
R03 Antiasthmatika und Produkte gegen chronisch-obstruktive Lungenerkrankungen	2,931,292
R03A B2-Stimulatoren	923,570
R03B Xanthine	16,188
R03C Degranulationshemmer	9,088
R03D Corticoide	258,075
R03F Komb. B2-Stimulatoren mit Corticoiden	1,172,896
R03G Anticholinergica rein und in Komb. mit B2-Stimulatoren	422,479
R03G3 Anticholinergica rein, Inhalate	269,418
R03G4 Anticholinergica – Kombinationen mit Beta-2-Stimulantien, Inhalate	153,061
R03J Antileukotrien Antiasthmatika	90,062
R03X Sonstige Antiasthmatika und Produkte gegen chronisch-obstruktive Lungenerkrankungen inkl. PDE4-Hemmer gegen Asthma / Chronisch-Obstruktive Lungenerkrankungen	38,934
R04 Percutane Einreibemittel und Inhalationspräparate	1,093,346
R05 Husten- und Erkältungspräparate	10,899,026
R05A Erkältungspräparate	2,498,594
R05C Expectorantia ohne Antiinfectiva	4,499,364
R05D Hustensedativa	3,297,144
R05D1 Hustenmittel plain (Einzelsubstanzen)	1,971,766
R05D2 Sonstige Hustensedativa, inkl. Kombinationen	1,325,378
R05F Sonstige Husten- und Erkältungspräparate	603,924
R06 Antihistamine systemisch	2,687,483
R07 Sonstige Präparate des Respirationssystems	2,061
S Sinnesorgane	7,658,311
S01 Ophthalmologica	7,214,097
S01A Ophtalmologische Antiinfectiva	451,604
S01B Ophtalmologische Corticoide rein	271,663
S01C Ophthalm. Antiphlogistika und Antiinfektiva Kombinationen	613,682
S01E Myotika und Mittel zur Glaukombehandlung	1,038,592
S01F Mydriatika und Cykloplegika	46,547
S01G Ophthalmologische Antiallergika, Abschwellende Mittel, Antiseptika	1,473,188
S01H Ophtalmolog. Lokalanesthetika	40,383
S01K Künstliche Tränen und Netzmittel für Augen	2,503,750
S01P Ophthalmologische Antineovaskularisationsprodukte	127,501
S01R Nonsteroidale entzündungshemmende Ophthalmologica (NSAID'S)	236,207
S01X Übrige Ophtalmologica inkl. Präparate für den Gebrauch von Kontaktlinsen, Augentonika und Augenvitamine, Präparate zur Verhütung von Katarakt und Antikataraktogenika, Ophthalmologische Operationshilfsmittel und Ophthalmologische Diagnostika	411,009
S02 Otologica	444,214
T Diagnostika	43,184
T01 Diagnostika für bildgebende Verfahren	18,885
T02 Testdiagnostika	24,299
V Verschiedenes	855,707
V01 Allergene	6,429
V03 Übrige Therapeutische Präparate	820,500

For peer review only - http://bmjopen.bmj.com/site/about/guidelines.xhtml

V03D Entgiftungspräparate für die Cytostatikatherapie	45,668
V03E Antidote	4,532
V03G Hyperkaliämie/Hyperphosphatämie-Produkte	42,259
V03X Sonstige therapeutische Präparate inkl. Entzündungshemmende Enzyme, Eisen-Chelatbildner und Radiopharmazeutika	728,041
V05 Chirurgische Antiseptica	134
V06 Allgemeine Nährmittel	5,612
V07 Alle übrigen nicht therapeutischen Präparate	23,032

Source: Interpharma with data from IMS Health Schweiz, 2016.

For peer review only - http://bmjopen.bmj.com/site/about/guidelines.xhtml

STROBE Statement—Checklist of items that should be included in reports of ***cross-sectional studies***

	Item No	Recommendation	Manu-script page
**Title and abstract**	1	(*a*) Indicate the study's design with a commonly used term in the title or the abstract [yes]	1
		(*b*) Provide in the abstract an informative and balanced summary of what was done and what was found [yes]	1-2
**Introduction**			
Background/rationale	2	Explain the scientific background and rationale for the investigation being reported [yes]	3
Objectives	3	State specific objectives, [yes] including any prespecified hypotheses [no]	3
**Methods**			
Study design	4	Present key elements of study design early in the paper [yes]	3
Setting	5	Describe the setting, locations, and relevant dates, including periods of recruitment, exposure, follow-up, and data collection [yes]	3
Participants	6	(*a*) Give the eligibility criteria, and the sources and methods of selection of participants [yes]	3
Variables	7	Clearly define all outcomes, exposures, predictors, potential confounders, and effect modifiers. [yes] Give diagnostic criteria, if applicable [no]	3
Data sources/ measurement	8*	For each variable of interest, give sources of data and details of methods of assessment (measurement). Describe comparability of assessment methods if there is more than one group [yes]	4
Bias	9	Describe any efforts to address potential sources of bias [yes]	4
Study size	10	Explain how the study size was arrived at [yes]	4-5
Quantitative variables	11	Explain how quantitative variables were handled in the analyses. If applicable, describe which groupings were chosen and why [yes]	4-5
Statistical methods	12	(*a*) Describe all statistical methods, including those used to control for confounding [yes]	5
		(*b*) Describe any methods used to examine subgroups and interactions [yes]	5
		(*c*) Explain how missing data were addressed [yes]	5
		(*d*) If applicable, describe analytical methods taking	n.a.

For peer review only

		account of sampling strategy [no]	
		(e) Describe any sensitivity analyses [no]	n.a.
**Results**			
Participants	13*	(a) Report numbers of individuals at each stage of study—eg numbers potentially eligible, examined for eligibility, confirmed eligible, included in the study, completing follow-up, and analysed [no]	5
		(b) Give reasons for non-participation at each stage [yes]	5
		(c) Consider use of a flow diagram [yes]	18
Descriptive data	14*	(a) Give characteristics of study participants (eg demographic, clinical, social) and information on exposures and potential confounders [yes]	22-24
		(b) Indicate number of participants with missing data for each variable of interest [yes]	5
Outcome data	15*	Report numbers of outcome events or summary measures [yes]	6
Main results	16	(*a*) Give unadjusted estimates and, if applicable, confounder-adjusted estimates and their precision (eg, 95% confidence interval). Make clear which confounders were adjusted for and why they were included [yes]	28
		(*b*) Report category boundaries when continuous variables were categorized n.a.	n.a.
		(*c*) If relevant, consider translating estimates of relative risk into absolute risk for a meaningful time period [no]	n.a.
Other analyses	17	Report other analyses done—eg analyses of subgroups and interactions, and sensitivity analyses [yes]	24
**Discussion**			
Key results	18	Summarise key results with reference to study objectives [yes]	8
Limitations	19	Discuss limitations of the study, taking into account sources of potential bias or imprecision. Discuss both direction and magnitude of any potential bias [yes]	2
Interpretation	20	Give a cautious overall interpretation of results considering objectives, limitations, multiplicity of analyses, results from similar studies, and other relevant evidence [yes]	8-11
Generalisability	21	Discuss the generalisability (external validity) of the study results [yes]	6
**Other information**			

For peer review only

Funding	22	Give the source of funding and the role of the funders for the present study and, if applicable, for the original study on which the present article is based [yes]	13

*Give information separately for exposed and unexposed groups.

Note: An Explanation and Elaboration article discusses each checklist item and gives methodological background and published examples of transparent reporting. The STROBE checklist is best used in conjunction with this article (freely available on the Web sites of PLoS Medicine at http://www.plosmedicine.org/, Annals of Internal Medicine at http://www.annals.org/, and Epidemiology at http://www.epidem.com/). Information on the STROBE Initiative is available at www.strobe-statement.org

For peer review only

BMJ Open

Medication Incidents in Primary Care Medicine: a Prospective Study in the Swiss Sentinel Surveillance Network (Sentinella)

Journal:	*BMJ Open*
Manuscript ID	bmjopen-2016-013658.R2
Article Type:	Research
Date Submitted by the Author:	27-Dec-2016
Complete List of Authors:	Gnädinger, Markus; University of Zurich, Institute for General Medicine<br>Conen, Dieter; Swiss Patient Safety,<br>Herzig, Lilli; University of Lausanne, Institute of General Medicine<br>Puhan, Milo; University of Zurich, Institute of Epidemiology, Biostatistics & Prevention<br>Staehelin, Alfred; Sentinel Surveillance Network, Swiss Federal Office of Public Health,<br>Zoller, Marco; Zurich University Hospital, Instiute for General Medicine<br>Ceschi, Alessandro; National Poisons Centre, Tox Info Suisse, Associated Institute of the University of Zurich, Division of Science, Head of ; University Hospital Zurich , Dept. Clinical Pharmacology & Toxicology
**Primary Subject Heading**:	General practice / Family practice
Secondary Subject Heading:	Pharmacology and therapeutics, Paediatrics, Medical management, Nursing
Keywords:	Health & safety < HEALTH SERVICES ADMINISTRATION & MANAGEMENT, Adverse events < THERAPEUTICS, Patient safety, Pharmaceutical preparations, Medication errors

SCHOLARONE™
Manuscripts

For peer review only

Medication Incidents in Primary Care Medicine

A Prospective Study in the Swiss Sentinel Surveillance Network (*Sentinella*)

[1]Markus Gnädinger (corresponding author, markus.gnaedinger@hin.ch), [2]Dieter Conen, [3,4]Lilli Herzig, [5]Milo A. Puhan, [1,4]Alfred Staehelin, [1]Marco Zoller, [6]Alessandro Ceschi

[1]Institute of Primary Care, University of Zurich, [2]Patientensicherheit Schweiz, Zurich [3]Policlinique Médicale, University of Lausanne, [4]Sentinel Surveillance Network, Swiss Federal Office of Public Health, Bern (*Sentinella*), [5]Epidemiology, Biostatistics, and Prevention Institute, University of Zurich, [6]Division of Clinical Pharmacology and Toxicology, Institute of Pharmacological Sciences of Southern Switzerland, Ente Ospedaliero Cantonale, Lugano, Switzerland and Department of Clinical Pharmacology and Toxicology, University Hospital Zurich, Zurich, Switzerland and National Poisons Centre, Tox Info Suisse, Associated Institute of the University of Zurich, Zurich, Switzerland

(**Correspondence:** Markus Gnädinger, Dr. med. Facharzt für Innere Medizin, Birkenweg 8, 9323 Steinach, 0041 71 446 04 64)

For peer review only

Abstract

Objectives: To describe the type, frequency, seasonal and regional distribution of medication incidents in primary care in Switzerland and to elucidate possible risk factors for medication incidents.

Design: Prospective surveillance study.

Setting: Swiss primary health care, Swiss Sentinel Surveillance Network.

Participants: Patients with drug treatment who experienced any *erroneous* event related to the medication process and interfering with normal treatment course, as judged by their physician. The 180 physicians in the study were general practitioners or pediatricians participating in the Swiss Federal Sentinel reporting system in 2015.

Outcomes: *Primary:* medication incidents; *secondary:* potential risk factors like age, gender, poly-medication, morbidity, care-dependency, previous hospitalization.

For peer review only - http://bmjopen.bmj.com/site/about/guidelines.xhtml

Results: The mean rates of detected medication incidents were 2.07 per general practitioner per year (46.5 per 100,000 contacts) and 0.15 per pediatrician per year (2.8 per 100,000 contacts), respectively. The following factors were associated with medication incidents (OR, 95% CI): higher age 1.004 per year (1.001;1.006), care by community nurse 1.458 (1.025;2.073) , and care by an institution 1.802 (1.399;2.323) , chronic conditions 1.052 (1.029;1.075) per condition, medications 1.052 (1.030;1.074) per medication, as well as Thurgau Morbidity Index for stage 4: 1.292 (1.004;1.662), 5: 1.420 (1.078;1.868), and 6: 1.680 (1.178;2.396), respectively. Most cases were linked to an incorrect dosage for a given patient, while prescription of an erroneous medication was the second most common error.

Conclusions: Medication incidents are common in adult primary care whereas they rarely occur in pediatrics. Older and multimorbid patients are at a particularly high risk for medication incidents. Reasons for medication incidents are diverse but often seem to be linked to communication problems.

Trial registration: www.clinicaltrials.gov, NCT0229537

Keywords: Patient safety, pharmaceutical preparations, medication errors.

Strength and limitations

This is the first prospective and systematic collection of incident data in primary care in Switzerland that is characterized by three linguistic regions and two drug distribution systems.

It was conducted by experienced physicians and with high response rates.

There is likely – as expected – bias from selective and underreporting or non-detection of medication incidents.

Introduction

Patient safety is a major concern in healthcare systems worldwide. Although most safety research has been conducted in the in-patient setting [1], evidence indicates that medical errors and adverse events pose a serious threat for patients in the primary care setting as well, since most patients receive ambulatory care [2-4]. Information about the frequency and outcomes of safety incidents in primary care is required to identify risks or 'hot spots', to prioritize them and to take action as needed [5]. The aim of the project was to describe the type, incidence, seasonal and regional

For peer review only - http://bmjopen.bmj.com/site/about/guidelines.xhtml

distribution of medication incidents in primary care in Switzerland and to elucidate risk factors for medication incidents.

Method

Study design

We conducted a prospective surveillance study among primary care patients during 2015 to identify cases of medication incidents.

Study population

The study population was any person undergoing drug treatment in general internal or pediatric practices participating the *Sentinella* network. The latter covers a representative sample of patients in primary care for Switzerland [6, *included to this manuscript*]. Founded in 1986, it was mainly designed to survey transmissible diseases. Later, it also assessed other health problems of public interest. It generates daily to weekly current data and covers the entire geographic and linguistic regions of our country. Children, the mentally handicapped or the elderly were also included, all of whom might be at increased risk for medication errors.

Medication incidents

We defined medication incidents as any erroneous event (as defined by the physician) related to the medication process and interfering with normal treatment course (e.g. administration of an erroneous medication). We did not include lack of treatment effect, adverse drug reactions or drug-drug or drug-disease interactions without detectable treatment error. Nor did we consider medication incidents if patients refused to have them reported to the *Sentinella* system.

Data sources

The study physicians recorded the patient's year of birth and gender on their weekly reporting form. After a maximum of four weeks they had to fill in a detailed incident questionnaire (Appendix A). It was comprised of their *Sentinella* number and the calendar week of notification. Concerning the patients, they reported the living situation, several supposed risk factors for an incident, as well as the following variables: hospitalization during the previous year, care-dependency, number of drugs used chronically, number of chronic conditions, and the Thurgau-Morbidity-Index (TMI, a seven step Likert scale to measure morbidity in outpatients), to be compared with a denominator

For peer review only - http://bmjopen.bmj.com/site/about/guidelines.xhtml

analysis (below) [6]. We further received a detailed description of the incident and proposals to avoid future incidents.

We got the annual number of patient-to-physician contacts (PPC) per practice from the *Sentinella* administration, as well as morbidity data from a fortnight cross-sectional denominator analysis of all patients consulting a *Sentinella* practice during weeks 11 or 12 [6].

We received the anonymized list of participating physicians, their specialty, as well as the community size (Swiss Federal Statistical Office) and the linguistic region from the *Sentinella* administration. Information on Swiss medication sales in 2015 by ATC (Anatomical Therapeutic Chemical Classification System, an international classification for pharmaceutical products) groups was derived from Interpharma Switzerland (Appendix F).

The questionnaires were completed either electronically or on a paper/pencil version, the former as online SurveyMonkey™ questionnaires, the latter as sealed envelopes sent from the *Sentinella* administration. We had three study questionnaires: a detailed incident questionnaire, an initial one, and a final one. The detailed incident questionnaire collected information about the circumstances of the incident and about the patient's properties (Appendix A). The initial questionnaire served to describe the physician's practices in terms of e.g. number of physicians, availability of electronic patient history, electronic drug-drug interaction control system, and drug distribution system (Appendix B). The final questionnaire investigated non-reporting and difficulties with coding the morbidity variables (Appendix C).

We constructed a variable "incident relevance" from the items "disturbance" and "endangering" of the patients; if any of them was graded with "medium" or higher, the variable relevance was set to "more", otherwise it was set to "less". Because of question ambiguity, we set coding of the item care-dependency to "missing" for patients younger than 20 years of age. Evans' Index – a prognostic index – was calculated by simple addition of the number of chronic drug treatments with the number of chronic conditions [7].

The following free text variables were manually coded: relationship of the incident to the suspected medication, preventability of the incident, reactions to the incident, and proposals to avoid further incidents.

Statistical methods

Values are given as frequencies, mean ± SD or median [interquartile range (IQR)] (as denoted with (first quartile; forth quartile), depending on non-normal distribution or non-interval scaled data level. To assess the association of medication incidents with potential risk factors we used the SPSS-

For peer review only - http://bmjopen.bmj.com/site/about/guidelines.xhtml

GENLINMIXED procedure (a procedure that fits generalized linear mixed models). Clustering of patients was addressed by using a mixed binary logistic regression with the fixed factors of gender, year of birth, care-dependency, number of chronic drug treatments, number of chronic conditions, and TMI as well as the physicians' practice number as a random factor; if one item was missing, the whole record was excluded from the analysis. Numerator was affiliation to the case group; denominator was all PPC during weeks 11 and 12. We used IBM SPSS 23.

Results

The Sentinella system

During the year 2015, 149 practices were enrolled to the *Sentinella* system. Of them, 144 practices were known to report regularly; their properties are listed in Table 1. The *Sentinella* physicians are representative for the overall Swiss physician population [5,6]. Drugs were auto-distributed by 42% of the study practices. Approx. half of the physicians had electronic and the other paper-based medical records. Systematic drug-drug interaction control systems were installed only by a minority (36.8%) of the physicians. During the year 2015 (which included unusually for calendar adaptation, 53 instead of 52 reporting weeks), the general practitioners (GPs) had 4,456±2,137 PPC; for the pediatricians (PEDs), these were 5,297±2,715 (mean±SD). In their initial study questionnaire, 11.1% of the reporting physicians admitted to be working part time (i.e. less than 30 h per week).

Study flow

During the year 2015, we received 216 incident notifications (Figure 1). In 11 cases, we did not receive the detailed notification form. Eight cases had to be removed from the database because they fulfilled the exclusion criterion (i.e. "adverse drug reactions *without* detectable error"), and one case had to be removed because of double data entry. This led to 197 cases which could be analyzed. The distribution of the monthly incident notifications throughout 2015 is depicted in Figure e1 (Appendix D), and the distribution of the numbers of cases reported by each practice in Figure 2.

Description of patients

Table 2 lists age, gender and geographic distribution as well as the physician-to-patient relationship and the observer of the incident. Only three cases evolved in PED practices. No statistically significant differences were found between the two relevance classes of the incidents.

Number of incidents, non-reporting

For peer review only - http://bmjopen.bmj.com/site/about/guidelines.xhtml

In the GPs 194 incidents, 148 physicians, 4,456 yearly PPC led to 1.31 incidents per physician per year or 29.4 per 100,000 PPC; in PEDs 3 incidents, 32 physicians, 5,297 yearly PPC led to 0.1 incident per physician per year or 1.8 per 100,000 PPC.

To evaluate the *non-reporting of incidents*, we asked the physicians in the final study questionnaire. Out of 180 actively reporting physicians we received 145 questionnaires (80.6% response rate). To our question: “Did you **not** report medication incidents that you had noticed during the last year?”, they answered: “Never or almost” 110 (75.9%), “yes, but seldom” 22 (15.2%), “yes, frequently” 9 (6.2%), “always, or almost” 4 (2.8%). Reasons for not reporting incidents were “lack of time” or “forgetfulness”. If we speculate that these answers represent reporting rates of 76-100, 51-75, 26-50, or 0-25% respectively, we could divide the observed rates by the middle of the reporting classes: 0.875, 0.625, 0.375 and 0.125. By doing so, we calculated a rate of 50% of underreporting; if we furthermore consider the 5% of the incidents where the questionnaires were not sent, this rate increases to 58%. We therefore have to multiply the observed rates with a factor of 1.58 resulting in the following rates of detected incidents: GP 2.07 per physician per year, 46.5 per 100,000 PPC and PED 0.15 per physician per year, 2.8 per 100,000 PPC.

Types and causes of incidents, organ systems involved, preventability

The *types of error* are listed in Table e1 (Appendix D) and Figure 3; in 26 cases, more than one was mentioned. Most cases were linked to an incorrect dosage for a given patient, while prescription of an erroneous medication was the second most common error.

Most errors concerned orally ingested medication. Errors in application of *parenteral drugs* were reported less frequently; in our study, they comprised insulin (one case) and vaccinations (three cases). There were cases of an incorrect (influenza vs. anti-tetanus) or incomplete (Boostrix® vs. Boostrix-Polio®) vaccination and of undue vaccination (a third anti-HPV vaccine). One case was due to a communication problem within the practice staff, another to a vaccination in absence of the vaccination card.

Three cases concerned pediatric patients, all were linked to inadequate dosing, two times of an antibiotic prescription (Cotrimoxazole, Co-Amoxicillin) and one time of an antiemetic drug (Thiethylperazine).

In 89 of the 195 cases, *organ system* damage was reported, in 13 cases more than one organ system was involved, most frequently the central nervous system (Figure 4) (Table e2, Appendix D). Possible *triggers* of the incident were reported in 194 of the 197 cases; in 45 cases, there was more than one single reason, most frequently lacking alertness of the reporting physicians or their

For peer review only

For peer review only - http://bmjopen.bmj.com/site/about/guidelines.xhtml

staff (Table e3). When asked, who might be the "*responsible*" for the incident, 191 replied. The most common person or institution possibly responsible for the medication incident was: the reporting physician 41 (21.5%), followed by the practice nurse 26 (13.6%), the institution where the patient lives 33 (17.3%), the pharmacy 7 (3.7%), the hospital 12 (6.3%), the community nurse 4 (2.1%), the patients or their proxies 15 (7.9%), and the manufacturer 2 (1.0%); in 9 cases (4.6%) this was unclear, in 37 cases (19.4%), there was more than source one to blame. *Preventability* of the incidents was classified (by our study board): unlikely in 6 cases (3.0%), possible in 58 (29.4%), probable in 114 (57.9%), and definite in 19 (9.6%).

Endangering and disturbances

In 192 of the 197 cases the item "patients' *endangering"* as estimated by the physicians was answered. In 39 cases (20.3%) there was no endangering, in 83 (43.2%) light, in 51 (26.6%) moderate, in 19 (9.9%) severe. The answers to item "*disturbances* caused by the incident" are listed in Table 3. Out of the 197 incidents, 74 (37.5%) were classified as "more" relevant (see methods section, Table 2).

Interface problems, orientation, predictability, repeat incidents

The presence of *interface problems* was reported in 64 of 197 cases (32.4%); these were: with a hospital in 28 cases (43.8%), with an institution in 14 (21.9%), with a community nurse in 6 (9.4%), with a pharmacist in 9 (14.0%), with a specialist in 3 (4.7%), and with others in 4 (6.3%). In 184 cases, we received information about *informing* patients about the incident; in 98 cases, the patient was oriented by the reporting physician or his staff (50.3%), non-orientation was stated: because the patient was not able to understand (children, demented) in 26 cases (14.1%), because the problem had already been solved and the notification would have unduly disturbed the confidence in 18 cases (9.8%), because the patient or the proxies had observed themselves in 23 cases (12.5%), because the patient had already been oriented by others in 7 cases (3.8%) or because of another reason in 12 cases (6.5%). As for *predictability* of the incident, we received 183 valid answers; of them 82 (44.8%) stated "yes, in the given constellation, the incident was to be expected". When asked whether they had already reported a *similar incident* in the study, 29 out of 196 (14.8%) answered in the affirmative.

Risk factors to undergo a medication incident

To detect patient *risk factors* to undergo an incident, we compared the incident data with those of a fortnight denominator analysis [6]; the following univariate factors accumulated preferentially in incident patients: higher age, care-dependency, higher numbers of chronic conditions or medications (or higher Evans' Index), as well as higher TMI (table 4). In the logistic regression only inpa-

For peer review only

tient care by an institution remained a significant factor. Other suspected risk factors are listed in Table e4 (Appendix D); these items were not included in the denominator study and therefore lack a comparator. Within the patient group with a more as compared to a less relevant incident, only psychiatric illness reached a significantly increased proportion. We did not detect major differences between the two drug distribution systems.

Relationship between incidents and drug class, ATC-group

We assessed semi-quantitatively whether the class of the suspected drug was causally linked to the emergence of the incident; the frequencies were: none 49 (25.1%), unlikely 31 (15.9%), possible 77 (39.5%), probable 34 (17.4%), definite 4 (2.1%), did not apply 2 (n.a). ATC codes of the suspected medications are listed in Table 5. Most naming concerned the groups C “cardiovascular” (23.2%) and N “nervous system” (22.1%). Among the medication classes judged to be *related* to the incident (n=37) the most frequently named group were oral anticoagulants: rivaroxaban 9, phenprocoumon 7, and acenocoumarol 2 cases; this explains the 7-fold increased relative risk of ATC group B as compared to sales. As an example for errors *without* relation to the drug class, we must mention the 17 cases of institutionalized patients ingesting medications scheduled for other residents, in most cases this was person eating at the same table, but in other cases the person in care mixed up patient names.

Reactions to the incident and suggestions to prevent further ones

In 141 of the 197 cases, the physicians reported to have changed something after the incident, in 13 cases this was more than one *single action*, mostly often named were communication with other caregivers or better instruction of patients (Table e5, Appendix D). The respondents were asked for proposals how to *prevent* future incidents of the reported type; 125 of them made a total of 243 suggestions (Table e6, Appendix D). Of these, 37 (15.2%) were related to accurate medication lists, 10 (4.1%) to better patient instructions, 38 (15.6%) to organization of regular follow-up controls, and 55 (22.6%) involved organizational changes within the practice and its staff.

Discussion

In a representative group of primary care physicians [6,8], we found approximately one case of a medication incident per GP and year.

Incident rates

Incident rates in this study were similar to those found in other studies. We calculated the rates of detected medication incidents as follows: GP 2.07 per physician and year, 46.5 per 100,000 PPC and PED 0.15 per physician and year, 2.8 per 100,000 PPC. Medication incidents may make up a

For peer review only

proportion of approximately one third of all incidents [9]; the rates for *all safety incidents* may amount 6.20 per physician and year or 139.4 per 100,000 PPC in GPs and 0.45 per physician and year or 8.4 per 100,000 PPC in PEDs.

In Australian primary care patients, an incident rate of 4.98 per GP and year was reported; this is close to our estimated rate of 6.20; however, there was no sub-typing of the incidents [10]. In a three-year study, O'Beirne and colleagues reported a rate of 1.8 safety incidents per year and physician [9]. In a literature review of western countries' publications, Sandars and Esmail described a rate of 5 to 80 errors per 100,000 consultations [2], a rate somewhat lower than the 139.4 cases per 100,000 consultations estimated in our study. Kuo and colleagues reported a proportion of 15% of all incidents to be related to medication [11]; since we estimated a proportion of 33% this would give rise to even higher rates of all safety incidents when calculated from our study database. An 11% group of the physicians worked part time, the rate per full time physician per year may therefore be somewhat higher than calculated. The 13-fold higher incident rate in GPs as compared to PEDs is not surprising given the lower medication rates in children. A recent British study confirmed this much lower rate of incidents in children; out of 46,902 family practice safety reports, 1,788 concerned children (26-times less than adults) [12].

Definition of incidents and reliability of reporting

On the other hand, incident rates may be influenced by their definition. Gandhi and colleagues coined the term "avoidable adverse drug reaction" [13]. In our study, we explicitly excluded adverse drug reactions *without* detectable error. Runciman and colleagues defined a patient safety incident as follows: "An event or a circumstance that could have resulted or did result in unnecessary harm to a patient" [14]. An intuitive definition of a medical error was given by Makeham et al: "That was a threat to patient wellbeing and should not happen. I don't want it to happen again." [15]. As shown in Figure e1 (Appendix D), reporting frequency was higher at the beginning of the study as compared to the later course of it. This could reflect some loss of interest or forgetfulness by the reporting physicians. As calculated from our final questionnaire after the study, the reporting physicians failed to report about one in three cases of the detected medication errors. Non-detection of incidents may even be more frequent than non-reporting of observed incidents. A missed possible drug-drug interaction may be detected by chart review, a documentation error would probably have been found only in 1:1 supervision, which is very time-consuming, costly and may additionally influence performance of the observed physician. It is therefore virtually impossible to decipher the real rate of non-detected incidents. The problems in detection of incidents were the reason to postulate a "mix of methods" as needed to identify adverse events in general practice [16].

For peer review only

For peer review only - http://bmjopen.bmj.com/site/about/guidelines.xhtml

Other approaches to investigate safety incidents

In contrast to our prospective investigation, there are other methods to approach safety incidents. In a retrospective, semi-quantitative analysis, Gehring and colleagues investigated safety incidents in Swiss primary care [17]; among 23 predefined classes of safety incidents, the respondents admitted 15 to have occurred at least yearly – four of them being linked to drug treatment. Another approach to incidents is to ask patients as performed by Mira and colleagues [18]; they interviewed patients (> 65 years with five or more chronic drug treatments) and found that 75% of the patients reported to have been affected by at least one medication error during the previous twelve months. We did not collect data on appropriateness of treatment; admittedly medication inappropriate for some groups of patients (elderly or patients with impaired renal function) may provoke incidents [19]. A recent Scottish study demonstrated an impressive reduction in high-risk prescriptions (non-steroidal anti-inflammatory drugs, antiplatelet, and anticoagulation) as well as hospital admissions for gastrointestinal ulcers or heart failure by a combination of educational measures, informatics and financial incentives [20].

Type, consequences, causes and preventability of error

Most cases in this study involved application of an erroneous dosing or of a wrong medication although non-application of necessary medication was also frequent. The distribution of the incidents was similar to that reported in other studies [11,17,18,22]. When a wrong medication was dispensed, the error was often caused by confounding of prepared medication in home residents. The classic case of non-application of a necessary medication was the missed re-uptake of anticoagulation after an operation. Non-application of necessary drugs seems to be a relevant source of unnecessary harm to patients [21]. The causing of the incidents was similar as reported by others [11,17,18,22]. Most studies found – as in our patients – lacking alertness of and communication problems within practice staff, but there was a large variety of other topics. All three pediatric cases were linked to prescription of an inadequate dosing for age and weight. More than half of the patients did not have any disturbances after the incident; otherwise in most cases the nervous system was affected. No fatalities were reported, but seven patients (3.5%) needed stationary care. In 2004 Pirohamed and colleagues published a study on adverse drug reactions as a cause for admission to hospital; they found that 6.5% of all hospitalizations and 4.0% of all hospital days were caused by adverse drug reactions, 72% of them being preventable and 2% leading to death [23], however, this study included all cases, and therefore a majority of cases of adverse drug reactions *without* a detectable error. An older Swiss study was published by Livio and colleagues; out of 3,195 hospitalizations, she identified 229 cases (7.2%) as probably caused by adverse drug reactions [24]. In that study, 32% of the events were classified as being preventable (which does not

For peer review only - http://bmjopen.bmj.com/site/about/guidelines.xhtml

strictly mean that an error had caused the incident), and in 6% of the cases, fatalities were reported. The results on outpatients contrast to inpatients where one in ten drug administrations was described to be erroneous [25]. Medical errors seem to be the “third leading cause of death in the US” [26].

Risk factors

We were able to identify some risk factors such as higher age, care-dependency, higher numbers of chronic conditions or medications (and higher Evans’ Index), as well as higher TMI. However, adjusted in the logistic regression analysis, only care-dependency remained a significant risk factor, but this may be due to the small number of observations which possibly precluded less important risk factors to be detected. The only significant risk factor for undergoing an incident of higher relevance was psychiatric disease. Several factors which reflect quite well the results of our study have been described in the literature to correlate with proneness to undergo a medication incident: mainly the number of drugs ingested [13,27], but also higher or young age [28] and morbidity [29,30] have been described. Most incidents concerned ATC-groups N “nervous system” or C “cardiovascular”, which were also of the mostly sold drugs; an exception was group B with anticoagulants and a sevenfold increased relative risk as compared to Swiss sales in 2015. It seems wise to be alert to avoid errors when prescribing medication of these groups. The prevailing position of anticoagulants (18.5% of cases) was described also by Field and colleagues [30]. Otherwise the repartition of our suspected drugs was similar to other primary care studies [11,30], a literature review [31] or a theoretical paper [22].

Limitations

There is likely – as expected – bias from selective and underreporting or non-detection of medication incidents. Therefore, the true rate of incidents could only be estimated, and the proportions of incident characteristics would probably substantially have been altered by a more complete recording of incidents.

Conclusion

Medication incidents are common in general medicine whereas they rarely occur in pediatrics, in which polypharmacy is less prevalent. Reasons for medication incidents are diverse but often seem to be linked to communication problems. Older and multimorbid patients are at a particularly high risk for medication incidents.

For peer review only

For peer review only - http://bmjopen.bmj.com/site/about/guidelines.xhtml

Abbreviations

GP general practitioner

PED pediatrician

PPC patient-to-physician contacts

TMI Thurgau Morbidity Index

Author affiliations

MG Institute of Primary Care, University of Zurich, Switzerland.
AC Division of Clinical Pharmacology and Toxicology, Institute of Pharmacological Sciences of Southern Switzerland, Ente Ospedaliero Cantonale, Lugano, and Department of Clinical Pharmacology and Toxicology, University Hospital Zurich, Zurich, and National Poisons Centre, Tox Info Suisse, Associated Institute of the University of Zurich, Zurich, Switzerland
DC Patientensicherheit Schweiz, Zurich, Switzerland.
LH Policlinique Médicale, University of Lausanne, Switzerland and Sentinel Surveillance Network, Swiss Federal Office of Public Health, Bern, Switzerland.
MP Epidemiology, Biostatistics, and Prevention Institute, University of Zurich, Switzerland.
AS Sentinel Surveillance Network, Swiss Federal Office of Public Health, Bern, Switzerland.
MZ Institute of General Medicine, University of Zurich, Switzerland.

Acknowledgements

We are grateful to Lee Wennerberg for the English language corrections, Dr Sven Staender, Männedorf for the helpful comments, and Simon Gnädinger, Zurich for the manual coding of free text items. We thank the *Sentinella* program commission for their support, the reporting physicians of *Sentinella* for their unflagging enthusiastic collection of data, and the Federal Office of Public Health for providing data and translating the questionnaires into French.

Contributorship statement

MG lead the study, did the pilot study (questionnaire development, data entering and processing) wrote all documents, did all the contacts with the *Sentinella* administration, ethics committee, and

For peer review only

others, programmed the electronic questionnaires, entered hand-written questionnaires into the database, did the data processing and wrote the publication after data collection.

AC is an expert on clinical pharmacology and drug safety.

DC is an expert on patient safety.

LH is French-speaking and helped to interpret the French questionnaires. She is an expert on multimorbidity. She is a member of the *Sentinella* program commission.

MP is head of Epidemiology, Biostatistics & Prevention Institute. He is responsible for the sound methodology.

AS had the idea for the study. He is vice president of the *Sentinella* program commission.

MZ is expert on electronic data exchange in primary care.

All of them have seen all the study documents and have contributed intellectually to their elaboration. All have contributed to revise the draft of this publication and approve the submitted version of this publication.

Funding

The study was funded by Bangerter-Rhyner Foundation, Basel.

Competing interests

The authors declare that they have no financial interest conflicts with this study.

Patient consent

Not necessary for anonymous data.

Ethics approval

The ethical committee of Canton Zurich decided that our study did not need formal approval because the data are completely anonymous (KEK-ZH 2014-0400). The study was recorded in www.ClinicalTrials.gov: NCT02295371, as well as in our national study registry (www.kofam.ch; SNCTP000001207).

Data sharing statement

No additional data are available.

Strobe statement

For peer review only

Whenever possible, the guidelines of the Strobe statement were followed (Appendix E).

References

1. **Manias E:** Detection of medication-related problems in hospital practice: a review. *Br J Clin Pharmaco*l 2012; 76 (1): 7-20.
2. **Sandars J, Esmail A:** The frequency and nature of medical error in primary care: understanding the diversity across studies. Fam Pract 20 (3): 231-6, 2003.
3. **Miller GC, Britth HC, Valenti L.** Adverse drug events in general practice patients in Australia. *Med J Aust.* 2006;184(7):321-324.
4. **Thomsen LA, Winterstein AG, Søndergaard B, Haugbølle LS, Melander A.** Systematic review of the incidence and characteristics of preventable adverse drug events in ambulatory care. *Ann Pharmacother.* 2007; 41(9):1411-1426.
5. **Gnädinger M, Ceschi A, Conen D, et al:** Medication incidents in primary care medicine: protocol of a study by the Swiss Federal Sentinel Reporting System. BMJ Open 04/2015; 5(4): e007773. DOI:10.1136/bmjopen-2015-007773.
6. **Gnädinger M, Herzig L, Ceschi A et al:** The Burden Related to Chronic Conditions and Multimorbidity in the Swiss Primary Care Population: A Prospective Study in the Swiss Sentinel Surveillance Network (*Sentinella*), 2016, to be submitted. **That manuscript is included in the present submission.**
7. **Evans DC, Cook CH, Christy JM et al:** Comorbidtiy-polypharmacy scoring facilitates outcome prediction in older trauma patients. J Am Geriatr Soc 60: 1465-70, 2012.
8. **Cohidon C, Cornuz C, Senn N:** Primary care in Switzerland: evolution of physicians‘ profile and activities in twenty years (1993-2012). BMC Fam Pract 16: 107, 2015.
9. **Makeham M, Dovey S, Runciman W, et al:** Methods and Measures used in Primary Care Patient Safety Research. Results of a literature review. WHO report on patient safety Qual Safety Health Care 2008.
10. **Makeham MAB, Kidd MR, Saltman DC et al:** The threats to Australian patient safety (TAPS) study: incidents of reported errors in general practice. Med J Aus 185: 95-98, 2006.
11. **Kuo GM, Phillips RL Graham D et al:** Medication errors reported by US family physicians and their office staff. Qual Saf Health Care 17: 286-90, 2008.
12. **Rees P, Edwards A, Panesar S et al:** Safety incidents in the primary care office setting. Pediatrics 135 (6): 1027-35, 2015.

For peer review only - http://bmjopen.bmj.com/site/about/guidelines.xhtml

13. **Gandhi TK, Weingart SN, Borus BA, et al:** Adverse drug events in ambulatory care. N Engl J Med 348: 1556-64, 2003.

14. **Runciman W, Hibbert P, Thomoson R et al:** Towards an international classification ofr patient safety: key concepts and terms. Int J Qual Health Care 21 (1): 18-26, 2009.

15. **Makeham MAB, Dovey SM, County M et al:** An international taxonomy for errors in general practice: a pilot study. Med J Aus 177:68-72; 2002.

16. **Wetzels R, Wolters R, van Weel C et al:** Mix of methods is needed to identify adverse events in general practice: a prospective observational study. BMC Fam Pract 2008; 9:35.

17. **Gehring K, Schwappach DLB, Battaglia M et al:** Frequency of and harm associated with primary care safety incidents. Am J Managed Care 18 (9): e323-7, 2012.

18. **Mira JJ, Orozco-Beltran D, Perez-Jover V et al:** Physician patient communication failure facilitates medication errors in older polymedicated patients with multiple comorbidities. Fam Pract 30: 56-63, 2013.

19. **Cooper JA, Moriarty F, Ryan C et al:** Potentially inappropriate prescribing in two populations with differing socio-economic profiles: a cross-sectional database study using the PROMPT criteria. Eur J Clin Pharmacol 72: 583-91, 2016.

20. **Dreischulte T, Donnan P, Grant A et al:** Safer prescribing – a trial of education, informatics, and financial incentives. New Engl J Med 374: 1053-64, 2016.

21. **O'Grady I, Gerrett D:** Minimising harm from missed drug doses. Nursing Times 111 (44): 12-15, 2015.

22. **Huges RG, Ortiz E:** Medication errors. Why they happen, and how they can be prevented. Am J Nursing 205 (3): 14-24, 2005.

23. **Pirmohamed M, James S, Meakin S, et al:** Adverse drug reactions as cause of admission to hospital: prospective analysis of 18 820 patients. BMJ 2004; 329:15–19.

24. **Livio F, Buclin T, Yersin B et al:** Hospitalisations pour effet indésirable médicamenteux. Recensement prospectif dans un service d'urgences médicales [French]. Raisons de Santé 23, 1998.

25. **Berdot S, Gillaizeau F, Caruba T et al:** Drug administration errors in hospital inpatients: a systematic review. PLoS ONE 8 (6): e68856, 2013; doi: 10.1371/journal.pone.0068856.

26. **Makary M, Daniel M :** Medical error – the third leading cause of death in the US. BMJ 2016 ; 353: i2139.

For peer review only

For peer review only - http://bmjopen.bmj.com/site/about/guidelines.xhtml

27. **Nobili A, Pasina L, Tettamanti M et al:** Potentially severe drug interactions in elderly outpatients: results of an observational study of an administrative prescription database. J Clin Pharm Ther 34: 377-86, 2009.

28. **Avery AJ, Ghaleb M, Barber N, et al:** The prevalence and nature of prescribing and monitoring errors in English general practice: a retrospective case note review. Br. J Gen Pract e543, 2013.

29. **Field TS, Gurwitz JH, Avorn J et al:** Risk factors for adverse drug events among nursing home residents. Arch Intern Med 161: 1629-34, 2001.

30. **Field TS, Mazor KM, Briesacher B, et al:** Adverse drug events resulting from patient errors in older adults. J Am Geriatr Soc 55: 271-6, 2007.

31. **Saedder EA, Brock B, Nielsen LP et al:** Identifying high-risk medication: a systematic literature review. Eur J Clin Pharmacol 70: 637-45, 2014.

For peer review only

For peer review only - http://bmjopen.bmj.com/site/about/guidelines.xhtml

List of figures and tables

Figure 1: Study flow chart

Figure 2: Distribution of the number of cases reported by practice

Figure 3: Type of error

Figure 4: Organ system involved

Table 1: Characteristics of the reporting physicians in 2015

Table 2: General description of the cases

Table 3: Disturbances after the incident

Table 4: Possible risk factors for incident as compared to a denominator analysis during calendar weeks 11 and 12 [Gnädinger M 2016]

Table 5: ACT-Groups of suspected medications

For peer review only

For peer review only - http://bmjopen.bmj.com/site/about/guidelines.xhtml

Physicians' gender	
- male	128 (71.1%)
- female	52 (28.9%)
Physicians' age class	
- < 40	12 (6.7%)
- 40-49	44 (24.4%)
- 50-59	66 (36.7%)
- 60 and over	58 (32.2%)
Specialty	
- GP	148 (82.2%)
- PED	32 (17.8%)
Number of physicians in practice (n=144)	
- 1	69 (47.8%)
- 2	39 (27.1%)
- 3	17 (11.8%)
- 4 to 5	8 (5.6%)
- 6 to 9	7 (4.9%)
- 10 and over	4 (2.8%)
Number of physicians per practice reporting to *Sentinella* (n=144)	
- 1	119 (82.6%)
- 2	19 (13.2%)
- 3	5 (3.5%)
- 8	1 (0.7%)
Linguistic region	
- German	122 (67.8%)
- French	44 (24.4%)
- Italian	14 (7.8%)
Urbanity of the practice	
- urban	93 (51.7%)
- agglomeration	60 (33.3%)
- rural	27 (15.0%)
Workload per week	
- < 15 h	9 (5.0%)
- 15-30 h	36 (20.0%)
- > 30 h	135 (75.0%)
Drug distribution system	
- dispensing by physician	73 (42.2%)
- mixed system	19 (10.6%)
- dispensing by pharmacy	85 (47.2%)
Electronic documentation	
- yes	89 (49.4%)
- no	91 (50.6%)
Electronic interaction control	
- yes	65 (36.1%)
- no	115 (63.9%)
Electronic prescription	
- yes, with thesaurus	62 (34.4%)
- yes, but without thesaurus	24 (13.3%)
- none	94 (52.2%)
Certification of the practice	
- yes	46 (25.6%)
- none	134 (74.4%)
Staff meetings	
- yes, at least monthly	69 (38.3%)
- yes, but less frequently	70 (38.9%)
- none	41 (22.8%)
Quality circle participation	
- yes, at least monthly	134 (74.4%)

For peer review only - http://bmjopen.bmj.com/site/about/guidelines.xhtml

-	yes, but less frequently	23 (12.8%)
-	none	23 (12.8%)

Table 1. Characteristics of the reporting physicians in 2015 (144 practices, 180 physicians)
.

For peer review only

	Relevance		All
	less	more	
Number of cases	124	73	197
Patients age	69.2 ± 20.6	69.4 ± 21.2	69.3 ± 20.8
Patients gender,			
% males	40.3	32.9	37.6
Physicians specialty			
% pediatricians	1.6	1.4	1.5
Linguistic region, %			
- German	75.0	68.5	72.6
- French	18.5	28.8	22.3
- Italian	6.5	2.7	5.1
Physician-to-patient relationship, %			
- own family physician	86.3	78.1	83.2
- urgency / holiday replacing	0.8	4.1	2.0
- institution physician	11.3	17.8	13.7
- other	1.6	0.0	1.0
Observer of the incident; %			
- physician / practice staff	50.0	50.7	50.3
- patient / proxies	21.8	23.3	22.3
- community nurse	1.6	4.1	2.5
- institution (where patient lives)	15.3	16.4	15.7
- hospital	0.8	1.4	1.0
- other physicians	2.4	0.0	1.5
- pharmacist	7.3	4.1	6.1
- other	0.8	0.0	0.5

Table 2. General description of the cases. Differences between “less” and “more” categories are not statistically significant. We calculated the variable “incident relevance” from the variables “dis-

For peer review only - http://bmjopen.bmj.com/site/about/guidelines.xhtml

turbance” and “endangering” of the patients; if any of them was graded with “medium” or higher, the variable relevance was set to “more”, otherwise to “less”.

For peer review only

For peer review only - http://bmjopen.bmj.com/site/about/guidelines.xhtml

Item	N	percent
Severity of disturbance		
- no symptoms but pathological laboratory tests	15	8.0
- light	44	23.4
- moderate	22	11.7
- severe	10	5.3
- fatality	0	0.0
Subtotal (this is the base of the next rows)	*91*	*48.4*
- no symptoms, normal (or no) laboratory tests	97	51.6
Total	188	100.0
Missing data	9	n.a.
All patients	197	n.a.
Time until recovery		
- hours	26	28.5
- days	41	45.1
- weeks	15	16.5
- not yet known or missing information	9	9.9
All patients with disturbances	91	100.0
Recovering		
- without sequels	78	85.8
- with light to moderate sequels	2	2.2
- with severe sequels or fatality*	5	5.4
- not yet known or missing information	6	6.6
All patients with disturbances	91	100.0
Treatment / surveillance		
- not needed	48	52.7
- ambulatory care	33	36.3
- hospital care**	7	7.7
- missing information	3	3.3
All patients with disturbances	91	100.0

For peer review only

Table 3. Disturbances after the incident. * In one case, there was a reduced kidney function, the other cases remained unclear, and no fatalities were reported. ** Two cases had to be surveilled in the emergency room, the hospital stays were: intoxications with thiethylperazine, with fenoterol plus ipratropium bromide, with zolpidem, further a derailed diabetes type 2 (after missed treatment with metformin), and a gastro-intestinal hemorrhage in a patient where antithrombotic treatment with rivaroxaban was not communicated to the physician.

For peer review only

For peer review only - http://bmjopen.bmj.com/site/about/guidelines.xhtml

Item	Patient group		OR (95% CI) (crude)	OR (95% CI) (adjusted)
	Denominator	Incidents		
Number of observations	26,852	197	/	/
Age (mean±SD), years	46.7 ± 27.5	69.3 ± 20.8	1.004 (1.001;1.006)**[a]	1.001 (0.996;1.005)
Gender,				
● male	47.0	37.6	1	1
● female	53.0	62.4	1.048 (0.916;1.197)	1.055 (0.878;1.196)
Care-dependency, number (%)* [d]				
● none	16,335 (85.5%)	96 (51.6%)	1	1
● yes, by proxies	954 (5.0%)	12 (6.5%)	1.121 (0.789;1.594)	0.979 (0.674;1.423)
● yes, by community nurse	723 (3.8%)	22 (11.8%)	1.458 (1.025;2.073)[a]	1.201 (0.821;1.758)
● yes, by institution	1,099 (5.8%)	56 (30.1%)	1.802 (1.399;2.323)[c]	1.528 (1.141;2.046) [a]
Number of conditions (median, IQR)	2 (0;4)	5 (3;7)	1.052 (1.029;1.075)**[c]	1.030 (0.994;1.067)**
Number of chronic active treatments (median, IQR)	1 (0;4)	6 (3;9)	1.052 (1.030;1.074)**[c]	1.030 (0.995;1.067)**
Evans' Index (median, IQR)	3 (0;8)	11 (6;17)	1.009 (1.005;1.013)** [c]	n.a.***
Thurgau Morbidity Index value (%)[d]			[d]	
● 0	8,463 (31.5%)	24 (12.2%)	1	1
● 1	3,611 (13.4%)	8 (4.1%)	0.989 (0.787;1.242)	0.908 (0.694;1.190)
● 2	4,102 (15.3%)	23 (11.7%)	1.049 (0.847;1.300)	0.898 (0.685;1.169)
● 3	3,877 (14.4%)	39 (19.8%)	1.131 (0.914;1.399)	0.830 (0.611;1.127)
● 4	2,119 (7.9%)	39 (19.8%)	1.292 (1.004;1.662)[a]	0.901 (0.643;1.265)
● 5	1,539 (5.7%)	38 (19.3%)	1.420 (1.078;1.868)[b]	0.823 (0.547;1.239)
● 6	709 (2.6%)	26 (13.2%)	1.680 (1.178;2.396)[c]	0.866 (0.523;1.436)
missing values	18	0		

For peer review only - http://bmjopen.bmj.com/site/about/guidelines.xhtml

Table 4. Possible risk factors for incident as compared to a denominator analysis during calendar weeks 11 and 12 [Gnädinger M 2016]. * Because of question ambiguity this analysis was restricted to adult patients (age > 19 years); this led to 19,812 valid observations in the denominator and 183 in the incident groups. ** Per one conditions, medication, year or index point. *** Because Evans' Index is a composite of condition and medication numbers; it was not included in the multiple regression analysis. Significance levels: [a] $p<0.05$, [b] $p<0.01$, [c] $p<0.001$. [d] Because GENLIN procedure was not able to process ordinal scaled variables, correlations between study group and TMI or care-dependency were tested with Spearman's rho: the correlation coefficients were +0.075 or +0.094, respectively, $p<0.001$.

For peer review only

ATC-Class		All incidents	Incidents with *probable* or *definite* relationship with medication	Relative Risk (percentages left by right column)	Swiss 2015 sales* (number of packages)
A	Alimentary tract and metabolism	28 (14.6%)	6 (15.8%)	1.06	31,455,252 (14.9%)
B	Blood and blood forming organs	23 (12.0%)	7 (18.4%)	7.08	5,507,624 (2.6%)
C	Cardiovascular system	44 (22.9%)	1 (2.6%)	0.35	16,027,143 (7.5%)
D	Dermatologics	1 (0.5%)	0 (0.0%)	0.00	17,314,810 (8.2%)
G	Genito-urinary system and sex hormones	1 (0.5%)	0 (0.0%)	0.00	7,936,641 (3.7%)
H	Systemic hormonal preparations (excluding sex hormones and insulins)	7 (3.6%)	1 (2.6%)	2.00	2,875,760 (1.3%)
J	Anti-infectives for systemic use	23 (12.0%)	6 (15.8%)	3.95	8,444,623 (4.0%)
K	Infusion liquids	0 (0.0%)	0 (0.0%)	0.00	24,158,749 (11.5%)
L	Anti-neoplastic and immunomodulating agents	5 (2.6%)	1 (2.6%)	2.89	1,934,950 (0.9%)
M	Musculo-skeletal system	4 (2.1%)	2 (5.3%)	0.76	14,787,413 (7.0%)
N	Nervous system	43 (22.4%)	10 (26.3%)	1.30	42,690,195 (20.2%)
P	Anti-parasitic products, insecticides and repellents	1 (0.5%)	0 (0.0%)	0.00	462,559 (0.2%)
R	Respiratory system	7 (3.6%)	3 (7.9%)	0.57	28,837,468 (13.7%)
S	Sensory organs	2 (1.0%)	0 (0.0%)	0.00	7,658,311 (3.6%)
T	Diagnostic use	0 (0.0%)	0 (0.0%)	0.00	43,184 (0.0%)
V	Various	3 (1.6%)	1 (2.6%)	6.50	855,707 (0.4%)
	Total	192 (100.0%)	38 (100%)	1.0	210,990,389 (100.0%)

For peer review only

does not apply	5	0	n.a.	n.a.

Table 5. ACT-Groups of suspected medications. Relative risk of drugs with probable or definite relationship with the incident as compared to sales proportions. * Information by Interpharma Switzerland (Appendix F).

For peer review only

For peer review only - http://bmjopen.bmj.com/site/about/guidelines.xhtml

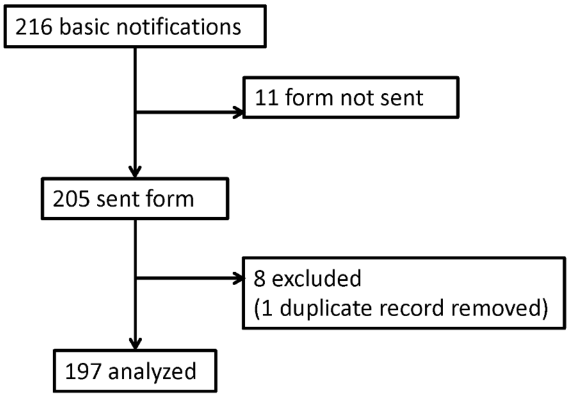

Study flow chart

254x190mm (300 x 300 DPI)

ew only

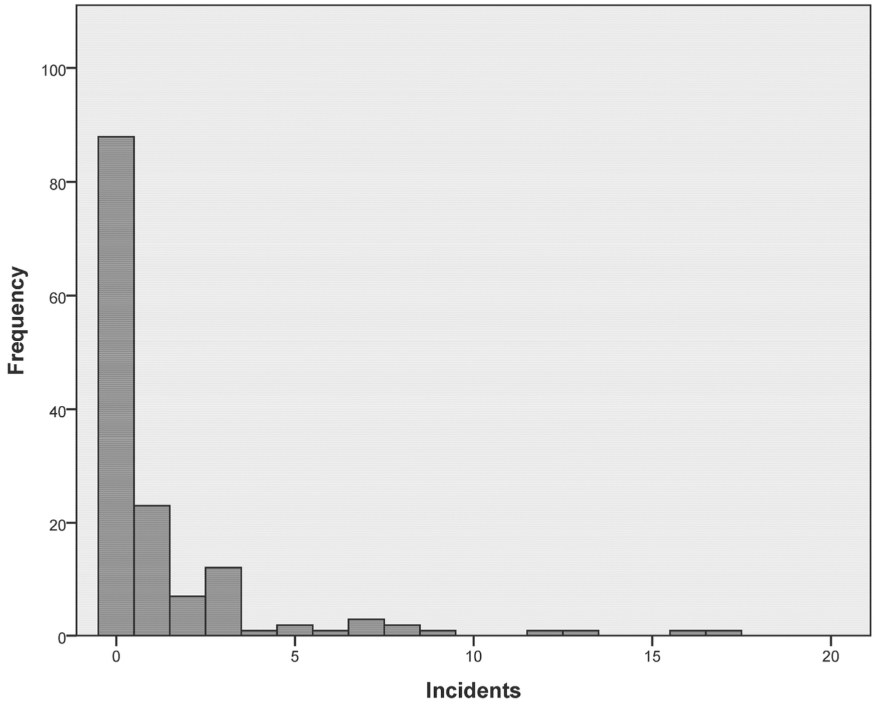

Distribution of the number of cases reported by practice

136x106mm (300 x 300 DPI)

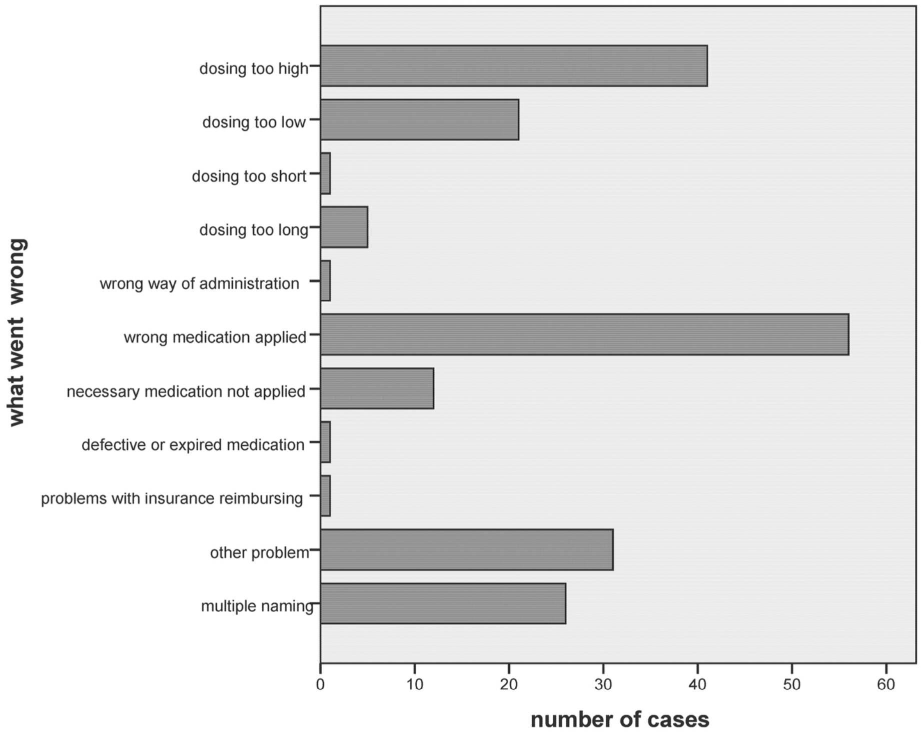

Type of error

148x129mm (300 x 300 DPI)

only

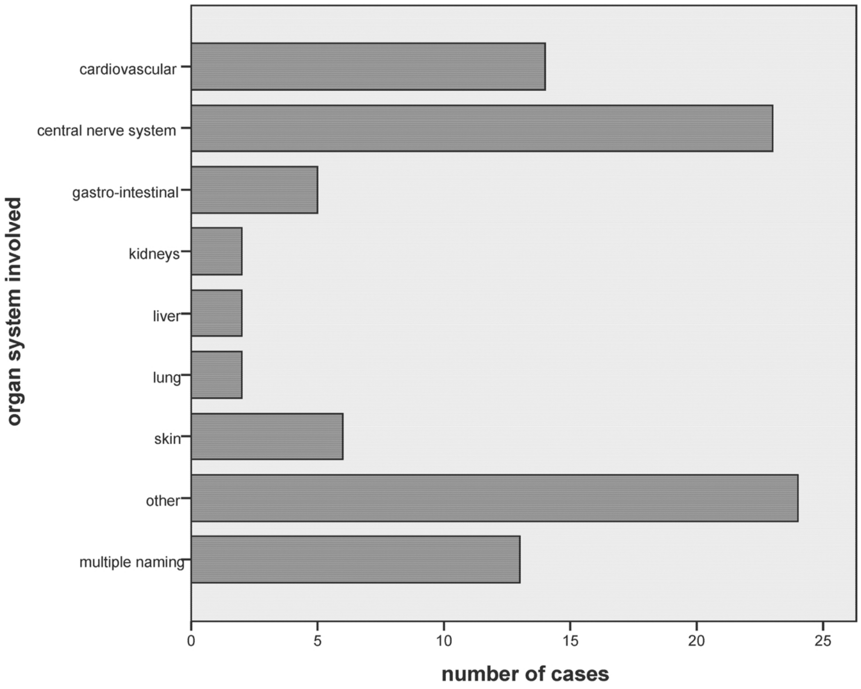

Organ system involved

147x120mm (300 x 300 DPI)

w only

Appendix A Incident questionnaire

Medication Incidents in Primary Care (MIPC)

(Version 1.0, January 25th 2015) (Internet version not available in English)

Incident reporting form

Administrative Information

1. Sentinella identification number: ☐☐☐☐ **2. Week of reporting:** ☐☐

The Patient

3. Year of birth: ☐☐☐☐ **4. Gender:** m ☐ f ☐

5. What was your relationship to the patient when the incident happened? Were you the

family physician ☐ emergency / substitute physician ☐ institution physician ☐ other ☐
if other what kind………………………

6. What is the patient's living situation?

with partner / family ☐ alone ☐ institution ☐ unknown ☐

7. Are there social problems?
yes ☐ no ☐ unknown ☐

8. Is the patient demented or otherwise mentally handicapped?

For peer review only - http://bmjopen.bmj.com/site/about/guidelines.xhtml

yes ☐ no ☐ unknown ☐

9. Does he suffer from psychological problems?

yes ☐ no ☐ unknown ☐

10. Does he take psychotropic drugs (esp. antidepressants, neuroleptics, benzodiazepines, opiates)?

yes ☐ no ☐ unknown ☐

11. Are there linguistic problems?

yes ☐ no ☐ unknown ☐

12. Does he or she smoke?

yes ☐ no ☐ unknown ☐

13. Is there substance abuse (other than nicotine)?

yes ☐ no ☐ unknown ☐, if yes, what substance? ……………………..

14. Does the patient have uncorrected / uncorrectable visual impairment?

yes ☐ no ☐ unknown ☐

15. Does the patient have uncorrected / uncorrectable hearing impairment?

yes ☐ no ☐ unknown ☐

16. Does the patient have uncorrected / uncorrectable mobility impairment?

yes ☐ no ☐ unknown ☐

For peer review only - http://bmjopen.bmj.com/site/about/guidelines.xhtml

17. Is there renal insufficiency (GFR: <60 ml/min/1.73 m^2)?

yes ☐ no ☐ unknown ☐

18. Is there hepatic insufficiency or liver cirrhosis?

yes ☐ no ☐ unknown ☐

19. Was the patient hospitalized in the past 12 months?

yes ☐ no ☐ unknown ☐

20. Is the patient taken care of by others? (*only one answer permitted*)?

yes, family / proxies ☐ yes, community nurse ☐ yes, institution ☐ no ☐ unknown ☐

21. Number of regularly applied active substances (including non-daily applied ones, see guidelines)?

☐☐ unknown ☐

22. Number of chronic conditions (see guidelines)?

☐☐ unknown ☐

23. Scale value of „Thurgau Morbidity Index" (chronic part, see guidelines)?

☐ unknown ☐

For peer review only - http://bmjopen.bmj.com/site/about/guidelines.xhtml

Details of the incident

24. Please, give a short **description** of the incident (in block letters):

25. Who observed the incident? (*multiple answers possible*)?

Physician / staff ☐ patient / relatives ☐ community nurse ☐ home / institution ☐ hospital ☐

other physicians ☐ pharmacist ☐ other ☐ unknown ☐

for „other" please specify: ……………………………

26. What happened (*multiple answers possible*)?

dosage too high ☐

dosage too low ☐

application too short ☐

application too long ☐

wrong administration route ☐

wrong medication ☐

indicated medication not received ☐

expired / defective medication ☐

problems with insurance reimbursement ☐

unclear / undefined ☐

other (please specify): ……………………… ☐

For peer review only - http://bmjopen.bmj.com/site/about/guidelines.xhtml

27. Please state the **trade name** of the medication used in the incident:

………………………………………………..

28. Please note other medication names, presuming they are relevant to the case.

none ☐ unknown ☐

29. How would you judge the degree of hazard to the patient during the incident?

mild ☐ medium ☐ severe ☐ none ☐ does not apply ☐ unknown ☐

30. How intense was the impairment (as judged by the patient)?

mild ☐ medium ☐ severe ☐ fatal ☐ no symptoms, but pathological lab values ☐
no impairment ☐ does not apply ☐ unknown ☐
If there wasn't *any* impairment, please skip to **question 34**.

31. How long did the impairment last?

hours ☐ days ☐ weeks ☐ longer ☐ unknown ☐

32. How was the recovery?
without residues ☐ with mild residues ☐ with severe residues / fatal ☐ unknown ☐

33. Which organ system was affected (*multiple answers possible*)?
cardiovascular ☐
central nervous ☐
gastro-enteral ☐
kidneys ☐
liver ☐
lung ☐
skin ☐
other, please specify ………………… ☐

34. Did you have to apply a specific surveillance or treatment for the incident?

yes, ambulatory ☐ yes, hospital ☐ no ☐ unknown ☐

If yes, please specify which? …………………………………………..

35. What factors contributed causally to the emergence of the incident (*multiple answers possible*)?

off duty hours ☐

communication failure within practice ☐

generic substitution by pharmacist ☐

hand-written prescription incorrectly interpreted ☐

conflicting multiple prescriptions ☐

lack of alertness within practice ☐

lack of documentation ☐

insufficient patient instruction ☐

lack of aids (e.g. Dosette®) ☐

lack of cooperation by patient / relatives ☐

misleading package leaflet information ☐

patient's internet search ☐

administrative problems ☐

manufacturer related (defective medication) ☐

distributer related (out of stock) ☐

lack of maintenance (e.g. first aid kit) ☐

other, please specify……………………….. ☐

unknown ☐

36. Was there an interface problem? If yes, which (*multiple answers possible*)?

yes, with hospital ☐

yes, with institution ☐

yes, with community nurse ☐

yes, with pharmacist ☐

For peer review only - http://bmjopen.bmj.com/site/about/guidelines.xhtml

yes, with specialist physician ☐
yes, with other, please specify? …………… ☐

no ☐

If **no**, please skip to **question 38**.

37. Was there an **explicit comparison** of prescription lists with the institution / person?

yes, verbal / by phone ☐ yes, written / by fax ☐ no ☐ unknown ☐

38/39. Was the patient informed about the incident?

yes, by myself / practice staff ☐
no, because he was not able to understand the message (children, demented) ☐
no, because the problem was solved and communication would have impaired confidence ☐
no, because the patient had moved or was deceased ☐
no, this was not needed because patient / relatives themselves had observed the incident ☐
no, because others had already informed him ☐

no, because: ………………………………………………………………………. ☐
unknown ☐

If yes, what was the patient’s reaction? ……………………………………………………

40. What did you do as a result to prevent similar incidents in the future (*multiple answers possible*)?
change standard operations procedures ☐
better instruction of patients ☐
communication with institution(s) ☐
notification of manufacturer ☐
notification of liability insurer ☐
notification of drug authority („yellow leaflet“) ☐
notification of the “critical incident reporting system” ☐
other, please specify? ……………………….. ☐
nothing ☐

41. Who or what was ultimately **responsible** for the occurrence of the incident? ……………………………………………

42. In the given situation, do you think one could have **anticipated** the event?

yes ☐ no ☐

43. Did you already report an **identical** or very similar incident to this study?

yes ☐ no ☐

44. Please make any **suggestions** about the kind of measures that could be taken to generally reduce the frequency of such events (in block letters):

Please keep a copy of this questionnaire in the patient files. Thank you for filling it out!

For peer review only - http://bmjopen.bmj.com/site/about/guidelines.xhtml

Appendix B Initial questionnaire

Medication incidents in primary care (MIPC)

Initial reporting (Version 1.0, January 25th 2015)
Internet version: not available in English

1. Sentinella identification number: ………….

2. Number of physicians within your practice: ………….

3. Number of them who report to Sentinella ………….

4. Your weekly workload, h/w: <16 ☐, 16-30 ☐, >30 ☐

5. Number of hours within your practice (%): <50% ☐, ≥50% ☐

6. Approximate proportion of medication prescribed (as compared to directly delivered drugs): <33% ☐, 33-66% ☐, >66% ☐

7. Do you have an X-ray (machine?)? yes ☐ no ☐

8. Do you have an ECG? yes ☐ no ☐

9. Do you have an ultrasound? yes ☐ no ☐

10. Do you have an electronic system for controlling electronic drug interaction? yes ☐ no ☐

11. Do you have electronic patient history documentation? yes ☐ no ☐

12. Do you prescribe electronically? yes, with a medication thesaurus ☐ yes, but without one (use of a typewriter) ☐ no ☐

13. Is your practice certified (e.g. EQUAM)? yes ☐ no ☐

14. Do you regularly schedule team sessions? Yes, at least monthly ☐, yes, but less frequently ☐, no ☐

For peer review only - http://bmjopen.bmj.com/site/about/guidelines.xhtml

15. Do you attend quality circle sessions (in accordance with "Hausärzte Schweiz")?
yes, regularly ☐, yes, now and then ☐, no ☐

16. Did you complete a special education (e.g. manual or psychosomatic medicine), or do you have special interests (e.g. toxic maniac patients)? yes ☐, no ☐
if yes, please specify : ……………………………………………………………………….

17. Are you contracted by an institution? yes ☐ no ☐
if yes, please specify (prison, home etc.): ………………………………………………………………

18. If yes, does this institution have specific problems with medication?
yes ☐, no ☐, if yes, please specify: ……………………………………………………………

19. Are you involved in other special activities (teaching, research, insurance doctor)?
yes ☐ no ☐
If yes, please specify the kind of activity: ……………………………………………………..

Thank you very much!

For peer review only - http://bmjopen.bmj.com/site/about/guidelines.xhtml

Appendix C Final questionnaire

Medication Incidents in Primary Care (MIPC)

Final questionnaire (Version 3.0 / December 14th 2015)

Online version: not available in English

Dear colleagues,

Thank you for your dedicated support of our study on medication incidents in 2015, regardless of whether you reported much of them or not. We would like to ask some final questions which will help us put the other information you sent us in the right context.

1. Sentinella identification number: ……………

2. Did you **not** report medication incidents that you had noticed during the last year (e.g. because of lack of time)?

never or almost never ☐, yes, but rarely ☐, yes, frequently ☐ always or almost always ☐

If this was frequently the case, please explain why:………………………………………..

3. Did your practice participate in the fortnight morbidity denominator study in March 2015 (calendar weeks 11 and 12)?
yes, fully ☐, yes, *but only partly (by omission of certain variables)* ☐, no ☐

If you did not fully participate or not at all, what was the reason?
……………………………………………………

If you did not participate in the denominator study, please continue with **question 12**.

4. How big was your effort for coding the morbidity variables of the denominator study?
manageable ☐, rather big ☐, too much ☐, impossible ☐

Did you have any difficulties when coding the morbidity variables?

5. Hospitalisation during the previous 12 months? none ☐, a little ☐, considerable ☐, severe ☐

6. Care-dependency? none ☐, a little ☐, considerable ☐, severe ☐

7. Number of medications? none ☐, a little ☐, considerable ☐, severe ☐

8. Number of conditions? none ☐, a little ☐, considerable ☐, severe ☐

9. Thurgau Morbidity Index? none ☐, a little ☐, considerable ☐, severe ☐

10. Repeat consultation during the fortnight? none ☐, a little ☐, considerable ☐, severe ☐

If you named considerable to severe difficulties in questions 5 to 10, please list them in **item 13**.

For peer review only - http://bmjopen.bmj.com/site/about/guidelines.xhtml

11. How long did it take for you and your practice nurse together for coding all of the variables of **one patient** (Dr. Gnädinger needed less than 3 minutes)?
………………………………………………. minutes

12. Would you be willing to be interviewed for a focus group on the subject of medication safety?

yes ☐, no ☐

13. Other comments:

Thank you very much!

For peer review only - http://bmjopen.bmj.com/site/about/guidelines.xhtml

Appendix D: electronic tables and figures

Figure e1: Distribution of the incident notifications over the year
Table e1: What went wrong with the incidents
Table e2: Organ system involved
Table e3: Causes of the incident
Table e4: Patient-sided possible risk factors
Table e5: Reactions to the incident
Table e6: Proposals to avoid further incidents

For peer review only - http://bmjopen.bmj.com/site/about/guidelines.xhtml

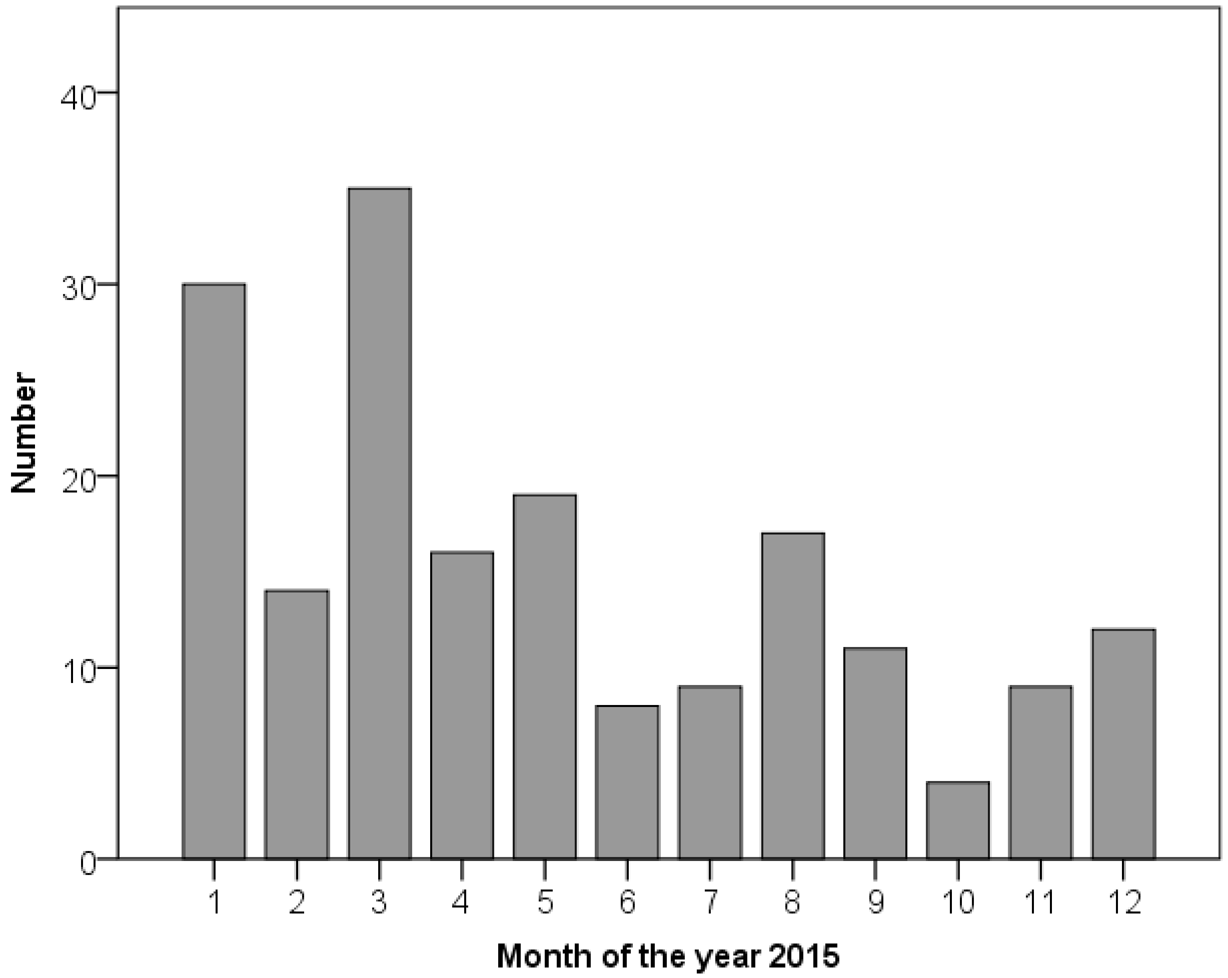

Figure e1. Distribution of the incident notifications over the year. Because only the week has been reported, the assignment to the month is approximate.

For peer review only - http://bmjopen.bmj.com/site/about/guidelines.xhtml

Type of error	N*	Percent*, %
Dosing too high	41 (12)	20.9 (6.1)
Dosing too low	21 (6)	10.7 (3.0)
Dosing too short	1 (2)	0.5 (1.0)
Dosing too long	5 (4)	2.6 (2.0)
Wrong way of administration	1 (1)	0.5 (0.5)
Wrong medication applied	56 (12)	28.6 (6.1)
Necessary medication not applied	12 (9)	6.1 (4.6)
Defective or expired medication applied	1 (1)	0.5 (0.5)
Problems with insurance reimbursing	1 (0)	0.5 (0.0)
Other problem**	31 (6)	15.8 (3.0)
Unknown	1 (0)	0.5 (0.0)
Multiple naming	26 (n.a.)	13.3 (n.a.)
Total	197 (53)	100.0 (26.9)

Table e1. What went wrong with the incidents. * Parenthesis denotes the additional naming within the category "multiple naming". ** Naming within the category "other" were: confusion of similar trade names (2), dosing error (6), erroneous package size (1), confusion of similar looking preparations (1), error when controlling blood levels (2), double dosing (4), missed discontinuation of medication (1), necessary treatment not applied (1), wrong vaccine applied (4), missed re-uptake of anticoagulation after operation (1), contra-indication overlooked (2), drug-drug interaction overlooked (3), known intolerance overlooked (3), necessary monitoring missed (2), transgression of maximal drug allowance (2), delivery of incomplete package (1), confusion by contradictory medication lists (3), incorrect handling of administering device (1), uncontrolled taking of "nature medicines" (1), unreliable compliance with medication plan (2), two medications of the same drug

For peer review only - http://bmjopen.bmj.com/site/about/guidelines.xhtml

class (3), forbidden bisection of pills (2), confusion of similar named patients (1), intake of medication of the neighbor resident in a home (1), other (9).

For peer review only - http://bmjopen.bmj.com/site/about/guidelines.xhtml

Organ system	N*	Percent*, %
Cardiovascular	14 (10)	15.4 (11.0)
Central nervous system	23 (10)	25.3 (11.0)
Gastro-intestinal	5 (3)	5.5 (3.3)
Kidneys	2 (2)	2.2 (2.2)
Liver	2 (0)	2.2 (2.2)
Lung	2 (1)	2.2 (1.1)
Skin	6 (0)	6.6 (0.0)
Other**	24 (6)	26.4 (6.6)
Multiple naming	13 (n.a.)	14.3 (n.a.)
Total	91 (32)	100 (35.2)
Question does not apply	106	/

Table e2. Organ system involved. * Parenthesis denotes the additional naming within the category “multiple naming”. ** Naming within the category “other” were: endocrine system (9), musculo-skeletal (8), ear-nose-throat (2), psychic (1), other (9).

For peer review only - http://bmjopen.bmj.com/site/about/guidelines.xhtml

Causes of the incident	N*	Percent*;%
Out of hours	2 (3)	1.0 (1.5)
Communication problems within staff	10 (11)	5.1 (5.6)
Generic substitution of original trade medication by pharmacist	3 (3)	1.5 (1.5)
Difficulties when reading hand-written prescription	1 (1)	0.5 (0.5)
Multiple conflicting prescriptions	5 (8)	2.5 (4.1)
Lacking alertness of physician or practice staff	50 (17)	25.4 (8.6)
Insufficient documentation	3 (11)	1.5 (5.6)
Insufficient patient instruction	7 (17)	3.6 (8.6)
Lacking cooperation of patient / proxies	6 (17)	3.0 (8.6)
Confusion by reading package leaflet	1 (0)	0.5 (0.0)
Confusion after “Googleing”	1 (0)	0.5 (0.0)
Administrative problems	2 (3)	1.0 (1.5)
Defective medication as caused by manufacturer	3 (0)	1.5 (0.0)
Lacking maintenance (e.g. emergency case)	1 (0)	0.5 (0.0)
Lacking use of treatment aids (e.g. Dosett)	0 (6)	0.0 (3.0)
Other source of trouble**	54 (18)	27.2 (9.1)
Unknown	3 (0)	1.5 (0.0)
Multiple naming	45 (n.a.)	23.1 (n.a.)
Total	197 (115)	100.0 (58.4)

Table e3. Causes of the incident. * Parenthesis denotes the additional naming within the category “multiple naming”. ** Naming within the category “other” were: Erroneous delivery in pharmacy (5), treatment delayed/hampered by patient (2), transcription error (2), missed

For peer review only - http://bmjopen.bmj.com/site/about/guidelines.xhtml

discontinuation of an ongoing treatment (2), communication problem within helpers' network (16), similar trade names (2), interface problems with hospital (4), reading error of patient / proxies (3), erroneous execution of a medical prescription (14), missed follow-up control (9), difficult handling of a preparation (2), disregard of possible interactions (2), transgression of competence by care workers (5), incomplete information by patient / proxies (8), stress / lack of time (12), other 25.

For peer review only - http://bmjopen.bmj.com/site/about/guidelines.xhtml

Item (number of missing observations in groups “less” / “more”)	Relevance less n (%)	more n (%)	All n (%)
Number of patients	124 (100.0)	73 (100.0)	197 (100.0)
Living situation (1/0)			
- together with mate / family	61 (49.6)	31 (42.5)	92 (46.9)
- alone	28 (22.8)	23 (31.5)	51 (26.0)
- institution	33 (27.6)	19 (26.0)	53 (27.0)
Social problems (2/4)			
- yes	22 (18.1)	18 (26.1)	49 (20.9)
Dementia or mental illness (2/2)			
- yes	31 (25.4)	18 (25.4)	49 (25.4)
Psychiatric problems (1/2)			
- yes	28 (22.8)	28 (38.4)*	56 (28.9)
Treatment with psychotropic drugs (0/0)			
- yes	50 (40.3)	39 (53.4)	89 (44.2)
Linguistic problems (0/1)			
- yes	11 (8.9)	5 (6.8)	16 (8.2)
Smoking (7/4)			
- yes	11 (9.4)	8 (11.6)	19 (10.2)
Substance abuse other than nicotine (2/1)			
- yes	3 (2.5)	5 (6.9)	8 (4.1)
Visual blurring (6/6)			
- yes	4 (3.4)	4 (6.0)	8 (4.3)

For peer review only - http://bmjopen.bmj.com/site/about/guidelines.xhtml

Hearing problems (3/5)			
- yes	8 (6.6)	4 (5.9)	12 (6.3)
Gait problems (2/2)			
- yes	40 (32.8)	18 (25.4)	58 (30.1)
Renal insufficiency (GFR<60 ml/min*1.73m^2) (6/2)			
- yes	24 (20.3)	21 (29.6)	45 (23.8)
Liver cirrhosis of other hepatic function problem (3/1) - yes	3 (2.5)	3 (4.2)	6 (3.1)

Table e4. Patient-sided possible risk factors. * p=0.021 "more" vs. "less" by chi-square testing. We calculated the variable "incident relevance" from the variables "disturbance" and "endangering" of the patients; if any of them was graded with "medium" or higher, the variable relevance was set to "more", otherwise to "less".

For peer review only - http://bmjopen.bmj.com/site/about/guidelines.xhtml

Reactions to the incident	N*	Percent*; %
Changing standard operating procedures of the practice	14 (3)	7.2 (1.5)
Better instruction of patients	23 (4)	11.7 (2.0)
Communication with other institutions	31 (3)	15.7 (1.5)
Notifying manufacturer	1 (2)	0.5 (1.0)
Reporting the incident to the critical incident reporting system	10 (6)	5.1 (3.0)
No reaction at all	55 (n.a.)	27.9 (n.a)
Other type of reactions to the incident**	49 (9)	24.9 (4.6)
Missing information	1 (0)	0.5 (0.0)
Multiple naming	13 (n.a.)	6.6 (n.a.)
Total	197 (27)	100.0 (13.7)

Table e5. Reactions to the incident. * Parenthesis denotes the additional naming within the category "multiple naming". ** Naming within the category "other" were: Hire more workforce (1), arrangements with other physicians (2), with pharmacist (4), with community nurse (4), with institution (12), with practice nurse (6), with specialist (1), with patient (3), with supplier (1), sending a new medication plan (1), having regular staff meetings (1), remove Digoxin 0.25 from assortment because of safety reasons (1), to not perform vaccinations in absence of vaccination card (2), to clarify intolerances (2), to clarify interactions (1), to hand out an allergy card (1), to actualize patient records (3), to apply for insurance cost credit (1), to organize follow-up controls (20), other (5).

For peer review only - http://bmjopen.bmj.com/site/about/guidelines.xhtml

Proposal	N*	Percent*;%
Cross-check of medication lists	6 (14)	4.8 (11.2)
Patient instructions	2 (3)	1.6 (2.4)
Reduction of time pressure / stress	3 (6)	2.4 (4.8)
To observe adverse drug reactions also with “nature products”	1 (0)	0.8 (0.0)
Four eyes check when dispensing medication	2 (4)	1.6 (3.2)
No medication without prescription	3 (6)	2.4 (4.8)
To critically audit polymedication	3 (2)	2.4 (1.6)
To avoid similarly looking or named medication in drug master	2 (3)	1.6 (2.4)
To demand medication plans to be brought to the consultation	0 (4)	0.0 (3.2)
To provide medication plans routinely	2 (8)	1.6 (6.4)
In-deep checking before delivering “new” medication to the patient	4 (1)	3.2 (0.8)
To provide patients with allergy / intolerance cards	2 (1)	1.6 (0.8)
To let patients themselves write their medication card (and controlling afterwards)	0 (3)	0.0 (2.4)
To instruct patients to come into the practice immediately after hospitalization	3 (0)	2.4 (0.0)
To broach regularly “medication safety” on staff meetings	5 (6)	4.0 (4.8)
To provide information in patients’ native language	0 (2)	0.0 (1.6)
To share important information about patients with practice nurse	0 (2)	0.0 (1.6)
To improve information flow	11 (16)	8.8 (10.4)
To organize follow-up controls	10 (28)	8.0 (22.4)
To wait for laboratory results before prescribing	1 (0)	0.8 (0.0)
Other**	12 (9)	9.6 (7.2)
Multiple answering	53 (n.a.)	42.4 (n.a.)

For peer review only - http://bmjopen.bmj.com/site/about/guidelines.xhtml

Total	125 (118)	100.0 (94.4)
None	72 (n.a.)	(n.a.)

Table e6. Proposals to avoid further incidents. * Parenthesis denotes the additional naming within the category “multiple naming”. ** Among naming within the category “other” were: timely involving of community nurse, stopping of self-medication in multimorbid or poly-medicated patients, to clearly labelling desensitization suspensions, to separating adult and pediatric vaccines, to implementing timely new guidelines, to refusing to be in care of a patient with bad compliance, to clearly separating and labelling of medication prepared for different patients, to advice patients for separating short- and long-acting insulins at their home, to providing short-time prescriptions with a clear expiration date, to allowing only adequately educated people to align and deliver medication in homes for the elderly and that delivering may be done by the same person who did the preparing of medication boxes, to avoiding similar looking medication boxes being muddled-up by patients at the refectory room.

For peer review only - http://bmjopen.bmj.com/site/about/guidelines.xhtml

Appendix E

STROBE Statement—Checklist of items that should be included in reports of ***cross-sectional studies***

	Item No	Recommendation
**Title and abstract**	1	(*a*) Indicate the study's design with a commonly used term in the title or the abstract **[yes]**
		(*b*) Provide in the abstract an informative and balanced summary of what was done and what was found **[yes]**
**Introduction**		
Background/rationale	2	Explain the scientific background and rationale for the investigation being reported **[yes]**
Objectives	3	State specific objectives, **[yes]** including any prespecified hypotheses **[no]**
**Methods**		
Study design	4	Present key elements of study design early in the paper **[yes]**
Setting	5	Describe the setting, locations, and relevant dates, including periods of recruitment, exposure, follow-up, and data collection **[yes]**
Participants	6	(*a*) Give the eligibility criteria, and the sources and methods of selection of participants **[yes]**
Variables	7	Clearly define all outcomes, exposures, predictors, potential confounders, and effect modifiers. **[yes]** Give diagnostic criteria, if applicable **[no]**
Data sources/ measurement	8*	For each variable of interest, give sources of data and details of methods of assessment (measurement). Describe comparability of assessment methods if there is more than one group **[yes]**
Bias	9	Describe any efforts to address potential sources of bias **[yes]**
Study size	10	Explain how the study size was arrived at **[yes]**
Quantitative variables	11	Explain how quantitative variables were handled in the analyses. If applicable, describe which groupings were chosen and why **[yes]**
Statistical methods	12	(*a*) Describe all statistical methods, including those used to control for confounding **[yes]**
		(*b*) Describe any methods used to examine subgroups and interactions **[yes]**
		(*c*) Explain how missing data were addressed **[yes]**
		(*d*) If applicable, describe analytical methods taking account of sampling strategy **[no]**
		(*e*) Describe any sensitivity analyses **[no]**
**Results**		

For peer review only - http://bmjopen.bmj.com/site/about/guidelines.xhtml

Participants	13*	(a) Report numbers of individuals at each stage of study—eg numbers potentially eligible, examined for eligibility, confirmed eligible, included in the study, completing follow-up, and analysed **[no]**
		(b) Give reasons for non-participation at each stage **[yes]**
		(c) Consider use of a flow diagram **[yes]**
Descriptive data	14*	(a) Give characteristics of study participants (eg demographic, clinical, social) and information on exposures and potential confounders **[yes]**
		(b) Indicate number of participants with missing data for each variable of interest **[yes]**
Outcome data	15*	Report numbers of outcome events or summary measures **[yes]**
Main results	16	(*a*) Give unadjusted estimates and, if applicable, confounder-adjusted estimates and their precision (eg, 95% confidence interval). Make clear which confounders were adjusted for and why they were included **[yes]**
		(*b*) Report category boundaries when continuous variables were categorized **[yes]**
		(*c*) If relevant, consider translating estimates of relative risk into absolute risk for a meaningful time period **[no]**
Other analyses	17	Report other analyses done—eg analyses of subgroups and interactions, and sensitivity analyses **[yes]**
**Discussion**		
Key results	18	Summarise key results with reference to study objectives **[yes]**
Limitations	19	Discuss limitations of the study, taking into account sources of potential bias or imprecision. Discuss both direction and magnitude of any potential bias **[yes]**
Interpretation	20	Give a cautious overall interpretation of results considering objectives, limitations, multiplicity of analyses, results from similar studies, and other relevant evidence **[yes]**
Generalisability	21	Discuss the generalisability (external validity) of the study results **[yes]**
**Other information**		
Funding	22	Give the source of funding and the role of the funders for the present study and, if applicable, for the original study on which the present article is based **[yes]**

*Give information separately for exposed and unexposed groups.

Note: An Explanation and Elaboration article discusses each checklist item and gives methodological background and published examples of transparent reporting. The STROBE checklist is best used in conjunction with this article (freely available on the Web sites of PLoS Medicine at http://www.plosmedicine.org/, Annals of Internal Medicine at http://www.annals.org/, and Epidemiology at http://www.epidem.com/). Information on the STROBE Initiative is available at www.strobe-statement.org

For peer review only - http://bmjopen.bmj.com/site/about/guidelines.xhtml

Appendix F

Swiss Pharmaceutical Market: Number of packaged sold by ATC-groups, 2015

	2015<br>number of packa-ged sold
Total	210,992,389
A Alimentäres System und Stoffwechsel	31,455,252
A01 Stomatologika, Medizinische Präparate zur Mund- und Zahnpflege	1,473,520
A02 Antacida-Ulcustherapheutika, Antiflatulentia	6,051,251
A02A Antacida,Antiflatulentia	1,500,822
A02B Ulcustherapeutika	4,426,496
A02C Sonstige Magentherapeutika	123,933
A03 Produkte gegen funktionelle Magen-Darm-Störungen	2,087,299
A03A Antispasmodika+Anticholinergika rein	595,861
A03C Antispasmodika/Ataraktika-Kombinationen	25,411
A03E Antispasmodika, sonstige Kombinationen	222,102
A03F Gastroprokinetika	1,233,461
A03G Modulatoren der gastrointestinalen Sensomotorik	10,464
A04 Antiemetica und Antinausea	920,841
A05 Cholagoga und Leberschutz-Mittel	337,970
A05A Gallentherapeutica und Cholagoga	254,744
A05B Leberschutzpräparate	83,226
A06 Mittel gegen Verstopfung und Darmreinigung	4,583,118
A06A Mittel Gegen Verstopfung	4,329,037
A06B Darmreinigungsmittel	254,081
A07 Antidiarrhoica, Elektrolytzufuhr und intestinale Antiphlogistica	3,005,035
A07B Intestinale Adsorbierende Antidiarrhoica	228,076
A07E Intestinale Antiphlogistica	222,680
A07F Antidiarrhoica Micro-Organismen	1,340,432
A07G Orale Elektrolyt-Zufuhr	107,030
A07H Motilitätshemmer	1,095,216
A07X Übrige Antidiarrhoica inkl. A07A Antidiarrhoica intestinale Antiinfectiva	11,601
A08 Antiadiposita excl. Diätetica	51,654
A09 Digestiva und Enzyme	804,667
A10 Antidiabetica	3,376,580
A10C Humaninsulin und Analoga	1,078,495
A10H Sulfonylharnstoff Antidiabetika	321,219
A10J Biguanid Antidiabetika	1,217,578
A10K Glitazone Antidiabetika	25,305
A10M Glinide Antidiabetika	38,782
A10N DPP-IV Inhibitor Antidiabetika	483,559
A10P SGLT2-Hemmer Antidiabetika	45,927
A10S GLP-1 Agonisten Antidiabetika	156,656
A10X Übrige Antidiabetika inkl. Insulin tierischen Ursprungs und Alphagluko-sidaseinhibitor Antidiabetika	8,616

1

A11 Vitamine	5,001,408
A11A Multivitamine mit Mineralstoffen	755,243
A11B Multivitamine ohne Mineralstoffe	17,507
A11C Vitamin A+D inkl. einfache Kombinationen	2,618,699
A11D Vitamin B1 und Kombinationen	170,884
A11E Vitamin B-Komplex	739,860
A11F Vitamin B 12 rein	123,445
A11G Vitamin C, inkl. Kombinationen mit Mineralstoffen	353,644
A11X Sonstige Vitamine	222,126
A12 Mineralverbindungen	3,490,891
A12A Mineralverbindung Calcium	1,254,334
A12B Mineralverbindung Kalium	200,438
A12C Sonstige Mineralverbindungen	2,036,119
A13 Tonica und Roborantia	229,735
A16 Übrige Präparate des alimentären und Stoffwechsel-Systems	34,295
B Blut und Blutbildende Organe	5,507,624
B01 Antithrombotika	4,009,465
B01A Vitamin-K-Antagonisten	399,963
B01B Heparine	714,872
B01C Thrombozytenaggregationshemmer	2,265,838
B01D Fibrinolytika	14,293
B01X Direkte Thrombin-Inhibit und direkte Faktor XA-Hemmber	598,766
B02 Blutgerinnungssystem – Sonstige Produkte	237,495
B02B Antagonisten (Antidot.Anticoag)	146,201
B02D Blutcoagulation	1,805
B02G Glitazone-Antidiabetika	48,969
B02X Übrige Blutgerinnungsprodukte (Tissues sealing preparations, Thrombopoietin Agonisten, Antifibrinolytica synthetisch etc.)	40,520
B03 Antianämica	1,259,896
B03A Antianämica mit Eisen und Kombinationen	862,744
B03C Erythropoietin-Präparate	67,258
B03X Sonstige Antianämica inkl. Folsäure/Folinsäure	329,894
C Herz-Kreislauf Therapie	16,027,143
C01 Cardica	954,962
C01B Anti-Arrhythmie-Präparate	213,952
C01C Cardiale Stimulantien excl. Herzglykoside	266,028
C01D Coronartherapeutica excl. Calcium-Antagonisten und Nitropräparate	121,815
C01E Nitropräparate und Analog	228,438
C01X Positive Inotrope, Herzglykoside und übrige Herzpräparate	124,729
C02 Antihypertonica	129,957
C03 Diuretica	1,815,738
C04 Cerebrale und periphere Vasotherapeutika	390,255
C05 Antivaricosa/Antihaemorrhoidalia	2,271,175
C05A Antihaemorrhoidalia topisch	773,800
C05B Antivaricosa topisch	1,029,010
C05C Antivaricosa systemisch	468,365
C06 Übrige Herz+Kreislaufmittel	339,930
C07 Betablocker	2,398,309
C07A Betablocker rein	2,272,988

For peer review only - http://bmjopen.bmj.com/site/about/guidelines.xhtml

C07B Betablocker Kombination	125,321
C08 Calciumantagonisten	1,157,267
C09 Stoffe mit Wirkung auf das Renin-Angiotensin-System	4,116,677
C09A Ace-Hemmer, rein	1,142,465
C09B Ace-Hemmer, Kombinationen	584,697
C09C Angiotensin-II-Antagonisten, rein	1,084,218
C09D Angiotensin-II-Antagonisten, Kombinationen	1,261,616
C09X Sonstige Stoffe mit Wirkung auf das Renin - Angiotensin - System	43,681
C10 Lipidregu/Antiarteriosklerotika	2,446,927
C10A Lipidregulatoren	2,341,963
C10A1 Statine (Cholesterolsynthesehemmer)	2,190,227
C10A2 Lipidsenker, Fibrate, Ionenaustauscher und sonstige Lipidsenker	151,736
C10C Lipidregulatoren kombiniert mit anderen Lipidregulatoren	104,964
C11 Übrige Präparate für die Herz-Kreislauf-Therapie	5,946
D Dermatologica	17,314,810
D01 Dermatologische Antimykotica	1,360,570
D02 Emollientia und Hautschutzmittel	2,703,273
D03 Wundheilmittel	4,389,791
D04 Antipruriginosa inkl. Antihistaminica und Anästhetica usw.	1,458,276
D05 Nichtsteroidale Präparate gegen entzündliche Hauterkrankungen	327,245
D06 Topische antibakterielle Produkte und antivirale Mittel	864,829
D06A Topische antibakterielle Produkte	379,013
D06D Topische Produkte gegen Virusinfektionen	485,816
D07 Corticosteroide topisch	1,982,173
D07A Reine Corticosteroide topisch	960,182
D07B Corticosteroid-Kombinationen topisch	1,021,991
D08 Antiseptica + Desinfizientia	1,836,788
D10 Aknemittel	1,227,850
D10A Topische Aknemittel	829,114
D10B Orale Aknemittel	398,736
D11 Übrige Dermatologica	1,164,015
G Urogenital-System+Sexualhormon	7,936,641
G01 Gynaekologische Antiinfektiva	812,985
G01A Trichomonadenmittel	185,395
G01B Gynaekologische Antimykotica	475,911
G01X Gynaekologische antibakterielle Produkte und Antiseptika	151,679
G02 Sonstige Gynaekologica	1,060,376
G02A Oxytocica	91,111
G02F Topische Sexualhormone	496,735
G02X Lokale Antikonzipientia, Prolactininhibitoren, wehenhemmende Mittel und sonstige Gynaekologika	472,530
G03 Sexualhormone und Stimulantien des Genitalsystems	3,917,793
G03A Hormonale Kontrazeptiva systemisch	2,477,919
G03B Androgene, excl.G3E,G3F	70,852
G03C Oestrogene, excl.G3A,G3E,G3F	397,050
G03D Progestogene, excl. G3A,G3F	245,149
G03F Oestrogen-Progestogen-Kombinationen	487,291
G03G Gonadotropin inl. sonstige Ovulationsstimulantien	100,879

For peer review only - http://bmjopen.bmj.com/site/about/guidelines.xhtml

G03X Androgen-Kombinationen mit weiblichen Sexualhormonen, SERMS und sonstige Sexualhormone und ähnliche Produkte	138,653
G04 Urologica	2,145,487
G04A Urologische Antiinfektiva und Harnantiseptika	410,545
G04C BPH Produkte (Benigne Prostata-Hyperplasie)	715,845
G04D Harninkontinenzprodukte	390,832
G04E Produkte gegen Erektionsstörungen	311,659
G04X Alle sonstigen Urologika	316,606
H Hormonales System, exkl. Sexualhormone	2,875,760
H01 Hypophysen-/Hypothalamus-Hormone	36,944
H02 Corticosteroide Systemisch	1,575,880
H03 Schilddruesentherapie	1,109,934
H04 Sonstige Hormone (wachstumshormone, antidiuretische Hormone, Anti-Parathyroid-Hormone etc.)	153,002
J Antiinfektiva systemisch	8,446,623
J01 System.Antibakter.Prod.	5,721,173
J01A Tetracycline und Kombinationen	155,659
J01C Breitspektrum Penicilline	2,353,136
J01D Cephalosporine	897,839
J01F Macrolide und vergleichbare Substanzen	778,785
J01G Fluorchinolone	817,056
J01H Mittel- und Schmalspektrum-Penicilline	111,484
J01K Aminoglykoside	46,447
J01P Sonstige Beta-Lactam. Antibiot. Penicilline, Cephalosporine (Monobactame, Peneme und Carbapeneme)	69,074
J01X Rifamycin und vergleichbare Substanzen, Trimethoprim, Kombinationen und vergleichbare Substanzen und sonstige antibakterielle Produkte	491,693
J02 Antimykotika, Systemisch	591,346
J04 Tuberkulosemittel	37,320
J05 Antivirale Mittel, systemisch	570,063
J05B Antivirale Mittel, exkl. Produkte gegen HIV	236,676
J05C Virustatika gegen HIV	333,387
J05C1 Nucleoside und Nucleotide Reverse Transkriptase Hemmer	108,107
J05C2 Protease Hemmer	98,581
J05CX Nicht-Nucleoside Reverse Transkriptase Hemmer, HIV Fusionshemmer, HIV-Antivirale Integrase Hemmer und sonst.HIV-Antivirale Mittel	126,699
J06 Sera und Gammaglobulin	23,164
J07 Vakzine	1,406,674
J07B Vakzine Kombinationen	463,631
J07B1 Kombinationen mit Tetanusimpfstoff	387,139
J07BX Mehrfach-Impfstoffe mit Masern und/oder Mumps und alle anderen Kombinationen	76,492
J07D Bakterielle Impfstoffe	164,542
J07E Virale Impfstoffe	687,366
J07E1 Influenza-Impfstoff	205,391
J07E4 Hepatitis-Impfstoff	212,503
J07E9 Alle uebrigen viralen Impfstoffe, inkl. HPV, Rotaviren, Varizellen etc.	269,472
J07X Alle uebrigen Impfstoffe und aehnliche Produkte	91,135
J08B Übrige Antiinfektiva	96,883
K Infusionslösungen	24,158,749
K01 Intravenoese Lösungen ab 100 ML	12,064,154

For peer review only - http://bmjopen.bmj.com/site/about/guidelines.xhtml

K01A Elektrolytlösungen	3,372,213
K01B Standardlösungen	8,461,628
K01D Fettemulsionen, inkl. Produkte zur totalen parenteralen Ernährung	156,484
K01E Aminosäuren-Lösungen	51,140
K01X Kalorische Lös. über 10% und Lösungen zur Osmotherapie	22,689
K02 Plasma Expander	70,547
K03 Blut- und Blutersatzinfusionslösungen	1,817
K04 Injektionslösungen/Infusionszusätze	12,005,621
K04A Elektrolytlösungen	752,499
K04B Standardlösungen	9,981,186
K04C Kalorische Lösungen < 100 ML	34,050
K04D Sonstige Injektionslösungen/Infusionszusätze < 100 ML	1,237,886
K05 Spüllösungen	16,610
L Antineoplastika und Immunmodulatoren	1,934,950
L01 Antineoplastika	984,072
L01A Alkylisierende Substanzen	46,698
L01B Antimetaboliten	323,741
L01C Vinca-Alkaloide & sonstige pflanzliche Produkte	157,793
L01D Antineoplastisch wirkende Antibiotika	46,619
L01F Platinhalt.Antineoplast.	95,347
L01G Monoklonale Antikörper zur antineoplastischen Therapie	159,208
L01H Proteinkinasehemmer zur antineoplastischen Therapie	33,895
L01X Sonstige Antineoplastika	120,771
L02 Cytostatische Hormonetherapie	183,379
L02A Cytostatische Hormone	43,050
L02B Cytostatische Hormonantagonisten	140,329
L03 Immunostimulantien	79,294
L03A Immunstimulantien	40,990
L03B Interferone	38,304
L04 Immunsuppressiva	688,205
L04B Anti-TNF-Produkte	340,857
L04X Sonstige Immunsuppressiva inkl. Interleukin Inhibitoren	347,348
M Muskel-und Skelettsystem	14,787,413
M01 Antirheumatica	6,373,707
M01A Corticoidfreie Antirheumatica	5,700,326
M01C Antirheumatica spezifisch	673,381
M02 Rheumaeinreibungen Rubefatientia	6,372,249
M03 Muskelrelaxantien	665,391
M03A Muskelrelaxantien peripher wirkend	85,757
M03B Muskelrelaxantien zentral wirkend	579,634
M04 Gichtmittel	368,020
M05 Übrigige Muskel-Skelettsystem-Präparate	1,008,046
N Nervensystem	42,690,195
N01 Anaesthetica	1,554,391
N01A Narkosemittel	1,078,794
N01A1 Narkosemittel Inhalationspräparate	20,582
N01A2 Narkosemittel Injektionspräparate	1,058,212
N01B Lokalanaesthetika	475,597

For peer review only - http://bmjopen.bmj.com/site/about/guidelines.xhtml

N01B1 Medizinische injizierbare Lokalanästhetika	198,964
N01B3 Topische Lokalanästhetika	270,962
N01B9 Sonstige Lokalanästhetika und dentale injizierbare Lokalanästhetika	5,671
N02 Analgetica	24,080,469
N02A Betäubungsmittel	1,661,607
N02B Analgetica und Antipyretica	21,918,098
N02B1 Analgetica rezeptpflichtig	8,592,792
N02B2 Analgetica rezeptfrei	13,325,306
N02C Migränemittel	500,764
N02C1 Triptane	432,741
N02C9 Sonstige Migränemittel	68,023
N03 Antiepileptica	1,957,745
N04 Parkinsonmittel	658,710
N05 Psycholeptica	9,075,955
N05A Antipsychotika	2,002,404
N05A1 Atypische Antipsychotika	1,521,618
N05A9 Konventionelle Antipsychotika	480,786
N05B Hypnotica und Sedativa	4,462,062
N05B1 Hypnotica und Sedativa, barbituratfreie Reinsubstanzen	2,830,382
N05B2 Hypnotica und Sedativa, barbituratfreie Kombinationen	129,461
N05B5 Pflanzliche Hypnotica und Sedativa	1,502,219
N05C Tranquilizer	2,611,489
N06 Psychoanaleptica Ex.Antiadipos	4,081,575
N06A Antidepressiva und stimmungsstabilisierende Produkte	3,503,509
N06A2 Pflanzliche Antidepressiva	245,372
N06A3 Stimmungsstabilisierende Produkte	50,396
N06A4 SSRI Antidepressiva	1,319,897
N06A5 SNRI Antidepressiva	664,070
N06A9 Sonstige Antidepressiva	1,223,774
N06B Psychostimulantia	341,172
N06X Psycholeptica-Psychoanaleptica-Kombinationen, Nootropica und Neurotonica sowie verschiedene andere Produkte	236,894
N07 Sonstige ZNS-wirksame Produkte	1,281,350
N07B Raucherentwöhnungsmittel	652,146
N07C Produkte gegen Schwindel	286,538
N07D Anti-Alzheimer Produkte	121,928
N07E Alkohol- und Opiatentwöhnungsmittel	126,973
N07X Alle anderen ZNS-wirksamen Präparate	93,765
P Parasitologie	462,559
P01 Antiprotozoenmittel und Anthelmintika	437,402
P01B Anthelmintica, excl. Schistosomiasis-Mittel	296,327
P01D Malariamittel	139,242
P02 Sonstige Antiparasitäre Präparate inkl. Insektizide und Repellentien	26,990
R Respirationssystem	28,837,468
R01 Rhinologica	7,246,454
R01A Rhinologica topisch	6,868,431
R01AX Corticosteroide Rhinologika	1,067,991
R01A6 Antiallergische Rhinologica	230,180
R01A7 Abschwellende Rhinologica	4,290,641

R01A9 Sonstige Rhinologica topisch inkl. Antiinfektive Rhinologika ohne Corticosteroide	1,279,619
R01B Rhinologica systemisch	378,023
R02 Halsschmerzmittel und Antiinfectiva	3,977,806
R03 Antiasthmatika und Produkte gegen chronisch-obstruktive Lungenerkrankungen	2,931,292
R03A B2-Stimulatoren	923,570
R03B Xanthine	16,188
R03C Degranulationshemmer	9,088
R03D Corticoide	258,075
R03F Komb. B2-Stimulatoren mit Corticoiden	1,172,896
R03G Anticholinergica rein und in Komb. mit B2-Stimulatoren	422,479
R03G3 Anticholinergica rein, Inhalate	269,418
R03G4 Anticholinergica – Kombinationen mit Beta-2-Stimulantien, Inhalate	153,061
R03J Antileukotrien Antiasthmatika	90,062
R03X Sonstige Antiasthmatika und Produkte gegen chronisch-obstruktive Lungenerkrankungen inkl. PDE4-Hemmer gegen Asthma / Chronisch-Obstruktive Lungenerkrankungen	38,934
R04 Percutane Einreibemittel und Inhalationspräparate	1,093,346
R05 Husten- und Erkältungspräparate	10,899,026
R05A Erkältungspräparate	2,498,594
R05C Expectorantia ohne Antiinfectiva	4,499,364
R05D Hustensedativa	3,297,144
R05D1 Hustenmittel plain (Einzelsubstanzen)	1,971,766
R05D2 Sonstige Hustensedativa, inkl. Kombinationen	1,325,378
R05F Sonstige Husten- und Erkältungspräparate	603,924
R06 Antihistamine systemisch	2,687,483
R07 Sonstige Präparate des Respirationssystems	2,061
S Sinnesorgane	7,658,311
S01 Ophthalmologica	7,214,097
S01A Ophtalmologische Antiinfectiva	451,604
S01B Ophtalmologische Corticoide rein	271,663
S01C Ophthalm. Antiphlogistika und Antiinfektiva Kombinationen	613,682
S01E Myotika und Mittel zur Glaukombehandlung	1,038,592
S01F Mydriatika und Cykloplegika	46,547
S01G Ophthalmologische Antiallergika, Abschwellende Mittel, Antiseptika	1,473,188
S01H Ophtalmolog. Lokalanesthetika	40,383
S01K Künstliche Tränen und Netzmittel für Augen	2,503,750
S01P Ophthalmologische Antineovaskularisationsprodukte	127,501
S01R Nonsteroidale entzündungshemmende Ophthalmologica (NSAID'S)	236,207
S01X Übrige Ophtalmologica inkl. Präparate für den Gebrauch von Kontaktlinsen, Augentonika und Augenvitamine, Präparate zur Verhütung von Katarakt und Antikataraktogenika, Ophthalmologische Operationshilfsmittel und Ophthalmologische Diagnostika	411,009
S02 Otologica	444,214
T Diagnostika	43,184
T01 Diagnostika für bildgebende Verfahren	18,885
T02 Testdiagnostika	24,299
V Verschiedenes	855,707
V01 Allergene	6,429
V03 Übrige Therapeutische Präparate	820,500

For peer review only - http://bmjopen.bmj.com/site/about/guidelines.xhtml

V03D Entgiftungspräparate für die Cytostatikatherapie	45,668
V03E Antidote	4,532
V03G Hyperkaliämie/Hyperphosphatämie-Produkte	42,259
V03X Sonstige therapeutische Präparate inkl. Entzündungshemmende Enzyme, Eisen-Chelatbildner und Radiopharmazeutika	728,041
V05 Chirurgische Antiseptica	134
V06 Allgemeine Nährmittel	5,612
V07 Alle übrigen nicht therapeutischen Präparate	23,032

Source: Interpharma with data from IMS Health Schweiz, 2016.

For peer review only - http://bmjopen.bmj.com/site/about/guidelines.xhtml

STROBE Statement—Checklist of items that should be included in reports of ***cross-sectional studies***

	Item No	Recommendation	Manu-script page
**Title and abstract**	1	(*a*) Indicate the study's design with a commonly used term in the title or the abstract **[yes]**	1
		(*b*) Provide in the abstract an informative and balanced summary of what was done and what was found **[yes]**	1-2
**Introduction**			
Background/rationale	2	Explain the scientific background and rationale for the investigation being reported **[yes]**	3
Objectives	3	State specific objectives, **[yes]** including any prespecified hypotheses **[no]**	3
**Methods**			
Study design	4	Present key elements of study design early in the paper **[yes]**	3
Setting	5	Describe the setting, locations, and relevant dates, including periods of recruitment, exposure, follow-up, and data collection **[yes]**	3
Participants	6	(*a*) Give the eligibility criteria, and the sources and methods of selection of participants **[yes]**	3
Variables	7	Clearly define all outcomes, exposures, predictors, potential confounders, and effect modifiers. **[yes]** Give diagnostic criteria, if applicable **[no]**	3
Data sources/ measurement	8*	For each variable of interest, give sources of data and details of methods of assessment (measurement). Describe comparability of assessment methods if there is more than one group **[yes]**	4
Bias	9	Describe any efforts to address potential sources of bias **[yes]**	4
Study size	10	Explain how the study size was arrived at **[yes]**	4-5
Quantitative variables	11	Explain how quantitative variables were handled in the analyses. If applicable, describe which groupings were chosen and why **[yes]**	4-5
Statistical methods	12	(*a*) Describe all statistical methods, including those used to control for confounding **[yes]**	5
		(*b*) Describe any methods used to examine subgroups and interactions **[yes]**	5
		(*c*) Explain how missing data were addressed **[yes]**	5
		(*d*) If applicable, describe analytical methods taking	n.a.

For peer review only

		account of sampling strategy [no]	
		(*e*) Describe any sensitivity analyses [no]	n.a.
**Results**			
Participants	13*	(a) Report numbers of individuals at each stage of study—eg numbers potentially eligible, examined for eligibility, confirmed eligible, included in the study, completing follow-up, and analysed [no]	5
		(b) Give reasons for non-participation at each stage [yes]	5
		(c) Consider use of a flow diagram [yes]	18
Descriptive data	14*	(a) Give characteristics of study participants (eg demographic, clinical, social) and information on exposures and potential confounders [yes]	22-24
		(b) Indicate number of participants with missing data for each variable of interest [yes]	5
Outcome data	15*	Report numbers of outcome events or summary measures [yes]	6
Main results	16	(*a*) Give unadjusted estimates and, if applicable, confounder-adjusted estimates and their precision (eg, 95% confidence interval). Make clear which confounders were adjusted for and why they were included [yes]	28
		(*b*) Report category boundaries when continuous variables were categorized n.a.	n.a.
		(*c*) If relevant, consider translating estimates of relative risk into absolute risk for a meaningful time period [no]	n.a.
Other analyses	17	Report other analyses done—eg analyses of subgroups and interactions, and sensitivity analyses [yes]	24
**Discussion**			
Key results	18	Summarise key results with reference to study objectives [yes]	8
Limitations	19	Discuss limitations of the study, taking into account sources of potential bias or imprecision. Discuss both direction and magnitude of any potential bias [yes]	2
Interpretation	20	Give a cautious overall interpretation of results considering objectives, limitations, multiplicity of analyses, results from similar studies, and other relevant evidence [yes]	8-11
Generalisability	21	Discuss the generalisability (external validity) of the study results [yes]	6
**Other information**			

For peer review only

Funding	22	Give the source of funding and the role of the funders for the present study and, if applicable, for the original study on which the present article is based **[yes]**	13

*Give information separately for exposed and unexposed groups.

Note: An Explanation and Elaboration article discusses each checklist item and gives methodological background and published examples of transparent reporting. The STROBE checklist is best used in conjunction with this article (freely available on the Web sites of PLoS Medicine at http://www.plosmedicine.org/, Annals of Internal Medicine at http://www.annals.org/, and Epidemiology at http://www.epidem.com/). Information on the STROBE Initiative is available at www.strobe-statement.org

For peer review only
